# Comparative metabolomics analysis reveals alkaloid repertoires in young and mature *Mitragyna speciosa* (Korth.) Havil. Leaves

**DOI:** 10.1371/journal.pone.0283147

**Published:** 2023-03-21

**Authors:** Rubashiny Veeramohan, Arief Izzairy Zamani, Kamalrul Azlan Azizan, Hoe-Han Goh, Wan Mohd Aizat, Mohd Fauzi Abd Razak, Nur Sabrina Mohd Yusof, Sharif Mahsufi Mansor, Syarul Nataqain Baharum, Chyan Leong Ng

**Affiliations:** 1 Institute of Systems Biology, Universiti Kebangsaan Malaysia, UKM Bangi, Selangor, Malaysia; 2 Leave a Nest Malaysia Sdn Bhd, Cyberjaya, Selangor, Malaysia; 3 Centre for Drug Research, Universiti Sains Malaysia, Minden, Penang, Malaysia; Bangabandhu Sheikh Mujibur Rahman Agricultural University, BANGLADESH

## Abstract

The fresh leaves of *Mitragyna speciosa* (Korth.) Havil. have been traditionally consumed for centuries in Southeast Asia for its healing properties. Although the alkaloids of *M*. *speciosa* have been studied since the 1920s, comparative and systematic studies of metabolite composition based on different leaf maturity levels are still lacking. This study assessed the secondary metabolite composition in two different leaf stages (young and mature) of *M*. *speciosa*, using an untargeted liquid chromatography-electrospray ionisation-time-of-flight-mass spectrometry (LC-ESI-TOF-MS) metabolite profiling. The results revealed 86 putatively annotated metabolite features (RT:m/z value) comprising 63 alkaloids, 10 flavonoids, 6 terpenoids, 3 phenylpropanoids, and 1 of each carboxylic acid, glucoside, phenol, and phenolic aldehyde. The alkaloid features were further categorised into 14 subclasses, i.e., the most abundant class of secondary metabolites identified. As per previous reports, indole alkaloids are the most abundant alkaloid subclass in *M*. *speciosa*. The result of multivariate analysis (MVA) using principal component analysis (PCA) showed a clear separation of 92.8% between the young and mature leaf samples, indicating a high variance in metabolite levels between them. Akuammidine, alstonine, tryptamine, and yohimbine were tentatively identified among the many new alkaloids reported in this study, depicting the diverse biological activities of *M*. *speciosa*. Besides delving into the knowledge of metabolite distribution in different leaf stages, these findings have extended the current alkaloid repository of *M*. *speciosa* for a better understanding of its pharmaceutical potential.

## Introduction

The *Mitragyna* genus from the Rubiaceae family encompasses 10 species, of which six are Asian and four are African. The most prevalent species in the Malay Peninsula are *Mitragyna speciosa*, *Mitragyna diversifolia*, *Mitragyna hirsuta*, *Mitragyna parvifolia*, *Mitragyna rotundifolia*, and *Mitragyna tubulosa*, which are known to contain indole alkaloids with pharmacological properties [1,2]. Among the species, *M*. *speciosa* has the most documented narcotic properties as an opium substitute with controversial debate on its legal usage and potential abuse. Furthermore, it is easily obtained through the internet in many Western countries like the United Kingdom (UK) and the United States (US) [3–5] and certain Asian countries like Japan [3,6]. *M*. *speciosa* is widely grown in Southeast Asian nations such as Indonesia, Malaysia, and Thailand, mostly for its leaves [7,8]. Indonesia is known to cultivate *M*. *speciosa* for global exportation, especially to Europe and North America [9,10]. In Malaysia, the trees of *M*. *speciosa* are often grown by villagers in their backyards for consumption [11,12]. It is generally known as *kratom* in Thailand and *ketum* or *biak-biak* in Malaysia. The fresh mature leaves of *M*. *speciosa* have been traditionally utilised for therapeutic purposes [13] by chewing or consumed as tea for stimulating effects that increase energy and work productivity [11,14,15]. It is also widely used in Southeast Asian countries as an aphrodisiac, to improve blood circulation, to endure physical fatigue, and to treat diarrhoea, fever, diabetes, chronic pain, and opiate withdrawal syndrome [12,15–19]. The leaf extracts of *M*. *speciosa* have been reported to show various biological activities, including antibacterial, antioxidant [20], antimutagenic [21], anti-inflammatory [22], antitussive [23], anaesthetic [24], antipsychotic [25], and antinociceptive [22,26,27] effects. These pharmacological actions are mostly linked to alkaloids in the extracts. However, the pharmacological and safety profiles of *M*. *speciosa* remain poorly understood and warrant further investigations [11].

To date, at least 58 alkaloids have been reported in different plant organs (leaf, bark, stem bark, stem, root, fruit, etc.), since the 1920s (S1 Table). Mitragynine (MG) was the first alkaloid to be isolated [28], followed by mitraspecine [29], and the rest were identified between 1963 to 2020. Most pharmacological studies of *M*. *speciosa* constituents have been extensively focused on MG and 7-hydroxymitragynine (7-OHMG), known as opioid antinociceptive agents [30–32]. MG and 7-OHMG function as partial agonists of the human mu (μ)-opioid receptor, which also acts on kappa (κ)- and delta (δ)-opioid receptors as competitive antagonists [33,34]. 7-OHMG was shown to be 46- and 13-fold more potent than MG and morphine, exhibiting greater affinity for the μ-opioid receptor [35]. Moreover, 7-OHMG was proposed to pose a higher risk of addiction and toxicity with *M*. *speciosa* consumption [36,37].

Previous investigations on the distribution of indole and oxindole alkaloids in *M*. *speciosa* using thin-layer chromatography (TLC) have shown that the occurrence and abundance of alkaloids vary between young plants and old trees, different organs (leaf, twig, stem bark, and root bark), as well as locality and time of collection [38–40]. Other factors that influence the variability in *M*. *speciosa* constituents are environmental factors [41], variety [42], and leaf maturity [43]. However, Houghton et al. [43] collected the young and mature leaf samples from different geographical areas of Malaysia; young leaves were collected from trees grown in Selangor, whereas mature leaves were collected from Perlis. Four isolated alkaloids, mitragynaline, corynantheidinaline, mitragynalinic acid, and corynantheidinalinic acid, were more abundant in young leaves, with a minute amount in mature leaves [43]. Furthermore, many other studies focused on investigating the alkaloids in either mature leaves [44–46], commercial products [47,48], or leaf samples of unspecified age [7,49,50]. Although biochemical and physiological characteristics between young and mature leaves generally vary [51–55], systematic comparison studies on the influence of different leaf stages on metabolite composition and abundance are limited. As the constituents of *M*. *speciosa* can also vary geographically and with sampling time [39], it is necessary to conduct sampling at the same locality and time point. In this study, a systematic study was employed using an untargeted metabolomics approach of liquid chromatography-electrospray ionisation-time-of-flight-mass spectrometry (LC-ESI-TOF-MS) to compare the composition and abundance of secondary metabolites in the young and mature leaves of *M*. *speciosa*.

To date, only a few targeted indole and oxindole alkaloids were already isolated and characterised from *M*. *speciosa*. Enhanced metabolite identifications using nuclear magnetic resonance (NMR) [7,47,49], gas chromatography-mass spectrometry (GC-MS) [42,56,57], high-performance liquid chromatography (HPLC) [42], and liquid chromatography-mass spectrometry (LC-MS) [50,57,58] were performed on *M*. *speciosa* leaf samples from various locations, with the isolated and characterised metabolites mostly targeted. Phytochemical characterisation of *M*. *speciosa* leaves was conducted using NMR and TLC, which isolated 18 compounds [41] without distinguishing different leaf tissues. At the current stage, apart from the targeted metabolites, a complete metabolite profile of the plant is yet to be studied. On this basis, untargeted metabolite profiling using LC-ESI-MS serves as a practical tool for finding bioactive compounds by analysing the metabolites in plant extracts and linking them to their biological activities [59,60]. Only one untargeted LC-MS metabolomics was employed recently to profile 53 commercial kratom products in the US to determine alkaloid variations, followed by targeted studies of MG, 7-OHMG, and speciofoline with *in vitro* evaluation of their biological effects [48]. However, annotation of all the overall metabolites was not reported. To our knowledge, this study presents the first systematic metabolite profiling and comparative analysis of young and mature *M*. *speciosa* leaves. The findings will substantially enhance existing knowledge of *M*. *speciosa* leaves and set the groundwork for subsequent research on this plant.

## Materials and methods

### Chemicals and reagents

Analytical-grade methanol (CH_3_OH) was acquired from Merck, Germany, while umbelliferone (C_9_H_6_O_3_, purity 99%) was acquired from Sigma-Aldrich, St. Louis, USA. Deionised water was filtered using the Milli-Q Reagent Water System (Millipore Billerica, MA, USA). Mitragynine (C_23_H_30_N_2_O_4_, purity ≥ 95%) and 7-hydroxymitragynine (C_23_H_30_N_2_O_5_, purity 97.9%) reference standards were obtained from Cayman Chemical (Ann Arbor, Michigan, USA) and Cerilliant (Round Rock, Texas, USA).

### Plant materials

Young (freshly expanding top two leaves from the shoot tip) and mature (seventh to tenth leaves from the top) leaves (S1 Fig) were obtained from the Centre for Drug Research (CDR), Universiti Sains Malaysia (USM), Penang. The leaves were collected from the same tree at the same time point at Kuala Kedah. The plant was identified by Dr Farah Alia, from Universiti Sains Malaysia (USM), and a voucher specimen number 11869 was deposited at the herbarium of the School of Biological Sciences, USM. The authentication of plant material is included in the supplementary materials, and the results are summarised in the S1 Text. The leaves of *M*. *speciosa* were flash-frozen using liquid nitrogen and kept in a -80°C freezer for metabolite extractions.

### Metabolite extraction

Sample extraction was performed as previously described [61]. The leaves were individually pulverised in liquid nitrogen with mortar and pestle prior to weighing and put into separate Falcon tubes. About 100 mg of powdered samples were immersed in freshly made ice-cold methanol (5mL), vortexed, and incubated for 8 hours on dry ice before overnight incubation in a high-capacity incubator shaker at 20°C. The mixture was centrifuged at 4°C and 6,000 rpm for 10 min. Next, a 0.2-μm polytetrafluoroethylene (PTFE) syringe filter was used to filter the supernatant. To prevent degradation, 1 mL of the extract was pipetted into vials and kept in a -80°C freezer. Later, the extracts were spiked with an internal standard (100 ppm of umbelliferone) before LC-MS analysis [61]. An internal standard is crucial in metabolite profiling studies because it serves as a reference for relative quantification and validation of chromatographic and MS system performance [62–64]. Pooled young (2–3 leaves) and individual mature leaf samples were used to prepare five biological and five technical replicates.

### LC-ESI-TOF-MS analysis

A Thermo Scientific C18 column (AcclaimTM Polar Advantage II, 3 × 150 mm, 3 μm particle size) on an Ultimate UHPLC system (Dionex) was used to perform chromatographic separation of *M*. *speciosa* leaf extracts as described previously [61]. Gradient elution with mobile phases of 0.1% formic acid in water (A) and 100% acetonitrile (ACN, B) was performed at 40°C with a flow rate of 0.4 mL/min. The total run time was 15 min. A sample injection volume of 1 μL was used, and the gradient was initiated at 5% solvent B (0–0.5 min), increased to 90% solvent B (0.5–6 min) and maintained at 90% solvent B (6–10 min). The gradient was then returned to 5% solvent B (10–12 min) and finally maintained at 5% solvent B (12–15 min). A MicroTOF-Q III Bruker Daltonics was used to perform high-resolution mass spectrometry in positive ionisation mode with electrospray ionisation (ESI) source settings of 4,500 V capillary voltage, 1.2 bar of nebuliser pressure, 8 L/min of drying gas flow rate at 200°C, and mass range of scan spectra from 50 to 1000 m/z.

MS/MS analysis for young and mature leaf samples of *M*. *speciosa* was done according to Rosli et al. [62] by pooling replicates of all extracts at equal amounts. Tandem mass spectra were acquired in Auto-MS/MS and multiple reaction monitoring (MRM) mode to facilitate compound identification.

### LC-MS data processing

The LC-MS raw data was processed as described by Veeramohan et al. [61] using Bruker Compass DataAnalysis version 4.1 (Bruker Daltonic GmbH) for peak detection and deconvolution of the total ion current chromatogram (TIC). This subsequently generated a list of retention time (RT) to mass per charge ratio (m/z) peaks linked to the detected compounds and intensity values via Find Molecular Features (FMF) algorithm [65]. The processed data was then aligned using Bruker Compass ProfileAnalysis version 2.1 (Bruker Daltonic, Germany) for bucket generation. Next, the generated dataset was tabulated and changed to.xlsx and.csv formats for subsequent analysis. Each bucket (peak) in the table represents a metabolite feature (RT:m/z value; m/z value up to three decimal places as default setting by the software), representing a metabolite. Data filtering was conducted by filtering out metabolite features that are not present in at least 50% of the samples in at least one leaf age group. Missing intensity values for several features in the dataset were manually added via manual peak picking. Furthermore, metabolite features with a coefficient of variation (CV) of > 30% in both leaf age groups were filtered out before metabolite profiling, relative quantification, and statistical analysis to minimise intensity value variation between technical replicates.

For the Auto-MS/MS data, raw data were analysed using DataAnalysis Viewer 4.2 (Bruker Daltonics) to visualise the fragmentation pattern of each RT:m/z value pairs detected. Targeted metabolite features were subjected to MRM mode to obtain their fragmentation profiles. The acquisition parameters of the targeted metabolites are shown in S2 Table.

### Identification of metabolites

The RT:m/z values detected in at least three out of five biological replicates in either group were selected for annotation. Metabolite identification for LC-MS data was attained by employing searches based on mass (m/z values), followed by manual verification similar to Rosli et al. [62], with some modifications. The m/z values were looked up in previous studies and online databases, such as METLIN [66], MetFrag [67], MassBank [68], and KNApSAcK [69]. All metabolite annotations were based on only protonated molecule ions of [M+H]^+^. Metabolites from the Auto-MS/MS and MRM data were identified based on the m/z value, RT in min, molecular formula, and fragmentation profiles. Since several metabolite databases were searched to identify candidates, isomeric features may match numerous candidates [70]. Hence, only metabolites with molecular weights within a 20 ppm mass error of the query m/z value were acquired and annotated from the databases [62] to decrease the number of candidates and increase the confidence in identification. Although these databases help with annotations, it is not always possible to narrow down the results of an observed metabolite feature to a single candidate. Therefore, multiple annotations are provided for such metabolite features in this manuscript. Additionally, the identities of MG and 7-OHMG were validated with authentic standards. Chromatograms of MG and 7-OHMG are shown in S2 Fig.

The level of identification (ID level) for the metabolites identified in this study was determined according to the criteria previously disclosed with some changes [71,72]. Level 1 was attributed to the metabolites validated with authentic standards, whereas level 2 was assigned to putatively identified metabolites with fragmentation profiles. Level 3 was assigned to putatively identified metabolites using parent ions due to the absence of fragmentation profiles. Exact m/z values (up to 4 decimal places) were reported for metabolite features identified with levels 1 and 2, while m/z values up to 3 decimal places (as a default setting by ProfileAnalysis software) were reported for metabolite features annotated with level 3 identification.

### Statistical analyses and relative quantification

MetaboAnalyst 5.0 [73] was used for peak intensity data normalisation by reference feature (internal standard), log transformation, and Pareto scaling, along with statistical analysis (fold change analysis [|Log_2_FC| > 2] and t-test [false discovery rate (FDR)-adjusted p-value < 0.05]). FDR correction was automatically done in MetaboAnalyst 5.0. Normalisation by reference feature was caried out to account for systematic variations between samples. Log transformation and Pareto scaling were applied to reduce high variations of intensity values between metabolite features. Multivariate analysis by unsupervised PCA and supervised partial least squares discriminant analysis (PLS-DA) was executed via SIMCA-P 14.1 (Umetrics, Sweden) using the normalised data generated by MetaboAnalyst 5.0. Metabolites were further organised according to their significance in projecting the variations in the PLS-DA model. Metabolites that significantly contribute to the discrimination of young and mature leaf groups were shown to have variable importance in the projection (VIP) values > 1.0. The Venn diagram was created using an online application (http://bioinformatics.psb.ugent.be/webtools/Venn/).

The relative abundance of metabolites was calculated using a semi-quantitative method [74,75]. The signal intensities of each putative metabolite were divided by the signal intensity values of the spiked internal standard (umbelliferone, 100 mg/L) from each biological and technical replicate. Means were calculated from each sample replicate and denoted as relative abundance. A heatmap denoting the relative abundance of putatively identified metabolites across young and mature leaves of *M*. *speciosa* was generated using MetaboAnalyst 5.0 [73]. The comparative relative abundance of each putatively identified indole alkaloid was graphed using GraphPad Prism 7 (GraphPad Software, Inc.). The mean for each group with standard error of the mean (SEM) was displayed in each graph.

## Results

### Overall metabolites in *M*. *speciosa*

**Fig 1:**
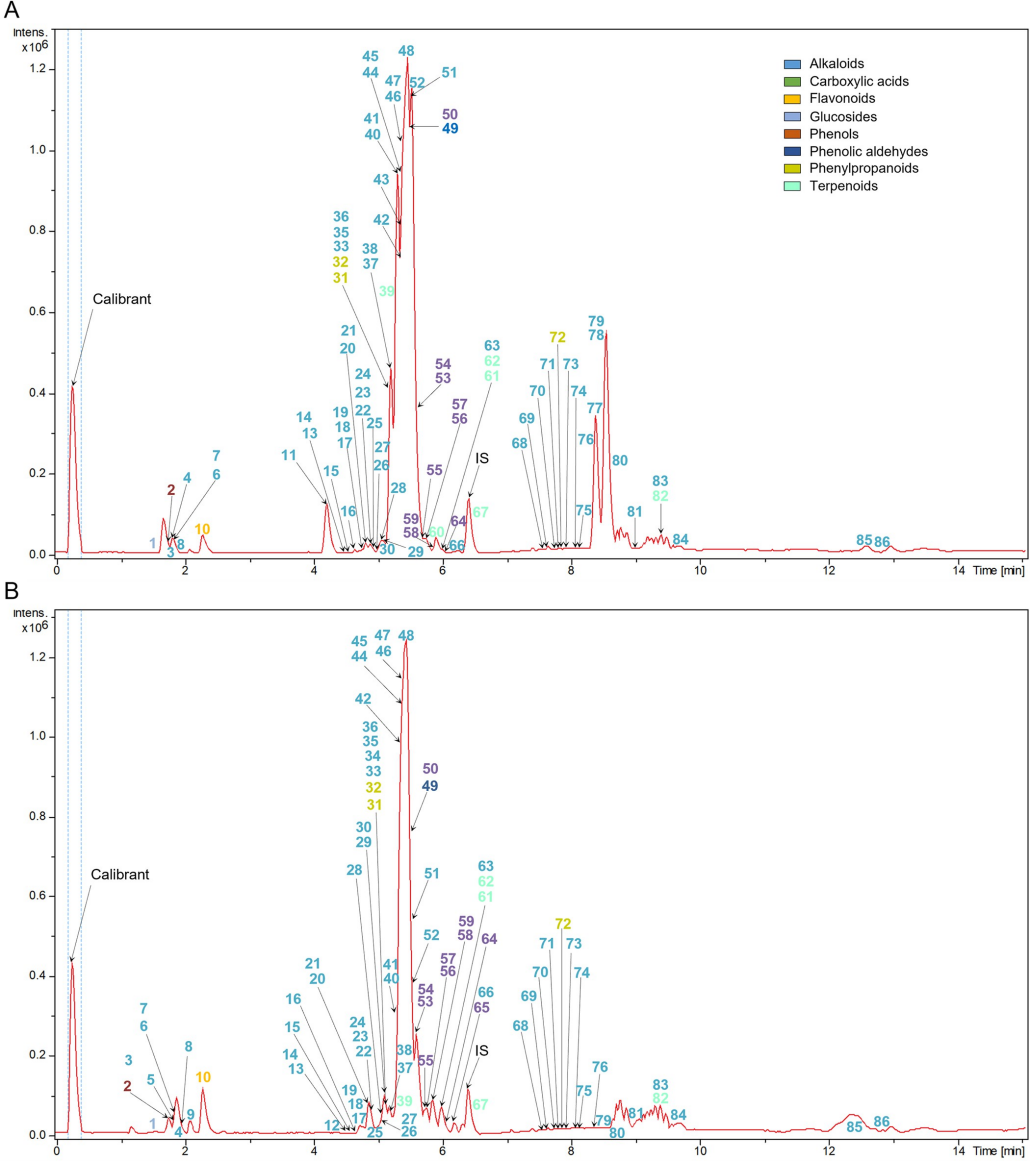
The representative base peak chromatograms (BPC) of young (A) and mature (B) *M*. *speciosa* leaf methanol extract obtained by LC-ESI-TOF-MS. Peak numbering designates the identified compounds.

Untargeted LC-ESI-TOF-MS profiling was performed to obtain metabolite features (RT:m/z values) corresponding to various compounds in young and mature leaves of *M*. *speciosa*. The predominant ion was the molecular ion ([M+H]^+^, m/z 399.2286) of alkaloid MG (Fig 1).

The metabolites of the young and mature *M*. *speciosa* leaves were putatively identified using LC-ESI-TOF-MS, tandem mass spectrometry, previous studies, and online databases. The results revealed a total of 86 metabolites putatively identified in young and mature leaves of *M*. *speciosa* (S3 and S4 Tables). They were further categorised into different classes of secondary metabolites, including 63 alkaloids (S3 Table) and 23 other secondary metabolites consisting of a carboxylic acid, a glucoside, a phenol, 3 phenylpropanoids, 6 terpenoids, 10 flavonoids, and a phenolic aldehyde (S4 Table). Alkaloids make up most of the total metabolites in *M*. *speciosa*, followed by flavonoids and terpenoids.

**Fig 2:**
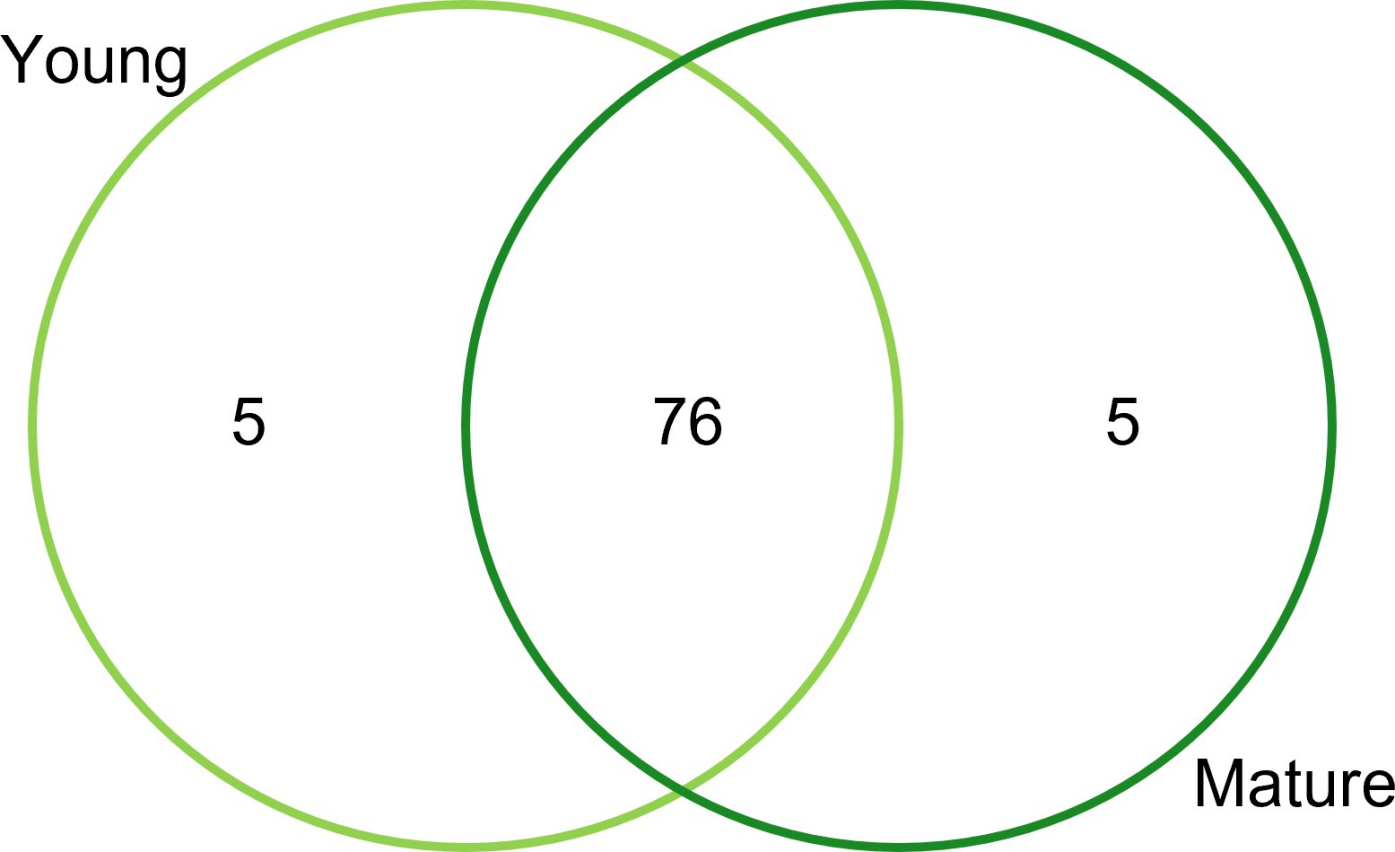
Venn diagram representing the number of overall identified metabolite features shared between or unique to young and mature leaves of *M*. *speciosa*.

The Venn diagram illustrates the uniqueness and overlapping of the overall identified metabolite features among young and mature leaves of *M*. *speciosa* (Fig 2). Both young and matured leaves shared high similarity (76 metabolites) in the metabolite profiles. Five metabolites are only found in young leaves, while another five metabolites are uniquely present in mature leaves (Fig 2). The five metabolites uniquely present in young leaves include four alkaloids and one terpenoid, while the five exclusively present in mature leaves include four alkaloids and one flavonoid. The distribution of each metabolite feature can be found in S3 and S4 Tables.

### Metabolomics revealed differences in metabolite composition between young and mature leaves of *M*. *speciosa*

**Fig 3:**
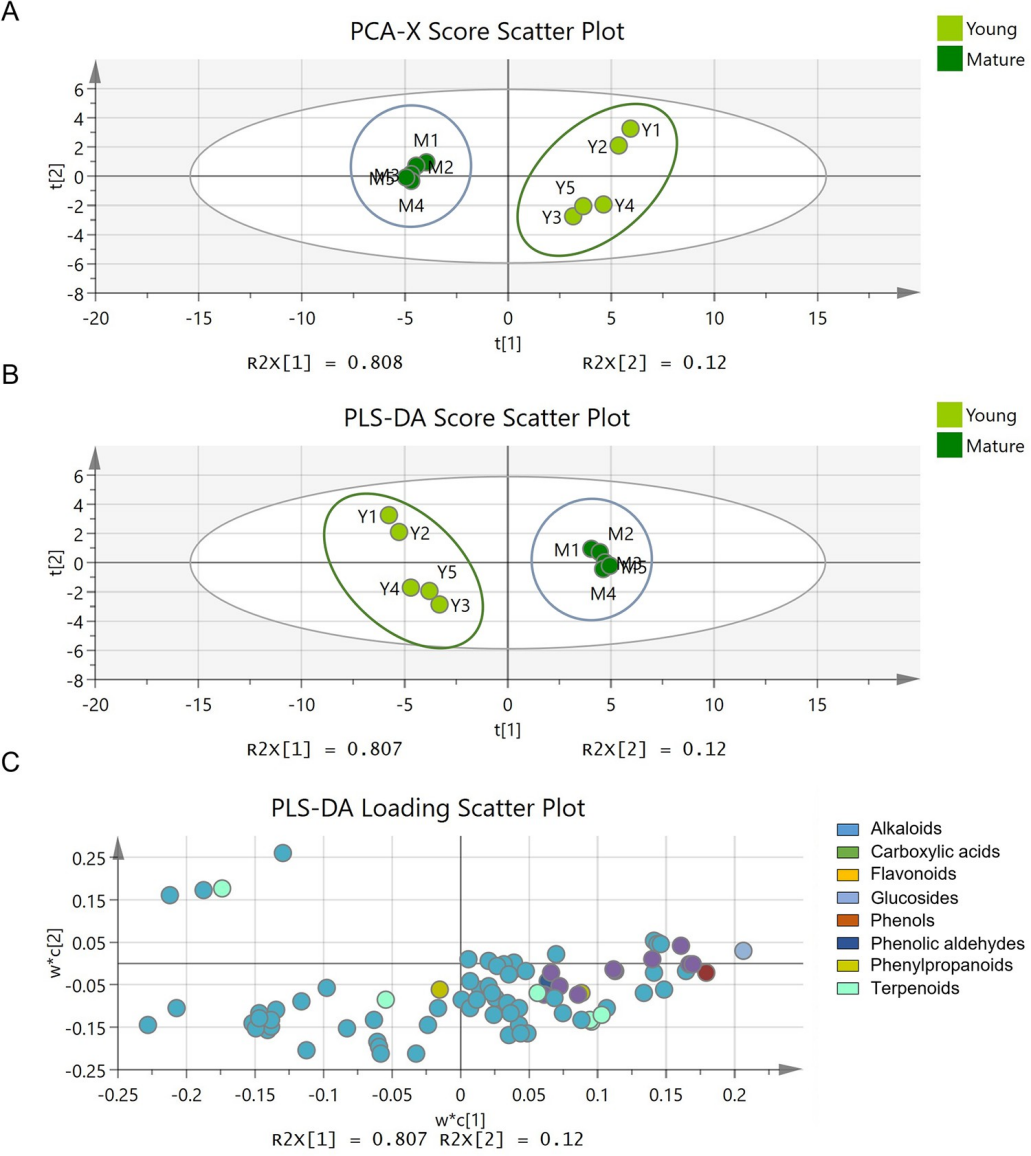
Multivariate analysis of *M*. *speciosa*’s metabolite profile. (A) PCA score scatter plot of young (lime green) and mature (dark green) leaves in five biological replicates. Circles are labelled relative to the leaf age groups. (B) PLS-DA score scatter plot of young and mature leaves in five biological replicates. Circles are labelled relative to the leaf age groups. (C) PLS-DA loading scatter plot projecting metabolite features that influenced the clustering and separation of young and mature leaf groups.

To analyse the differences in metabolite composition between young and mature leaves, the normalised data matrices acquired from the LC-ESI-TOF-MS analysis were subjected to MVA. With each point denoting a distinct sample, an unsupervised PCA displays the projections of each sample in a multidimensional space. The differences in metabolite compositions are connected to the sample dispersions, and samples with greater similarities are located together, while samples with greater differences are located farther away [62]. A score plot clusters samples according to their metabolite composition, whereas a loading plot represents the metabolites contributing to the variances amongst samples on the score plot [76]. The PCA score plot in Fig 3A shows that the young (Y1–Y5) and mature (M1–M5) groups formed discrete clusters, separated from one another with a total variance (R2X[cumulative] and Q2[cumulative]) of 92.8% and 87.9%.

A supervised PLS-DA was used to identify metabolite features that vary significantly between young and mature leaves. The PLS-DA score plot shows that the difference between young and mature leaf metabolome was separated with a good fit of R2X(cumulative) of 92.8%, R2Y(cumulative) of 99.7%, and Q2(cumulative) of 99.4% (Fig 3B). The PLS-DA loading scatter plot (Fig 3C) shows the distribution of metabolite features corresponding to the separation of the leaf samples in the PLS-DA score scatter plot (Fig 3B), where the metabolites projected further from the centre contribute more to the separation. Subsequently, variable importance in projection (VIP) scores were used to identify metabolite features contributing to the discrimination between young and mature leaf samples. A total of 34 metabolite features with VIP > 1.0 were identified and included in the S3 and S4 Tables. Metabolites with the greatest influence include 11-methoxy-vinorine (alkaloid), vomicine (alkaloid), hirsuteine (alkaloid), 5′′-O-β-D-glucosylpyridoxine (glucoside), and 3-methoxytyramine-betaxanthine (phenol).

**Fig 4:**
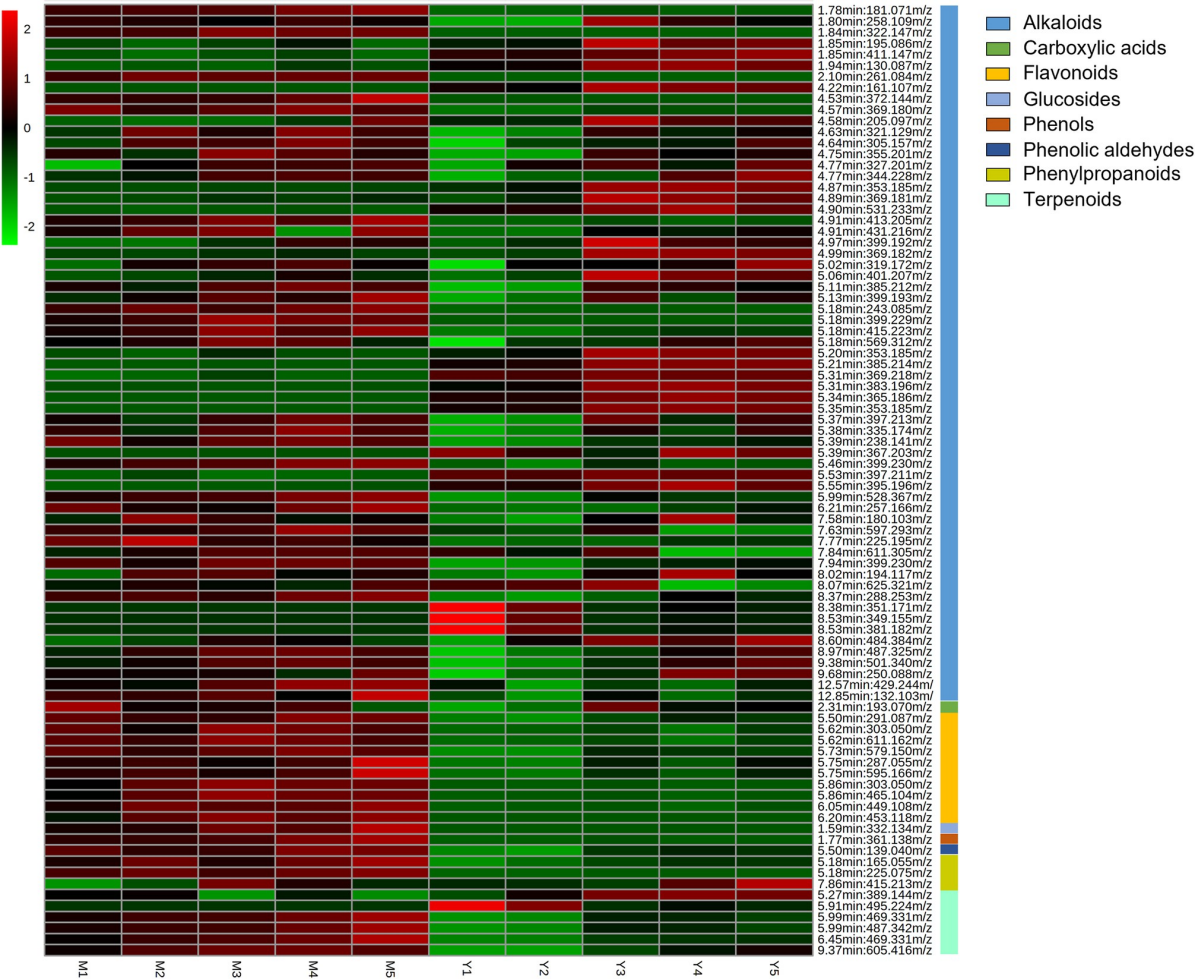
Heatmap showing the relative abundance of 86 metabolite features [RT(min):m/z] in young and mature leaves of *M*. *speciosa*. The scale bar of the heatmap indicates the relative abundance of the annotated metabolite features, with the lighter green colour representing lower intensity and the brighter red colour representing higher intensity. Annotation of each metabolite feature can be found in the S3 and S4 Tables.

Additionally, a heatmap is used to visualise the metabolite expression of all 86 metabolite features annotated to their respective secondary metabolite classes (Fig 4). The heatmap demonstrates varying expression levels of metabolites between young (Y1–Y5) and mature (M1–M5) leaves. Most secondary metabolites, including alkaloids, carboxylic acid, flavonoids, glucoside, phenol, phenolic aldehyde, phenylpropanoids, and terpenoids in *M*. *speciosa* showed higher expression in mature leaves (up-regulation) compared to the young ones. Conversely, 26 metabolite features annotated as alkaloids, a phenylpropanoid, and 2 terpenoids were observed to show higher expression in young leaves (down-regulation) compared to the mature leaves (Fig 4).

### *M*. *speciosa* leaves contain alkaloids of various subclasses

A total of 57 annotated alkaloid features were further categorised into 14 subclasses, i.e., indole, quinoline, isoquinoline, quinolizidine, quinazoline, terpenoid, cyclopeptide, peptide, harmala, phenanthridine, piperamide, piperidine, purine, and pyrazine alkaloids. Meanwhile, six metabolite features were categorised as unclassified due to several annotation hits from different alkaloid subclasses for similar metabolite features. For example, the metabolite feature m/z 225.195 at RT 7.77 is annotated to anapheline (piperidine alkaloid) and cuscohygrine (pyrrolidine alkaloid), with the same mass error (Δppm -5.1; S3 Table). Indole alkaloids are the major subclass, with 30 metabolite features putatively identified (ID level 2 and 3) and 2 metabolite features validated with authentic standards (ID level 1), i.e., MG and 7-OHMG (S2 Fig), followed by 6 isoquinolines, 4 quinolines, 3 piperidines, 2 metabolite features in each subclass of cyclopeptides and purines, 1 metabolite feature in the other remaining subclasses, and 6 unclassified alkaloids (S3 Table). It is noteworthy that most of these alkaloid subclasses have not been reported in *M*. *speciosa* thus far. These results putatively revealed 46 more alkaloids than the previously reported list (S1 Table) (14 alkaloids putatively identified with ID level 2 and 32 alkaloids annotated with ID level 3). From the 58 alkaloids previously reported in *M*. *speciosa*, 17 metabolite features in this study are annotated to the known indole alkaloids (MG, 7-OHMG, speciogynine, corynantheidine, paynantheine, isopaynantheine, speciociliatine, and ajmalicine) and oxindole alkaloids (mitraphylline, isomitraphylline, rynchophylline, isorynchophylline, corynoxine, corynoxine B, speciofoline, isospeciofoleine, and javaphylline) of *M*. *speciosa* (S3 Table).

### Differential expression and abundance of alkaloids found in young and mature leaves of *M*. *speciosa*

Since the pharmacological actions of *M*. *speciosa* are associated with alkaloids, their expression in young and mature leaves was further assessed. Of the 63 putatively identified alkaloids, 38 are significantly (FDR < 0.05) different (S3 Table). Fold change (FC) analysis revealed 22 significantly different (|Log_2_FC| > 2) alkaloids between young and mature leaves, with 14 and 8 alkaloids exhibiting at least 4-fold higher expressions in young and mature leaves (Table 1).

**Table 1:** Identification of important features by statistical analysis of fold change upon comparing alkaloid expression between young and mature leaves.

No.	RT:m/z	Metabolite	Alkaloid subclass	Log_2_(FC)
1	8.53min:381.1808m/z	Vomicine	Indole	-8.0
2	5.34min:365.1858m/z	11-Methoxy-vinorine	Indole	-7.3
3	8.38min:351.171m/z	Perakine/ vomilenine/ polyneuridine aldehyde/ 19-epi-cathenamine/ cathenamine	Indole	-6.6
4	5.39min:367.2031m/z	Hirsuteine	Indole	-6.0
5	8.53min:349.1558m/z	Alstonine	Indole	-4.5
6	5.18min:243.0857m/z	Lumichrome	Pyrazine	3.8
7	5.21min:385.2113m/z	Rynchophylline	Indole	-3.4
8	5.31min:383.196m/z	Akuammine/ aricine/ cabucine/ lochnerinine	Indole	-3.4
9	4.90min:531.2313m/z	3-α(S)-Strictosidine	Indole	-3.1
10	5.55min:395.196m/z	Brucine	Indole	-3.1
11	4.91min:413.205m/z	(-)-Alstolucine A	Indole	3.1
12	5.18min:399.229m/z	Speciogynine	Indole	3.0
13	1.94min:130.087m/z	L-Pipecolic acid/ D-pipecolic acid	Piperidine	-2.9
14	4.22min:161.1076m/z	Tryptamine	Indole	-2.8
15	1.78min:181.071m/z	Theophylline/ theobromine/ paraxanthine	Purine	2.8
16	1.84min:322.147m/z	Acronycine/ 2-[4-(3,4-Methylenedioxyphenyl)butyl]-4(1H)-quinolinone	Unclassified	2.8
17	4.53min:372.1430m/z	(+)-N-(methoxycarbonyl)-N-norboldine	Isoquinoline	2.8
18	5.35min:353.185m/z	Ajmalicine	Indole	-2.7
19	2.10min:261.084m/z	1,2,3,4-Tetrahydro-β-carboline-1,3-dicarboxylic acid	Harmala	2.7
20	5.31min:369.2198m/z	Corynantheidine	Indole	-2.6
21	4.57min:369.180m/z	Mitraphylline/ isomitraphylline/ strictosidine aglycone/ horhammericine/ dialdehyde	Indole	2.5
22	4.87min:353.1839m/z	Akuammidine	Indole	-2.3

Alkaloids are deemed significant with a |Log_2_FC| > 2.

**Fig 5:**
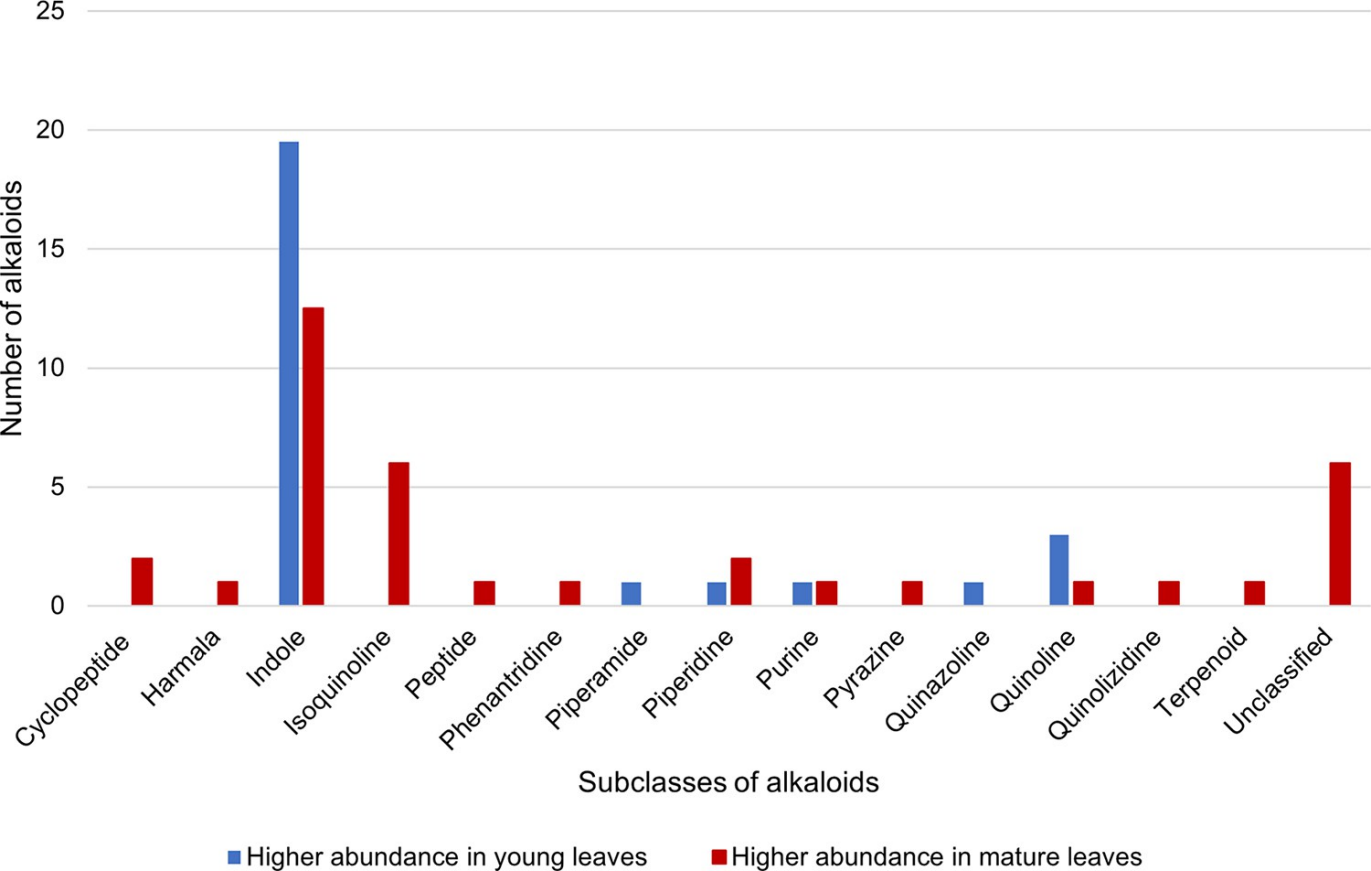
Bar chart representing the distribution of 63 annotated alkaloid features categorised to their respective subclasses. Alkaloid subclasses with a higher abundance in young leaves are coloured blue, while subclasses with a higher abundance in mature leaves are coloured red.

The relative abundance of alkaloids quantified in *M*. *speciosa* is tabulated according to their respective subclasses to compare their expression in young and mature leaves (S3 Table). According to the heatmap, most annotated alkaloids of various subclasses are more abundant in mature leaves than in young leaves (Fig 4). Many alkaloids of *M*. *speciosa* belonging to the isoquinoline, cyclopeptide, harmala, piperidine, terpenoid, peptide, phenanthridine, pyrazine, and quinolizidine subclasses are more abundant in the mature leaves, whereas alkaloids from the indole, quinoline, piperamide, and quinazoline subclasses are more abundant in the young leaves. Meanwhile, two purine alkaloids showed similar abundance in young and mature leaves (S3 Table and Fig 5).

**Fig 6:**
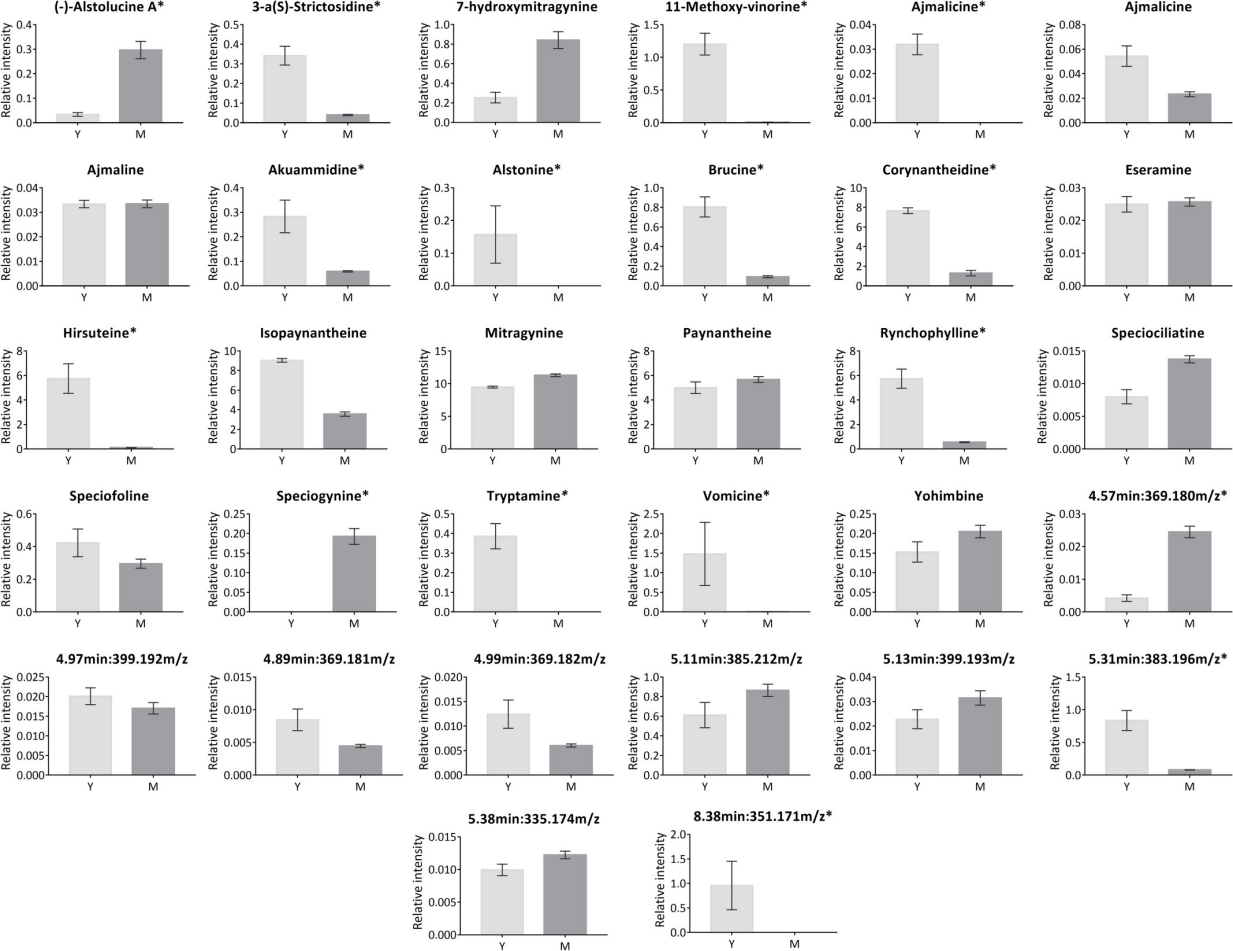
Relative abundance of 32 indole alkaloids putatively identified in young and mature leaves of *M*. *speciosa*. Relative intensity values for all indole alkaloids are provided in the S3 Table. Asterisks (*) indicate highly differential metabolites (* FDR < 0.05, |Log_2_FC| > 2). The graphs are arranged in alphabetical order. RT:m/z values are provided instead of compound names for metabolite features annotated to more than one compound, which can be referred to in the S3 Table.

Since indoles are the most prominent category of alkaloids in *M*. *speciosa*, further comparisons were focused on these compounds (Fig 6). Among the 32 annotated indole alkaloids, 13 are significantly more abundant in young leaves, while 3 are more abundant in mature leaves (S3 Table and Fig 6). Interestingly, two of the most studied indole alkaloids of *M*. *speciosa*, MG and its derivative, 7-OHMG, showed a higher expression in mature leaves than young leaves. Relative quantification analysis showed that MG is 1.2-fold higher in mature leaves than young leaves, while 7-OHMG is 3.3-fold higher in mature leaves (S3 Table and Fig 6).

## Discussion

### Untargeted metabolite profiling of *M*. *speciosa* leaves using LC-ESI-TOF-MS

Untargeted metabolomics provides comprehensive and unbiased qualitative and quantitative analyses of each metabolite in an organism [77]. LC-MS is a typical approach for analysing a wide spectrum of plant metabolites in untargeted metabolomics [62,78–81]. Additionally, the combination of LC and ESI-TOF-MS in this investigation improves mass accuracy, allowing for faster metabolite identification and quantification [62,81,82]. Recent untargeted metabolomics followed by a targeted quantification study of *M*. *speciosa* profiled 53 commercial kratom products. Compound identification was only focused on three targeted alkaloids, hampering the identification of other potentially bioactive compounds [48].

In this study, we focused on untargeted metabolite profiling to explore the secondary metabolite composition in the young and mature leaves of *M*. *speciosa*. Metabolite annotation was manually conducted using mass-based (m/z values) searches against several online databases and putatively identified 86 metabolites, of which 2 were identified using authentic standards (ID level 1), 39 were successfully identified using MS/MS data (ID level 2), and 45 annotated using MS data (ID level 3). In addition to alkaloids, 23 other secondary metabolites are categorised into flavonoids, terpenoids, and phenylpropanoids, while the rest comprises a carboxylic acid, a glucoside, a phenol, and a phenolic aldehyde (S3 and S4 Tables). Previously, León et al. [41] isolated and identified 10 other phytochemicals on top of 8 known *M*. *speciosa* alkaloids. The 10 phytochemicals isolated comprise a flavonoid, a saponin, two triterpenoid saponins, two monoaryl glycosides, and two cyclohexanone glycosides [41]. Several years later, Charoonratana et al. [83] determined the metabolite profiles in *M*. *speciosa* via NMR and HPLC analyses to identify the metabolites involved in the biosynthesis of MG. Besides MG, 15 metabolites were identified, including flavonoids, iridoids, triterpenes, organic acids, phenolic acids, amino acids, and sugar [83]. In our study, only two previously reported compounds, epicatechin (flavonoid) (ID level 2) and secologanin (terpenoid) (ID level 3), were annotated. The rest of the 21 secondary metabolites are reported for the first time in *M*. *speciosa* (20 metabolites putatively identified with ID level 2 and 1 metabolite annotated with ID level 3) (S4 Table). These metabolite annotations are only putative due to inadequate data on *M*. *speciosa* metabolites in public databases. Due to the various acquisition of metabolic data with different analytical instruments and methods, a complete spectral database for LC-MS is nearly unfeasible [62,70,84,85]. Nonetheless, a comprehensive metabolite profiling reported in the present study grants a future search of more potential metabolites in the plant.

### Variation of secondary metabolites between young and mature leaves

Various findings have shown that the composition of secondary metabolites differs in young and mature leaves [52,54,55,77,86]. Recent studies have found that *Gingko biloba* L. [52] and sugarcane [55] contain higher secondary metabolites in older leaves than in immature leaves. However, the younger leaves of *Melicope ptelefolia* [77] and *Inga* trees [87] had higher concentrations of secondary metabolites than mature leaves. Thus, secondary metabolite expressions in young and mature leaves differ between species. Previous research on *M*. *speciosa* mostly involved targeted isolations and structural elucidation of alkaloids. Initial investigations on the young and mature leaves of *M*. *speciosa* collected from different geographical regions isolated and revealed only four alkaloids with a higher abundance in young leaves than mature leaves using TLC examination [43]. A recent study on Malaysian *M*. *speciosa* leaves published targeted isolations of 10 indole and oxindole alkaloids and profiled the alkaloids of leaves gathered from various locations in the northern states [7]. However, the study of expression levels of many other secondary metabolites, including alkaloids, in young and mature leaves is lacking.

In this study, various secondary metabolites in the young and mature leaves of *M*. *speciosa* were comprehensively compared using untargeted metabolomics. Overall, the multivariate analysis showed that leaf age is a vital separating factor that led to clear discrimination among young and mature leaves collected from the same tree at the same time point (Fig 3). Our study also found that mature *M*. *speciosa* leaves expressed higher levels of secondary metabolites than young leaves (Fig 4), further indicating that metabolites are distributed differently in the young and mature leaves of the same tree. Furthermore, many alkaloids of *M*. *speciosa* belonging to various subclasses are more abundant in mature leaves than young leaves (Fig 5), implying that the concentrations of these metabolites increased with age. Most alkaloids are toxic and are typically distributed in sections of plants most threatened by the attacks of herbivores, insects, and/or microorganisms [88–90]. Generally, susceptible organs and tissues like seeds, plantlets, buds, and young leaves require more protection than aged ones; hence, more defence substances are synthesised or sequestered [90,91]. However, the distribution of alkaloids as defensive substances among young and mature leaves follows different trends in herbs and trees. A “phenological defence” is given to simultaneously occurring flushes of new leaves in trees, so there is more toxin accumulation in mature leaves because of the protection needed by mature leaves until fresh shoots of young leaves are formed [90,92]. Some metabolites are only found in either young or mature leaves; the diversity found among these specific metabolites in leaves of different maturity presumably reflects the evolution of metabolites towards various roles during leaf growth.

On the other hand, apart from a few key indole alkaloids in *M*. *speciosa* like MG and its derivative 7-OHMG that showed higher abundance in mature leaves, most of the putatively identified indole alkaloids in *M*. *speciosa*, such as corynantheidine, hirsuteine, rynchophylline, vomicine, tryptamine, and 3-α(S)-strictosidine among others, are significantly abundant in young leaves than mature leaves (Fig 6), implying that these indole alkaloids are highly synthesised in young leaves. Similar findings were observed on several indole alkaloid-producing plants, i.e., *Camptotheca acuminata*, *Catharanthus roseus*, *Rauvolfia serpentina*, and *Uncaria tomentosa* [93–97]. A previous study suggested that indole alkaloids found to be significantly abundant in young *M*. *speciosa* leaves may be the precursors of mitragynine and its derivatives [43]. Indole and oxindole alkaloids are usually produced via complex and divergent enzymatic steps of the monoterpenoid indole alkaloid (MIA) biosynthesis pathway [7]. The MIA biosynthesis is generally initiated with the condensation of the key precursor, 3-α(S)-strictosidine, from tryptamine (indole precursor) and secologanin (terpenoid precursor), catalysed by *strictosidine synthase* (STR) [98,99]. Two of the important MIA biosynthesis precursors, tryptamine and 3-α(S)-strictosidine, annotated in this study, are significantly greater in young leaves (Fig 6), further supporting the hypothesis of Houghton et al. [43]. Therefore, this study also identifies possible intermediates of the missing steps in the MIA pathway of *M*. *speciosa*. In short, our metabolomics analysis has provided insights into the different compositions of secondary metabolites in the young and mature leaves of *M*. *speciosa*.

It is also important to mention that the major obstacle faced during the identification of *M*. *speciosa* alkaloids was due to the chemical similarity of the indole and oxindole alkaloids and their small differences in molecular weights [100]. Often, MS/MS data are insufficient to distinguish structural and stereoisomers [70]. Hence, further validation of the potential indole and oxindole alkaloids using authentic standards or NMR is needed to support the current observation.

### New putatively identified secondary metabolites show broader therapeutic potentials of *M*. *speciosa*

Despite the wide consumption of *M*. *speciosa* in Southeast Asian countries for health and well-being, studies to assess the complete spectrum of biological activities in *M*. *speciosa* are still lacking. Several studies deduced that the methanol extracts of *M*. *speciosa* contain a mixture of compounds that possibly share similar pharmacological activities, e.g., muscle relaxation [101] and antidiabetic activity [102], to be more effective or potent than a single compound like MG. Through our untargeted metabolomics analysis, several unreported alkaloids that are probable contributors to the known and unknown medicinal properties of *M*. *speciosa* are shortlisted (Table 2). Fold change analysis identified three alkaloids (vomicine, hirsuteine, and alstonine) among the top ten most significant alkaloids. Vomicine isolated from the seeds of *Strychnos nux-vomica* exhibited anti-diabetic activity in albino rats [103]. Hirsuteine isolated from *Uncaria sinensis* showed neuroprotective activity in rats via Ca^2+^ influx suppression [104]. Likewise, alstonine, commonly found in the Apocynaceae plant family, showed antimutagenic properties in mice bearing YC8 lymphoma cells and Ehrlich ascitic carcinoma cells [105]. Besides, alstonine also showed a dose-dependent and potent antipsychotic profile in mice models [106]. Furthermore, other secondary metabolites like flavonoids and terpenoids also contribute to antioxidant properties in plants [55,78]. Therefore, the collective effects of all the newly annotated alkaloids of *M*. *speciosa* may be responsible for several aforementioned biological activities reported in *M*. *speciosa* leaf extracts [14,15,20–27].

**Table 2:** Newly identified alkaloids of *M*. *speciosa* and their biological significance.

Compounds	Molecular Formula	Biological activity	Reference
**Indole alkaloids**			
*Akuammidine	C_21_H_24_N_2_O_3_	Anti-asthmatic Anti-inflammatory Analgesic Antitussive Antidepressant	[107] [108] [109] [110]
*Alstonine	C_21_H_20_N_2_O_3_	Antipsychotic Anticancer	[106,111] [105]
*Hirsuteine	C_22_H_26_N_2_O_3_	Neuroprotective	[104]
*Tryptamine	‎C_10_H_12_N_2_	Neurotransmitter & neuromodulator Vasoconstrictor & vasodilator Antimicrobial & antibacterial Antioxidant & antifungal agents	[112]
*Vomicine	C_22_H_24_N_2_O_4_	Antidiabetic	[103]
Yohimbine	C_21_H_26_N_2_O_3_	Aids weight loss Aphrodisiac (love drug) Mydriatics (induces dilation of the pupil) Antidiabetic Antifungal	[113] [114] [115]
**Pyrroloindole alkaloid**			
Eseramine	C_16_H_22_N_4_O_3_	Anticholinesterase	[116]
**Isoquinoline alkaloids**			
*(+)-N-(methoxycarbonyl)-N-norboldine	C_20_H_21_NO_6_	Antimicrobial agent	[117]
**Quinazoline alkaloids**			
Vasicinol	C_11_H_12_N_2_O_2_	Anticholinesterase	[118]
**Terpenoid alkaloids**			
Daphniphylline	C_32_H_49_NO_5_	Central nervous system depressant	[119]
**Piperidine alkaloids**			
Prosopinine	C_18_H_35_NO_3_	Sedative	[120]
**Purine alkaloids**			
Caffeine	C_8_H_10_N_4_O_2_	Stimulant	[121]

Asterisks (*) indicate highly differential metabolites (FDR < 0.05, |Log_2_FC| > 2).

In this study, although the young and mature leaves from the same *M*. *speciosa* tree shared many similar metabolites (Fig 2), individual metabolites vary in abundance (S3 and S4 Tables; Figs 4 and 6), suggesting that the metabolomics analysis of different leaf organs aids in determining the part with the most potent medicinal effects. Most of the annotated compounds with notable biological activities like vomicine, hirsuteine, alstonine, akuammidine, and tryptamine are indole alkaloids, which showed a significant abundance in the young leaves (Table 1 and Fig 6), warranting further studies.

## Conclusions

This study reports a comprehensive metabolome from young and mature leaves of *M*. *speciosa* via LC-ESI-TOF-MS. In total, 86 metabolites were annotated as alkaloids, flavonoids, terpenoids, phenylpropanoids, carboxylic acid, glucoside, phenol, and phenolic aldehyde. Diverse alkaloid profiles were also identified and classified into 14 subclasses, with 13 subclasses of alkaloids that have not been reported in *M*. *speciosa*. These alkaloids are potentially associated with the physiological and biochemical progressions during leaf maturity and plant defence against herbivores, insects, and pathogens. Our findings support *M*. *speciosa* as a prominent source of biologically active alkaloids, which can be potentially used for myriads of therapeutic uses, including managing depression, cancer, and diabetes. Interestingly, this study also found that young leaves contain more significantly abundant and medicinally beneficial alkaloids, all of which have prominent bioactivities. Altogether, this work adds to our understanding of metabolite composition in the young and mature leaves of *M*. *speciosa*, paving the way for future identification of new biologically active compounds.

## Supporting information

S1 Fig The young (A) and mature (B) leaves of *M*. *speciosa*.
(JPG)

S2 Fig Chromatograms of mitragynine (MG) and 7-hydroxymitragynine (7-OHMG) authentic standards identified using LC-MS. (JPG)

S3 Fig Principal component analysis (PCA) of young (Y) and mature (M) leaves of *M*. *speciosa* with technical replicates. (TIF)

S1 Table List of alkaloids identified in *M*. *speciosa* from previous studies based on first occurrences in this species. (DOCX)

S2 Table Parameter acquisition for *M*. *speciosa* metabolites identified via multiple reaction monitoring (MRM). (DOCX)

S3 Table Identification of alkaloids in the young (Y) and mature (M) leaves of *M*. *speciosa*. (DOCX)

S4 Table Putative identification of other secondary metabolites in the young (Y) and mature (M) leaves of *M*. *speciosa*. (DOCX)

S1 Text Authentication of plant material. (DOCX)

S1 Appendix LC-ESI-TOF-MS spectra of metabolite features putatively identified at ID level 3 (identification using m/z value only due to absence of fragments). (DOCX)

S2 Appendix LC-ESI-TOF-MS/MS spectra of metabolite features putatively identified at ID level 2 (identification at fragmentation level [MS2] by matching with online databases). (DOCX)

S1 Raw data (XLSX)

S1 Data (XLSX)
