# Supplementary figures and images for: Comparative metabolomics analysis reveals alkaloid repertoires in young and mature Mitragyna speciosa (Korth.) Havil. Leaves
Source: PLoS One. 2023 Mar 21;18(3):e0283147. doi: 10.1371/journal.pone.0283147 (PMC10030037; doi:10.1371/journal.pone.0283147)

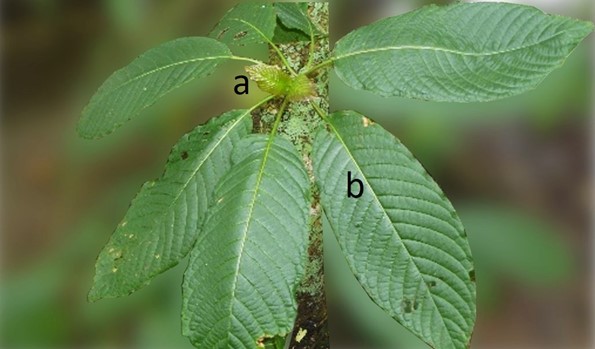

Supplement: S1 Fig — The young (A) and mature (B) leaves of M. speciosa. (JPG) [file pone.0283147.s001.jpg]

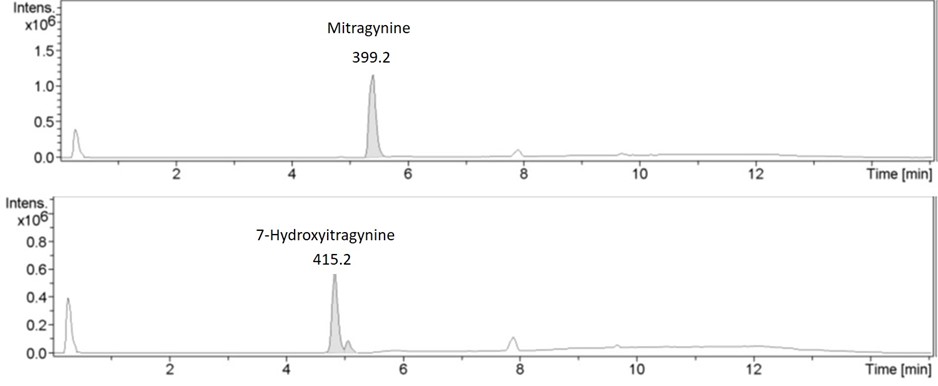

Supplement: S2 Fig — (JPG) [file pone.0283147.s002.jpg]

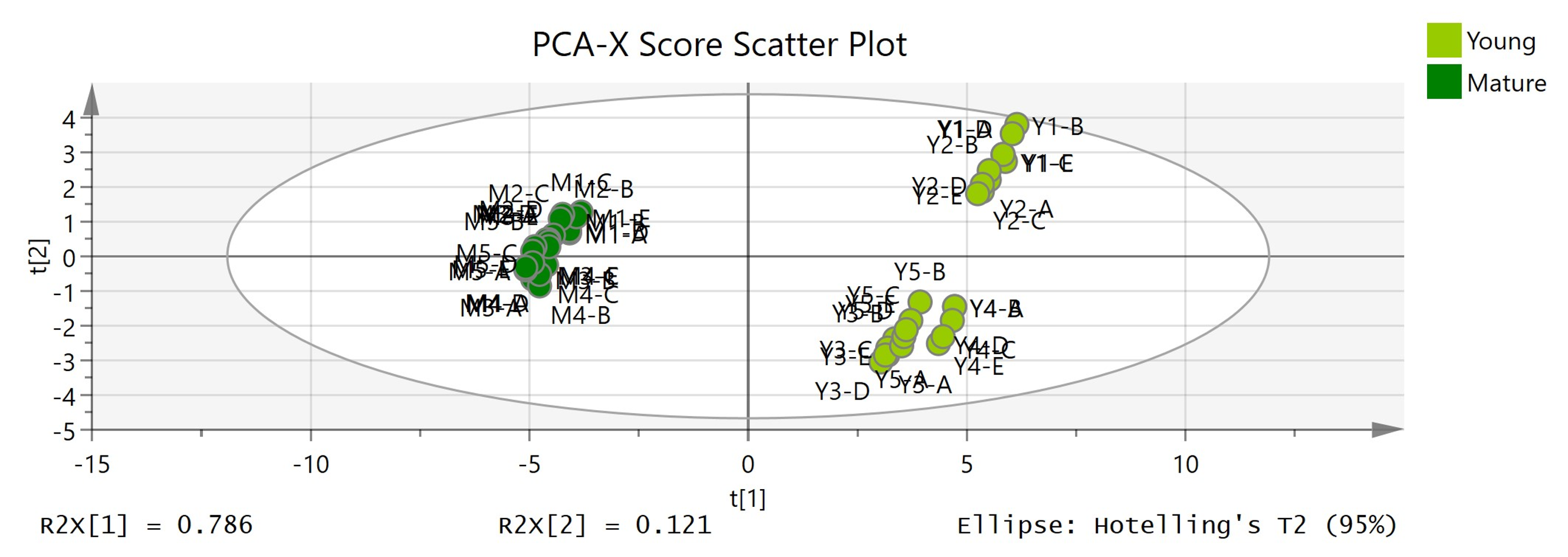

Supplement: S3 Fig — (TIF) [file pone.0283147.s003.tif]
