# Supplementary material for: Comparative metabolomics analysis reveals alkaloid repertoires in young and mature Mitragyna speciosa (Korth.) Havil. Leaves
Source: PLoS One. 2023 Mar 21;18(3):e0283147. doi: 10.1371/journal.pone.0283147 (PMC10030037; doi:10.1371/journal.pone.0283147)
Supplement: S1 Table — (DOCX) [file pone.0283147.s004.docx]

**S1 Table** List of alkaloids identified in M. speciosa from previous studies based on first occurrence in this species.

| **No.** | **Alkaloids** | **Plant material** | **Location** | **Reference** |
| --- | --- | --- | --- | --- |
| 1 | Mitragynine* | Leaves | Malaya | [1] |
| 2 | Mitraspecine | Bark  Wood | - | [2] |
| 3 | Stipulatine (Rotundifoline) | Bark  Leaves | Philippines | [3] |
| 4 | Rhynchophylline* | Bark  Leaves | Philippines | [3] |
| 5 | Speciofoline* | Leaves | Malaya | [4] |
| 6 | Ajmalicine* | Leaves | Thailand | [5] |
| 7 | Corynantheidine* | Leaves | Thailand | [5] |
| 8 | Speciogynine | Leaves | Thailand | [5] |
| 9 | Paynantheine | Leaves | Thailand | [5] |
| 10 | Speciociliatine | Leaves | Thailand | [5] |
| 11 | Speciophylline | Leaves | Thailand | [6] |
| 12 | Mitraphylline | Leaves | Thailand | [6] |
| 13 | Isomitraphylline | Leaves | Thailand | [6] |
| 14 | Specionoxeine | Leaves | New Guinea | [7] |
| 15 | Isospecionoxeine | Leaves | New Guinea | [7] |
| 16 | Ciliaphylline | Leaves | New Guinea | [7] |
| 17 | Isorotundifoline | Leaves | New Guinea | [7] |
| 18 | Isospeciofoline* | Leaves | Thailand | [8] |
| 19 | Isomitrafoline | Leaves | Thailand | [8] |
| 20 | Mitrafoline | Leaves | Thailand | [8] |
| 21 | Rotundifoleine | Leaves | Thailand | [8] |
| 22 | Isorotundifoleine | Leaves | Thailand | [8] |
| 23 | Corynoxine A | Leaves (mature tree) | Thailand | [9] |
| 24 | Corynoxine B | Leaves (mature tree) | Thailand | [9] |
| 25 | Corynoxeine | Leaves (mature tree) | Thailand | [9] |
| 26 | Isocorynoxeine | Leaves (mature tree) | Thailand | [9] |
| 27 | Isorhynchophylline | Leaves (mature tree)  Leaves  Stem bark  Root bark  (Young plant) | Thailand | [9,10] |
| 28 | Isocorynantheidine | Leaves  (Young plant) | Thailand | [10] |
| 29 | Isopaynantheine | Leaves  (Young plant) | Thailand | [10] |
| 30 | Mitraciliatine | Leaves  (Young plant) | Thailand | [10] |
| 31 | Rhynchophylline | Leaves  Young twigs  Stem bark  (Mature tree)  Stem bark  Root bark  (Young plant) | Thailand | [9,10] |
| 32 | Mitragynine oxindole A | Young twigs  Stem bark  (Mature tree Stem bark  Young plant) | Thailand | [9,10] |
| 33 | Mitragynine oxindole B | Young twigs  Stem bark  (Mature tree Stem bark  Young plant) | Thailand | [9,10] |
| 34 | Mitrajavine | Leaves  Root bark  (Young plant) | Thailand | [10] |
| 35 | Javaphylline | Leaves  Stem bark  (Young plant) | Thailand | [10] |
| 36 | Akuammigine | Leaves  (Young plant) | Thailand | [10] |
| 37 | 3-isoajmalicine | Leaves  (Young plant) | Thailand | [10] |
| 38 | 3-dehydromitragynine | Leaves | Selangor, Malaysia | [11] |
| 39 | Mitragynalinic acid | Young leaves  Mature leaves | Selangor, Malaysia  Perlis, Malaysia | [12] |
| 40 | Corynantheidinalinic acid | Young leaves  Mature leaves | Selangor, Malaysia  Perlis, Malaysia | [12] |
| 41 | Mitragynaline* | Young leaves  Mature leaves | Selangor, Malaysia  Perlis, Malaysia | [12] |
| 42 | Corynantheidaline | Young leaves  Mature leaves | Selangor, Malaysia  Perlis, Malaysia | [12] |
| 43 | 7α-hydroxy-7H-mitragynine* | Leaves | Thailand | [13] |
| 44 | 3,4,5,6-tetradehydromitragynine | Mature leaves | Malaysia | [14] |
| 45 | Mitralactonal | Mature leaves | Malaysia | [14] |
| 46 | Mitrasulgynine | Mature leaves | Malaysia | [14] |
| 47 | Mitralactonine | Young leaves | Malaysia | [15] |
| 48 | 9-methoxymitralactonine | Young leaves | Malaysia | [16] |
| 49 | 7-hydroxyspeciociliatine | Fruits | Malaysia | [17] |
| 50 | 7β-hydroxy-7H-mitraciliatine | Leaves | - | [18] |
| 51 | Isospeciofoleine | Leaves | - | [18] |
| 52 | Mitragynine-N(4)-oxide | Green Maeng Da (chipped leaf) | Southeast Asia | [19,20] |
| 53 | Speciociliatine-N(4)-oxide | Green Maeng Da (chipped leaf) | Southeast Asia | [19,20] |
| 54 | Isopaynantheine-N(4)-oxide | Green Maeng Da (chipped leaf) | Southeast Asia | [20] |
| 55 | Epiallo-isopaynantheine | Green Maeng Da (chipped leaf) | Southeast Asia | [20] |
| 56 | Epiallo-isopaynantheine-N(4)-oxide | Green Maeng Da (chipped leaf) | Southeast Asia | [20] |
| 57 | 3-epirhynchophylline | White Jongkong (fine powder) | Southeast Asia | [20] |
| 58 | 3-epicorynoxine B | White Jongkong (fine powder) | Southeast Asia | [20] |

*Indole and oxindole alkaloids (mitragynine, 7-hydroxymitragynine, mitraphylline, isomitraphylline, javaphylline, speciogynine, isorynchophylline, corynoxine, corynoxine B, speciofoline, isospeciofoleine, rynchophylline, ajmalicine, and corynantheidine, paynantheine, isopaynantheine, speciociliatine) that were also annotated in this study.

**References**

1. Field E. Mitragynine and mitraversine, two new alkaloids from species of *Mitragyne*. J Chem Soc Trans. 1921;119: 887–891.

2. Denis P. Mitraspecine, a new alkaloid from *Mitragyna speciosa*, Korthals. Bull la Cl des Sci. 1938;24: 653–658.

3. Hendrickson JB, Sims JJ. *Mitragyna* alkaloids: the structure of stipulatine. Tetrahedron Lett. 1963;4: 929–935.

4. Beckett AH, Lee CM, Tackie AN. The structure of speciofoline and ‘stipulatine’ (rotundifoline). Tetrahedron Lett. 1963;25: 1709–1714.

5. Beckett AH, Shellard EJ, Phillipson JD, Lee CM. The *Mitragyna* species of Asia. Part VII. Indole alkaloids from the leaves of *Mitragyna speciosa* Korth. Planta Med. 1966;14: 277–288.

6. Beckett AH, Shellard EJ, Phillipson JD, Lee CM. The *Mitragyna* species of Asia. VI. Oxindole alkaloids from the leaves of *Mitragyna speciosa* Korth. Planta Med. 1966;14: 266–276.

7. Trager WF, Lee CM, Phillipson JD, Haddock RE, Dwuma-Badu D, Beckett AH. Configurational analysis of rhynchophylline-type oxindole alkaloids : the absolute configuration of ciliaphylline, rhynchociline, specionoxeine, isospecionoxeine, rotundifoline and isorotundifoline. Tetrahedron. 1968;24: 523–543. doi:10.1016/0040-4020(68)88002-0

8. Hemingway SR, Houghton PJ, Phillipson JD, Shellard EJ. 9-Hydroxyrhynchophylline-type oxindole alkaloids. Phytochemistry. 1975;14: 557–563. doi:10.1016/0031-9422(75)85128-4

9. Shellard EJ, Houghton PJ, Resha M. The *Mitragyna* species of Asia. Part XXXI. The alkaloids of *Mitragyna speciosa* Korth from Thailand. Planta Med. 1978;34: 26–36.

10. Shellard EJ, Houghton PJ, Resha M. The *Mitragyna* species of Asia. Part XXXII. The distribution of alkaloids in young plants of *Mitragyna speciosa* Korth grown from seed obtained from Thailand. Planta Med. 1978;34: 253–263.

11. Houghton PJ, Said IM. 3-dehydromitragynine: An alkaloid from *Mitragyna speciosa*. Phytochemistry. 1986;25: 2910–2912. doi:10.1016/S0031-9422(00)83771-1

12. Houghton PJ, Latiff A, Said IM. Alkaloids from *Mitragyna speciosa*. Phytochemistry. 1991;30: 347–350.

13. Ponglux D, Wongseripipatana, S., Takayama H, Kikuchi M, Kurihara M, M. K, Aimi N, et al. A new indole alkaloid, 7 alpha-hydroxy-7H-mitragynine, from *Mitragyna speciosa* in Thailand. Planta Med. 1994;60: 580–581.

14. Takayama H, Kurihara M, Kitajima M, Said IM, Aimi N. New indole alkaloids from the leaves of malaysian: *Mitragyna speciosa*. Tetrahedron. 1998;54: 8433–8440. doi:10.1016/S0040-4020(98)00464-5

15. Takayama H, Kurihara M, Kitajima M, Said IM, Aimi N. Isolation and asymmetric total synthesis of a new *Mitragyna* indole alkaloid, (–)-mitralactonine. J Org Chem. 1999;64: 1772–1773.

16. Takayama H, Kurihara M, Kitajima M, Said IM, Aimi N. Structure elucidation and chiral-total synthesis of a new indole alkaloid, (-)-9-methoxymitralactonine, isolated from *Mitragyna speciosa* in Malaysia. Tetrahedron. 2000;56: 3145–3151. doi:10.1016/S0040-4020(00)00235-0

17. Kitajima M, Misawa K, Kogure N, Said IM, Horie S, Hatori Y, et al. A new indole alkaloid, 7-hydroxyspeciociliatine, from the fruits of Malaysian *Mitragyna speciosa* and its opioid agonistic activity. J Nat Med. 2006;60: 28–35. doi:10.1007/s11418-005-0001-7

18. Ali Z, Demiray H, Khan IA. Isolation, characterization, and NMR spectroscopic data of indole and oxindole alkaloids from *Mitragyna speciosa*. Tetrahedron Lett. 2014;55: 369–372. doi:10.1016/j.tetlet.2013.11.031

19. Shellard EJ, Houghton PJ, Resha M. The *Mitragyna* species of Asia. Part XXX: Oxidation products of mitragynine and speciociliatine. Planta Med. 1978;34: 253–263. doi:10.1055/s-0028-1097448

20. Flores-bocanegra L, Raja HA, Graf TN, Augustinovic M, Wallace ED, Hematian S, et al. The chemistry of kratom [*Mitragyna speciosa*]: updated characterization data and methods to elucidate indole and oxindole alkaloids. J Nat Prod. 2020;83: 2165–2177. doi:10.1021/acs.jnatprod.0c00257
