# Supplementary material for: Comparative metabolomics analysis reveals alkaloid repertoires in young and mature Mitragyna speciosa (Korth.) Havil. Leaves
Source: PLoS One. 2023 Mar 21;18(3):e0283147. doi: 10.1371/journal.pone.0283147 (PMC10030037; doi:10.1371/journal.pone.0283147)
Supplement: S2 Table — (DOCX) [file pone.0283147.s005.docx]

**S2 Table** Parameter acquisition for M. speciosa metabolites identified via multiple reaction monitoring (MRM).

| **No.** | **Metabolite** | **Ion transition (m/z)**  **(Precursor>Product ion)** | **Collision energy (eV)** |
| --- | --- | --- | --- |
| 1 | Vasicinol | 205.09>146.05 | 20.2 |
| 2 | Strictosidine | 531.23>514.20  531.23>352.15  531.23>340.15  531.23>283.11  531.23>282.11 | 35.9 |
| 3 | Hirsutine | 369.21>144.08  369.21>170.09  369.21>226.14  369.21>238.14  369.21>337.19 | 28.5 |
| 4 | 11-Methoxy-vinorine | 365.18>197.10  365.18>249.13  365.18>298.14  365.18>279.11 | 28.3 |
| 5 | Hirsuteine | 367.19>251.15  367.19>197.10  367.19>226.14  367.19>144.08  367.19>238.14 | 28.4 |
| 6 | Brucine | 395.19>227.11  395.19>281.16  395.19>309.12  395.19>381.17  395.19>382.18 | 29.9 |
| 7 | Rutin | 611.16>127.03  611.16>303.04  611.16>305.05 | 38.3 |
| 8 | Sinapate | 225.07>147.04  225.07>151.03  225.07>165.05  225.07>175.03 | 21.2 |
| 9 | Glabric acid | 487.34>469.33  487.34>135.11  487.34>470.33  487.34>163.14  487.34>191.18 | 34.4 |
| 10 | Magnoshinin | 415.21>119.08 | 30.8 |
