# Supplementary material for: Comparative metabolomics analysis reveals alkaloid repertoires in young and mature Mitragyna speciosa (Korth.) Havil. Leaves
Source: PLoS One. 2023 Mar 21;18(3):e0283147. doi: 10.1371/journal.pone.0283147 (PMC10030037; doi:10.1371/journal.pone.0283147)
Supplement: S3 Table — (DOCX) [file pone.0283147.s006.docx]

**S3 Table. Putative identification of alkaloids in the young (Y) and mature (M) leaves of *M. speciosa***

| **Peak**  **no.^a^ (N)** | **RT, min** | **Parent ion**  **(*m/z*)**  **[M+H]^+^** | **Molecular formula** | **Mass error**  **(ppm)** | **Fragment ions**  **[M+H]^+^** | **Fragment**  **formula** | **Metabolite ID** | **ID level^b^** | **Reference** | **VIP** | **FC** | **Log_2_FC^c^** | **P-value** | **FDR^c^** | **Rel. abundance^d^**  **(Mean ± SEM)** | |
| --- | --- | --- | --- | --- | --- | --- | --- | --- | --- | --- | --- | --- | --- | --- | --- | --- |
|  |  |  |  |  |  |  |  |  |  |  |  |  |  |  | **Y** | **M** |
| **Indole alkaloids** | | |  |  |  |  |  |  |  |  |  |  |  |  |  |  |
| 11 | 4.22 | 161.1076 | C_10_H_12_N_2_ | 1.7 | 144.0814  117.0720 | [C_10_H_10_N]^+^  - | **Tryptamine | 2 | KEGG | 1.30 | 0.1 | -2.8 | 6.1E-06 | 2.6E-05 | 0.386 ± 0.065 | n.d |
| 13 | 4.57 | 369.180 | C_21_H_24_N_2_O_4_ | -2.3 | - | - | ^∆^**Mitraphylline,  Isomitraphylline,  Strictosidine aglycone,  Horhammericine,  Dialdehyde | 3 | KNApSAcK  KEGG | 1.24 | 5.8 | 2.5 | 1.9E-04 | 5.5E-04 | 0.004 ± 0.001 | 0.024 ± 0.002 |
| 17 | 4.75 | 355.1996 | C_21_H_26_N_2_O_3_ | -5.7 | 144.0816  212.1269  224.1293  337.1910 | [C_10_H_9_N]^+^H^+^  [C_11_H_17_NO_3_]^+^H^+^  [C_15_H_16_N_2_]^+^  [C_21_H_25_N_2_O_2_]^+^ | Yohimbine | 2 | KEGG | 0.43 | 1.3 | 0.4 | 1.3E-01 | 1.6E-01 | 0.153 ± 0.026 | 0.205 ± 0.016 |
| 18 | 4.77 | 327.201 | C_20_H_26_N_2_O_2_ | -17.4 | - | - | Ajmaline | 3 | KEGG | 0.11 | 1.0 | 0.0 | 9.8E-01 | 9.8E-01 | 0.033 ± 0.001 | 0.033 ± 0.002 |
| 20 | 4.87 | 353.1839 | C_21_H_24_N_2_O_3_ | -5.9 | 144.0794  212.1273  335.1731  224.1265  323.1768 | [C_10_H_8_N+H]^+^H^+^  [C_11_H_16_NO_3_+H]^+^H^+^  [C_21_H_23_N_2_O_2_]^+^  [C_12_H_17_NO_3_]^+^H^+^  [C_20_H_21_N_2_O_2_+H]^+^H^+^ | **Akuammidine | 2 | KEGG | 1.07 | 0.2 | -2.3 | 1.4E-03 | 3.0E-03 | 0.283 ± 0.067 | 0.059 ± 0.003 |
| 21 | 4.89 | 369.181 | C_21_H_24_N_2_O_4_ | 0.3 | - | - | ^∆^Mitraphylline,  Isomitraphylline,  Strictosidine aglycone,  Horhammericine,  Dialdehyde | 3 | KNApSAcK  KEGG | 0.60 | 0.5 | -0.9 | 4.6E-02 | 6.7E-02 | 0.008 ± 0.002 | 0.004 ± 0.000 |
| 22 | 4.90 | 531.2313 | C_27_H_34_N_2_O_9_ | -4.5 | 514.2049  352.1529  340.1539  282.1112  283.1189 | [C_27_H_33_NO_9_-H]^+^  [C_21_H_22_NO_4_]^+^  [C_20_H_20_NO_4_+H]^+^H^+^  [C_14_H_18_O_6_]^+^  [C_17_H_17_NO_3_]^+^ | **3-α(S)-Strictosidine | 2 | KEGG | 1.37 | 0.1 | -3.1 | 1.8E-06 | 8.8E-06 | 0.342 ± 0.048 | 0.032 ± 0.005 |
| 23 | 4.91 | 413.203 | C_23_H_28_N_2_O_5_ | -9.9 | - | - | **(-)-Alstolucine A | 3 | ChEBI | 1.38 | 8.8 | 3.1 | 2.3E-05 | 8.6E-05 | 0.034 ± 0.008 | 0.296 ± 0.036 |
| 25 | 4.97 | 399.192 | C_22_H_26_N_2_O_5_ | 2.2 | - | - | ^∆^Isospeciofoleine,  Javaphylline | 3 | PubChem  KNApSAcK | 0.28 | 0.8 | -0.2 | 2.8E-01 | 3.2E-01 | 0.020 ± 0.002 | 0.017 ± 0.001 |
| 26 | 4.99 | 369.182 | C_21_H_24_N_2_O_4_ | 3.0 | - | - | ^∆^Mitraphylline,  Isomitraphylline  Strictosidine aglycone,  Horhammericine,  Dialdehyde | 3 | KNApSAcK  KEGG | 0.60 | 0.5 | -1.0 | 7.7E-02 | 1.0E-01 | 0.012 ± 0.003 | 0.006 ± 0.000 |
| 27 | 5.02 | 319.172 | C_16_H_22_N_4_O_3_ | -14.0 | - | - | Eseramine | 3 | KEGG | 0.15 | 1.0 | 0.0 | 7.2E-01 | 7.3E-01 | 0.025 ± 0.002 | 0.026 ± 0.001 |
| 28 | 5.06 | 401.2074 | C_22_H_28_N_2_O_5_ | 0.8 | 238.1435  369.1812  226.1446  176.0695  239.1497 | [C_13_H_20_NO_3_]^+^  [C_21_H_25_N_2_O_4_]^+^  [C_12_H_18_NO_3_+H]^+^H^+^  [C_10_H_9_NO_2_]+H^+^  [C_13_H_21_NO_3_]^+^ | Speciofoline | 2 | PubChem | 0.40 | 0.7 | -0.5 | 3.0E-01 | 3.3E-01 | 0.423 ± 0.085 | 0.296 ± 0.028 |
| 29 | 5.11 | 385.212 | C_22_H_28_N_2_O_4_ | -0.5 | - | - | Isorynchophylline, Corynoxine, Corynoxine B | 3 | ChEBI | 0.50 | 1.4 | 0.5 | 1.3E-01 | 1.6E-01 | 0.613 ± 0.129 | 0.865 ± 0.063 |
| 30 | 5.13 | 399.193 | C_22_H_26_N_2_O_5_ | 4 | - | - | ^∆^Isospeciofoleine,  Javaphylline | 3 | PubChem  KNApSAcK | 0.46 | 1.4 | 0.5 | 1.0E-01 | 1.4E-01 | 0.023 ± 0.004 | 0.031 ± 0.003 |
| 34 | 5.18 | 399.229 | C_23_H_30_N_2_O_4_ | 2.9 | - | - | **Speciogynine | 3 | KNApSAcK | 1.36 | 8.2 | 3.0 | 6.5E-08 | 1.1E-06 | n.d | 0.193 ± 0.020 |
| 35 | 5.18 | 415.2220 | C_23_H_30_N_2_O_5_ | -1.8 | 190.0849  383.1941 226.1419  271.1429  297.1599 | [C_11_H_11_NO_2_]+H^+^  [C_22_H_27_N_2_O_4_]^+^ [C_12_H_19_NO_3_]+H^+^  [C_16_H_18_N_2_O_2_]+H^+^  [C_18_H_23_N_2_O_2_-2H]^+^ | *7-hydroxymitragynine | 1 | Standard | 1.00 | 3.3 | 1.7 | 1.5E-03 | 3.0E-03 | 0.254 ± 0.054 | 0.841 ± 0.086 |
| 37 | 5.20 | 353.185 | C_21_H_24_N_2_O_3_ | -2.7 | - | - | ^∆^*Ajmalicine | 3 | KEGG | 0.79 | 0.4 | -1.2 | 2.5E-03 | 4.8E-03 | 0.054 ± 0.008 | 0.023 ± 0.002 |
| 38 | 5.21 | 385.2113 | C_22_H_28_N_2_O_4_ | -2.3 | 160.0735  226.1439  238.1415  353.1858  314.1329 | [C_10_H_9_NO]+H^+^  [C_12_H_19_NO_3_]+H^+^  [C_13_H_21_NO_3_-H]^+^  [C_21_H_25_N_2_O_3_]^+^  [C_18_H_20_NO_4_]^+^ | **Rynchophylline | 2 | KEGG | 1.43 | 0.1 | -3.4 | 6.2E-07 | 5.3E-06 | 5.740 ± 0.785 | 0.559 ± 0.037 |
| 40 | 5.31 | 369.2198 | C_22_H_28_N_2_O_3_ | 6.9 | 144.0818  170.0965  226.1441  238.1433  337.1917 | [C_10_H_9_N]+H^+^  [C_12_H_12_N]^+^  [C_12_H_19_NO_3_]+H^+^  [C_13_H_21_NO_3_-H]^+^  [C_21_H_25_N_2_O_2_]^+^ | **Corynantheidine | 2 | ChEBI | 1.26 | 0.2 | -2.6 | 2.6E-05 | 9.2E-05 | 7.670 ± 0.297 | 1.300 ± 0.282 |
| 41 | 5.31 | 383.196 | C_22_H_26_N_2_O_4_ | -1.3 | - | - | **Akuammine,  Aricine,  Cabucine,  Lochnerinine | 3 | KEGG | 1.41 | 0.1 | -3.4 | 5.5E-06 | 2.5E-05 | 0.835 ± 0.153 | 0.081 ± 0.003 |
| 42 | 5.34 | 365.1858 | C_22_H_24_N_2_O_3_ | -0.4 | 197.1064  249.1368  298.1427  279.1130 | [C_13_H_15_N_2_-2H]^+^  [C_17_H_17_N_2_]^+^  [C_18_H_18_NO_3_+H]+H^+^  [C_17_H_15_N_2_O_2_]^+^ | **11-Methoxy-vinorine | 2 | KEGG | 2.12 | 0.0 | -7.3 | 1.0E-09 | 2.9E-08 | 1.202 ± 0.167 | 0.007 ± 0.000 |
| 43 | 5.35 | 353.185 | C_21_H_24_N_2_O_3_ | -2.7 | - | - | ^∆^**Ajmalicine | 3 | KEGG | 1.30 | 0.2 | -2.7 | 1.4E-06 | 8.8E-06 | 0.032 ± 0.004 | n.d. |
| 44 | 5.37 | 397.213 | C_23_H_28_N_2_O_4_ | 2.1 | - | - | Paynantheine | 3 | PubChem  KNApSAcK | 0.27 | 1.1 | 0.2 | 2.2E-01 | 2.6E-01 | 5.005 ± 0.472 | 5.667 ± 0.243 |
| 45 | 5.38 | 335.174 | C_21_H_22_N_2_O_2_ | -4.2 | - | - | Strychnine,  Vinorine | 3 | KEGG | 0.34 | 1.2 | 0.3 | 6.6E-02 | 9.5E-02 | 0.010 ± 0.001 | 0.012 ± 0.001 |
| 47 | 5.39 | 367.2031 | C_22_H_26_N_2_O_3_ | 4.0 | 251.1542  197.1072  226.1431  144.0815  238.1421 | [C_17_H_19_N_2_]+  [C_13_H_14_N_2_-H]+  [C_12_H_18_NO_3_+H]+H^+^  [C_10_H_9_N]+H^+^  [C_13_H_19_NO_3_]+H^+^ | **Hirsuteine | 2 | KEGG | 1.92 | 0.0 | -6.0 | 1.1E-11 | 9.3E-10 | 5.749 ± 1.206 | 0.124 ± 0.011 |
| 48 | 5.46 | 399.2286 | C_23_H_30_N_2_O_4_ | 1.9 | 174.0918  226.1438  238.1435  328.1546  367.2012 | [C_11_H_11_NO]+H^+^  [C_12_H_19_NO_3_]+H^+^  [C_13_H_21_NO_3_-H]^+^  [C_19_H_22_NO_4_]^+^  [C_22_H_27_N_2_O_3_]^+^ | *Mitragynine | 1 | Standard | 0.36 | 1.2 | 0.3 | 1.0E-04 | 3.2E-04 | 9.469 ± 0.155 | 11.278 ± 0.208 |
| 51 | 5.53 | 397.2136 | C_23_H_28_N_2_O_4_ | 3.6 | 174.0915  238.1424  367.2003  328.1534  200.1061 | [C_11_H_11_NO]+H^+^  [C_13_H_19_NO_3_]+H^+^  [C_22_H_25_N_2_O_3_+H]+H^+^  [C_19_H_22_NO_4_]^+^  [C_13_H_14_NO]^+^ | *Isopaynantheine | 2 | PubChem  KNApSAcK | 0.91 | 0.4 | -1.3 | 4.2E-07 | 4.5E-06 | 9.044 ± 0.192 | 3.568 ± 0.218 |
| 52 | 5.55 | 395.1944 | C_23_H_26_N_2_O_4_ | -5.4 | 227.1179  281.1635  309.1252  381.1788  382.1862 | [C_14_H_16_N_2_O-H]^+^  [C_18_H_20_N_2_O]+H^+^  [C_22_H_24_N_2_O_4_+H]+H^+^  [C_22_H_23_N_2_O_4_+H]+H^+^  [C_18_H_18_N_2_O_3_-H]^+^ | **Brucine | 2 | KEGG | 1.38 | 0.1 | -3.1 | 1.8E-06 | 8.8E-06 | 0.805 ± 0.103 | 0.093 ± 0.010 |
| 73 | 7.94 | 399.230 | C_23_H_30_N_2_O_4_ | 5.4 | - | - | *Speciociliatine | 3 | KNApSAcK | 0.64 | 1.7 | 0.8 | 5.1E-03 | 8.8E-03 | 0.008 ± 0.001 | 0.014 ± 0.001 |
| 77 | 8.38 | 351.171 | C_21_H_22_N_2_O_3_ | 1.9 | - | - | **Perakine,  Vomilenine,  Polyneuridine aldehyde,  19-epi-Cathenamine,  Cathenamine | 3 | KEGG | 1.76 | 0.0 | -6.6 | 8.8E-04 | 2.2E-03 | 0.958 ± 0.495 | n.d. |
| 78 | 8.53 | 349.1558 | C_21_H_20_N_2_O_3_ | 3.2 | 321.1573  333.1228  334.1312 | [C_20_H_19_N_2_O_2_+H]+H^+^  [C_20_H_17_N_2_O_3_]^+^  [C_20_H_17_N_2_O_3_]+H^+^ | **Alstonine | 2 | KEGG | 1.25 | 0.0 | -4.5 | 2.0E-02 | 3.0E-02 | 0.157 ± 0.088 | n.d. |
| 79 | 8.53 | 381.1808 | C_22_H_24_N_2_O_4_ | -0.2 | 349.1540  321.1591  322.1651 | [C_21_H_20_N_2_O_3_]+H^+^  [C_20_H_21_N_2_O_2_]^+^  [C_20_H_21_N_2_O_2_]+H^+^ | **Vomicine | 2 | KEGG | 1.99 | 0.0 | -8.0 | 2.3E-04 | 6.3E-04 | 1.480 ± 0.805 | 0.006 ± 0.002 |
| **Quinoline alkaloids** | | |  |  |  |  |  |  |  |  |  |  |  |  |  |  |
| 7 | 1.85 | 411.147 | C_22_H_22_N_2_O_6_ | 19 | - | - | *Edulitine | 3 | ChEBI | 1.09 | 0.3 | -2.0 | 3.4E-05 | 1.1E-04 | 0.391 ± 0.037 | 0.099 ± 0.013 |
| 16 | 4.64 | 305.157 | C_17_H_22_NO_4_ | -16.9 | - | - | Balfourodinium | 3 | KEGG | 0.20 | 1.0 | 0.1 | 1.1E-01 | 1.4E-01 | 0.049 ± 0.002 | 0.054 ± 0.001 |
| 80 | 8.60 | 484.384 | C_30_H_49_N_3_O_2_ | -11.9 | - | - | Spirolucidine,  Oxolucidine B | 3 | KEGG | 0.20 | 0.9 | -0.2 | 2.9E-01 | 3.2E-01 | 0.089 ± 0.006 | 0.080 ± 0.002 |
| 84 | 9.68 | 250.088 | C_16_H_11_NO_2_ | 7.0 | - | - | Dubamine | 3 | KEGG | 0.08 | 1.0 | 0.0 | 4.8E-01 | 5.0E-01 | 0.007 ± 0.000 | 0.008 ± 0.000 |
| **Isoquinoline alkaloids** | | |  |  |  |  |  |  |  |  |  |  |  |  |  |  |
| 12 | 4.53 | 372.1430 | C_20_H_21_NO_6_ | -3.1 | 177.0555  165.0554 | [C_10_H_9_O_3_]^+^  [C_9_H_9_O_3_]^+^ | **(+)-N-(methoxycarbonyl)-N-norboldine | 2 | ChEBI | 1.34 | 7.2 | 2.8 | 2.0E-07 | 2.4E-06 | n.d. | 0.057 ± 0.007 |
| 15 | 4.63 | 321.129 | C_19_H_16_N_2_O_3_ | 17.5 | - | - | Alangimarine | 3 | KEGG | 0.26 | 1.2 | 0.2 | 7.8E-02 | 1.0E-01 | 0.008 ± 0.000 | 0.010 ± 0.000 |
| 68 | 7.58 | 180.103 | C_10_H_13_NO_2_ | 6.1 | - | - | Salsolinol | 3 | KEGG | 0.23 | 1.1 | 0.2 | 3.1E-01 | 3.4E-01 | 0.018 ± 0.002 | 0.020 ± 0.001 |
| 71 | 7.84 | 611.305 | C_37_H_42_N_2_O_6_ | -10.7 | - | - | *Isoliensinine, Liensinine | 3 | ChEBI | 0.19 | 1.1 | 0.1 | 1.4E-01 | 1.7E-01 | 0.022 ± 0.001 | 0.025 ± 0.001 |
| 74 | 8.02 | 194.117 | C_11_H_15_NO_2_ | -2.9 | - | - | Salsoline,  Heliamine | 3 | ChEBI | 0.16 | 1.1 | 0.1 | 5.7E-01 | 5.9E-01 | 0.141 ± 0.015 | 0.150 ± 0.009 |
| 75 | 8.07 | 625.321 | C_38_H_44_N_2_O_6_ | -9.9 | - | - | Dauricine,  Neferine | 3 | KEGG | 0.06 | 1.0 | 0.0 | 7.2E-01 | 7.3E-01 | 0.028 ± 0.002 | 0.029 ± 0.001 |
| **Quinolizidine alkaloid** | | |  |  |  |  |  |  |  |  |  |  |  |  |  |  |
| 24 | 4.91 | 431.2156 | C_23_H_30_N_2_O_6_ | -4.8 | 413.2090 | [C_23_H_29_N_2_O_5_]^+^ | Cinegalline | 2 | KEGG | 0.71 | 1.9 | 0.9 | 7.8E-03 | 1.2E-02 | 0.030 ± 0.005 | 0.048 ± 0.010 |
| **Quinazoline alkaloid** | | |  |  |  |  |  |  |  |  |  |  |  |  |  |  |
| 14 | 4.58 | 205.0949 | C_11_H_12_N_2_O_2_ | -11.0 | 146.0588 | [C_9_H_10_NO-2H]^+^ | Vasicinol | 3 | KEGG | 0.61 | 0.6 | -0.8 | 7.0E-02 | 9.7E-02 | 0.064 ± 0.011 | 0.037 ± 0.011 |
| **Terpenoid alkaloids** | | |  |  |  |  |  |  |  |  |  |  |  |  |  |  |
| 63 | 5.99 | 528.367 | C_32_H_49_NO_5_ | -2.6 | - | - | *Daphniphylline | 3 | ChEBI | 0.84 | 2.4 | 1.2 | 6.0E-03 | 9.9E-03 | 0.005 ± 0.001 | 0.011 ± 0.001 |
| **Cyclopeptide alkaloids** | | | |  |  |  |  |  |  |  |  |  |  |  |  |  |
| 83 | 9.38 | 501.340 | C_28_H_44_N_4_O_4_ | -7.0 | - | - | Adouetine X,  Frangulanine | 3 | KEGG | 0.37 | 1.3 | 0.3 | 1.4E-01 | 1.7E-01 | 0.631 ± 0.092 | 0.793 ± 0.040 |
| 85 | 12.57 | 429.244 | C_23_H_32_N_4_O_4_ | -13.1 | - | - | *Nummularine F | 3 | ChEBI | 0.25 | 1.1 | 0.2 | 2.9E-03 | 5.4E-03 | 0.025 ± 0.001 | 0.028 ± 0.001 |
| **Peptide alkaloid** | | |  |  |  |  |  |  |  |  |  |  |  |  |  |  |
| 81 | 8.97 | 487.325 | C_27_H_42_N_4_O_4_ | -6.0 | - | - | Hovenine A | 3 | ChEBI | 0.42 | 1.3 | 0.4 | 7.9E-02 | 1.0E-01 | 0.191 ± 0.026 | 0.252 ± 0.013 |
| **Harmala alkaloids** | | |  |  |  |  |  |  |  |  |  |  |  |  |  |  |
| 9 | 2.10 | 261.084 | C_13_H_12_N_2_O_4_ | -11.4 | - | - | **1,2,3,4-Tetrahydro-β-carboline-1,3-dicarboxylic acid | 3 | ChEBI | 1.32 | 6.7 | 2.7 | 9.3E-11 | 4.0E-09 | n.d. | 0.032 ± 0.001 |
| **Phenantridine alkaloid** | | | |  |  |  |  |  |  |  |  |  |  |  |  |  |
| 4 | 1.80 | 258.109 | C_15_H_15_NO_3_ | -13.4 | - | - | Ismine | 3 | ChEBI | 0.39 | 1.2 | 0.3 | 2.6E-01 | 3.0E-01 | 0.026 ± 0.001 | 0.031 ± 0.001 |
| **Piperamide alkaloid** | | |  |  |  |  |  |  |  |  |  |  |  |  |  |  |
| 19 | 4.77 | 344.228 | C_21_H_29_NO_3_ | 17.4 | - | - | Piperolein B,  Isopiperolein B | 3 | PubChem | 0.15 | 1.1 | 0.1 | 3.7E-01 | 4.0E-01 | 0.031 ± 0.002 | 0.033 ± 0.001 |
| **Piperidine alkaloids** | | |  |  |  |  |  |  |  |  |  |  |  |  |  |  |
| 8 | 1.94 | 130.087 | C_6_H_11_NO_2_ | 5.7 | - | - | **L-Pipecolic acid,  D-Pipecolic acid | 3 | ChEBI | 1.32 | 0.1 | -2.9 | 1.2E-04 | 3.7E-04 | 0.043 ± 0.007 | 0.006 ± 0.002 |
| 76 | 8.37 | 288.253 | C_16_H_33_NO_3_ | -1.1 | - | - | *Prosopinine | 3 | KEGG | 0.44 | 1.3 | 0.4 | 1.5E-03 | 3.0E-03 | 0.055 ± 0.003 | 0.072 ± 0.002 |
| 86 | 12.85 | 132.103 | C_6_H_13_NO_2_ | 8.3 | - | - | *6-Deoxyfagomine | 3 | ChEBI | 0.33 | 1.2 | 0.3 | 5.3E-03 | 9.0E-03 | 0.014 ± 0.000 | 0.017 ± 0.001 |
| **Purine alkaloids** | | |  |  |  |  |  |  |  |  |  |  |  |  |  |  |
| 3 | 1.78 | 181.071 | C_7_H_8_N_4_O_2_ | -5.5 | - | - | **Theophylline,  Theobromine,  Paraxanthine | 3 | KEGG | 1.32 | 7.1 | 2.8 | 1.8E-06 | 8.8E-06 | 0.021 ± 0.003 | 0.150 ± 0.011 |
| 6 | 1.85 | 195.0859 | C_8_H_10_N_4_O_2_ | -9.0 | 138.0675  135.0443 | [C_6_H_7_N_3_O]+H^+^  [C_6_H_7_N_3_O-2H]^+^ | *Caffeine | 2 | KEGG | 0.61 | 0.6 | -0.8 | 7.2E-03 | 1.1E-02 | 0.297 ± 0.035 | 0.177 ± 0.013 |
| **Pyrazine alkaloid** | | |  |  |  |  |  |  |  |  |  |  |  |  |  |  |
| 33 | 5.18 | 243.0857 | C_12_H_10_N_4_O_2_ | -8.0 | 165.0542 | [C_7_H_6_N_3_O_2_]+H^+^ | **Lumichrome | 2 | KEGG | 1.53 | 13.9 | 3.8 | 1.7E-06 | 8.8E-06 | 0.047 ± 0.008 | 0.652 ± 0.050 |
| **Unclassified** | | |  |  |  |  |  |  |  |  |  |  |  |  |  |  |
| 5 | 1.84 | 322.147 | C_20_H_19_NO_3_ | 10.0 | - | - | **Acronycine,  2-[4-(3,4-Methylenedioxyphenyl)butyl]-4(1H)-quinolinone | 3 | KEGG | 1.34 | 6.9 | 2.8 | 2.1E-09 | 4.5E-08 | n.d. | 0.012 ± 0.001 |
| 36 | 5.18 | 569.312 | C_34_H_40_N_4_O_4_ | 0 | - | - | Ceanothine E,  Adouetine Y | 3 | ChEBI | 0.20 | 1.1 | 0.2 | 2.0E-01 | 2.4E-01 | 0.010 ± 0.001 | 0.011 ± 0.001 |
| 46 | 5.39 | 238.141 | C_13_H_19_NO_3_ | -11.6 | - | - | *Anhalonine,  Pellotine,  Gigantine,  O-7-Angelylheliotridine | 3 | KEGG | 0.61 | 1.6 | 0.7 | 2.1E-03 | 4.0E-03 | 0.013 ± 0.001 | 0.022 ± 0.001 |
| 66 | 6.21 | 257.166 | C_16_H_20_N_2_O | 4.5 | - | - | *Huperzine B,  Chanoclavine-I,  Fumigaclavine B | 3 | KEGG | 0.36 | 1.2 | 0.3 | 8.9E-04 | 2.2E-03 | 0.006 ± 0.000 | 0.008 ± 0.000 |
| 69 | 7.63 | 597.293 | C_36_H_40_N_2_O_6_ | -4.8 | - | - | Guattegaumerine,  Dipiperamide D,  Berbamunine | 3 | KEGG | 0.29 | 1.2 | 0.2 | 4.4E-03 | 7.7E-03 | 0.011 ± 0.000 | 0.013 ± 0.000 |
| 70 | 7.77 | 225.195 | C_13_H_24_N_2_O | -5.1 | - | - | *Anapheline,  Cuscohygrine | 3 | KEGG | 0.65 | 1.7 | 0.8 | 3.9E-04 | 1.1E-03 | 0.016 ± 0.001 | 0.028 ± 0.002 |

^a^Peak numbers (N) are assigned according to the elution order of the base peak chromatogram (BPC).

^b^Level of identification (ID level), 1: identification verified using authentic standard at fragmentation level; 2: identification done at fragmentation level (MS/MS) by matching with online databases; 3: identification done using parent ion (m/z value) only due to absence of fragments.

^∆^Several compounds are listed twice or more with similar m/z value but with different RT potentially due to 1) different isomers that may be present and separate on the chromatography column to yield different RTs for the same compounds; 2) limitation of online metabolite databases with limited information on the metabolites of *M. speciosa*.

^c^Significant metabolites with false discovery rate (FDR) value < 0.05 are asterisked (*); |Log_2_FC| > 2 are double-asterisked (**).

^d^Relative quantification and statistical data of alkaloids in young (Y) and mature (M) leaves of *M. speciosa*. Each value is the mean of five biological replicates and five technical replicates [mean ± standard error of the mean (SEM)]. Where compounds may be undetected or absent, the value is considered not detected (n.d.).
