# Supplementary material for: Comparative metabolomics analysis reveals alkaloid repertoires in young and mature Mitragyna speciosa (Korth.) Havil. Leaves
Source: PLoS One. 2023 Mar 21;18(3):e0283147. doi: 10.1371/journal.pone.0283147 (PMC10030037; doi:10.1371/journal.pone.0283147)
Supplement: S4 Table — (DOCX) [file pone.0283147.s007.docx]

**S4 Table. Putative identification of other secondary metabolites in the young (Y) and mature (M) leaves of M. speciosa**

| **Peak no.^a^**  **(N)** | **RT, min** | **Parent ion**  **(*m/z*)**  **[M+H]^+^** | **Molecular formula** | **Mass error**  **(ppm)** | **Fragment ions**  **[M+H]^+^** | **Fragment formula** | **Metabolite ID** | **ID level^b^** | **Reference** | **VIP** | **FC** | **Log_2_FC^c^** | **P-value** | **FDR^c^** | **Rel. abundance^d^**  **(Mean ± SEM)** | |
| --- | --- | --- | --- | --- | --- | --- | --- | --- | --- | --- | --- | --- | --- | --- | --- | --- |
|  |  |  |  |  |  |  |  |  |  |  |  |  |  |  | **Y** | **M** |
| **Carboxylic acid** | | | |  |  |  |  |  |  |  |  |  |  |  |  |  |
| 10 | 2.31 | 193.0702 | C_7_H_12_O_6_ | -2.4 | 129.0534  157.0489 | [C_6_H_10_O_3_-H]^+^  [C_7_H_10_O_4_-H]^+^ | Quinate | 2 | KEGG | 0.31 | 1.2 | 0.3 | 2.5E-01 | 2.9E-01 | 0.595 ± 0.085 | 0.729 ± 0.073 |
| **Glucoside** | | | |  |  |  |  |  |  |  |  |  |  |  |  | |
| 1 | 1.59 | 332.1334 | C_14_H_21_NO_8_ | -1.8 | 152.0721  314.1229  315.1314 | [C_8_H_10_NO_2_]^+^  [C_14_H_20_NO_7_]^+^  [C_14_H_20_NO_7_]+H^+^ | **5'-O-beta-D-Glucosylpyridoxine | 2 | ChEBI | 1.92 | 64.7 | 6.0 | 1.4E-07 | 2.0E-06 | 0.002 ± 0.000 | 0.144 ± 0.023 |
| **Phenol** | | | |  |  |  |  |  |  |  |  |  |  |  |  | |
| 2 | 1.77 | 361.1340 | C_18_H_20_N_2_O_6_ | -15.0 | 181.0716  163.0610 | [C_6_H_11_O_6_+H]+H^+^  [C_6_H_11_O_5_]^+^ | **3-Methoxytyramine-betaxanthin | 2 | KEGG | 1.67 | 22.7 | 4.5 | 1.7E-06 | 8.8E-06 | 0.010 ± 0.003 | 0.226 ± 0.027 |
| **Phenylpropanoids** | | | |  |  |  |  |  |  |  |  |  |  |  |  | |
| 31 | 5.18 | 165.0548 | C_9_H_8_O_3_ | 1.1 | 133.0296  119.0495  135.0448  105.0323 | [C_8_H_6_O_2_-H]^+^  [C_8_H_6_O]+H^+^  [C_8_H_7_O_2_]^+^  [C_7_H_5_O]^+^ | *Caffeic aldehyde | 2 | KEGG | 0.82 | 2.3 | 1.2 | 1.4E-03 | 3.0E-03 | 0.150 ± 0.021 | 0.346 ± 0.029 |
| 32 | 5.18 | 225.0759 | C_11_H_12_O_5_ | -3.3 | 147.0428  151.0375  165.0531  175.0373 | [C_9_H_8_O_2_-H]^+^  [C_8_H_7_O_3_]^+^  [C_9_H_8_O_3_]+H^+^  [C_10_H_8_O_3_-H]^+^ | **Sinapate | 2 | KEGG | 1.57 | 15.6 | 4.0 | 5.8E-07 | 5.3E-06 | 0.020 ± 0.003 | 0.311 ± 0.018 |
| 72 | 7.86 | 415.2102 | C_24_H_30_O_6_ | -3.2 | 119.0847 | [C_9_H_9_+H]+H^+^ | Magnoshinin | 2 | KEGG | 0.16 | 0.9 | -0.1 | 4.8E-01 | 5.0E-01 | 0.042 ± 0.004 | 0.038 ± 0.004 |
| **Terpenoids** | | | |  |  |  |  |  |  |  |  |  |  |  |  | |
| 39 | 5.27 | 389.144 | C_17_H_24_O_10_ | 0 | - | - | Secologanin | 3 | KEGG | 0.52 | 0.6 | -0.7 | 6.7E-02 | 9.5E-02 | 0.645 ± 0.078 | 0.405 ± 0.067 |
| 60 | 5.91 | 495.2215 | C_25_H_34_O_10_ | -2.0 | 419.2062  421.2192 | [C_23_H_31_O_7_]+  [C_23_H_31_O_7_+H]+H^+^ | **Glaucarubinone/ Soularubinone | 2 | KEGG | 1.63 | 0.0 | -5.7 | 1.5E-03 | 3.0E-03 | 0.119 ± 0.061 | n.d. |
| 61 | 5.99 | 469.3303 | C_30_H_44_O_4_ | -2.0 | 189.1625  135.1170  423.3230 | [C_14_H_21_]^+^  [C_10_H_17_-2H]^+^  [C_30_H_43_O_3_]^+^ | *3-Oxoglycyrrhetinate | 2 | KEGG | 0.91 | 2.7 | 1.4 | 6.9E-03 | 1.1E-02 | 0.291 ± 0.071 | 0.777 ± 0.067 |
| 62 | 5.99 | 487.3405 | C_30_H_46_O_5_ | -2.7 | 469.3303  135.1177  470.3364  163.1482  191.1809 | [C_30_H_45_O_4_]^+^  [C_10_H_17_-2H]^+^  [C_30_H_45_O_4_]+H^+^  [C_12_H_20_-H]^+^  [C_14_H_23_]^+^ | *Glabric acid | 2 | KEGG | 0.89 | 2.6 | 1.4 | 6.9E-03 | 1.1E-02 | 0.222 ± 0.053 | 0.577 ± 0.048 |
| 67 | 6.45 | 469.3309 | C_30_H_44_O_4_ | -0.7 | 135.1178  203.1792  451.3200 | [C_10_H_14_]+H^+^  [C_15_H_21_+H]+H^+^  [C_30_H_43_O_3_]^+^ | *Fatsicarpain C | 2 | ChEBI | 0.97 | 3.1 | 1.6 | 3.1E-03 | 5.7E-03 | 0.125 ± 0.029 | 0.390 ± 0.044 |
| 82 | 9.37 | 605.416 | C_35_H_56_O_8_ | 18.5 | - | - | *Papyriferic acid | 2 | ChEBI | 0.53 | 1.5 | 0.6 | 9.4E-03 | 1.4E-02 | 0.010 ± 0.001 | 0.015 ± 0.001 |
| **Flavonoids** | | | |  |  |  |  |  |  |  |  |  |  |  |  | |
| 50 | 5.50 | 291.0858 | C_15_H_14_O_6_ | -1.8 | 139.0396  123.0452  207.0647  147.0437  165.0537 | [C_7_H_6_O_3_]+H^+^  [C_7_H_6_O_2_]+H^+^  [C_11_H_10_O_4_]+H^+^  [C_9_H_9_O_2_-2H]^+^  [C_9_H_8_O_3_]+H^+^ | *Epicatechin | 2 | PubChem | 0.62 | 1.6 | 0.7 | 4.5E-04 | 1.2E-03 | 2.314 ± 0.174 | 3.735 ± 0.135 |
| 53 | 5.62 | 303.0492 | C_15_H_10_O_7_ | -2.4 | 273.0372  247.0602 | [C_14_H_10_O_6_-H]^+^  [C_13_H_9_O_5_+H]+H^+^ | *Quarcetin/  6-Hydroxykaempferol/ Robinetin/  Morin/  8-Hydroxykaempferol | 2 | KEGG | 1.04 | 3.5 | 1.8 | 3.4E-05 | 1.1E-04 | 0.077 ± 0.011 | 0.261 ± 0.022 |
| 54 | 5.62 | 611.1602 | C_27_H_30_O_16_ | -0.8 | 127.0381  303.0491  305.0525 | [C_6_H_4_O_3_+2H]+H^+^  [C_15_H_9_O_7_+H]+H^+^  [C_11_H_15_O_10_-2H]^+^ | *Rutin | 2 | KEGG | 1.03 | 3.4 | 1.8 | 2.7E-05 | 9.2E-05 | 0.622 ± 0.084 | 2.137 ± 0.120 |
| 55 | 5.73 | 579.1489 | C_30_H_26_O_12_ | -1.4 | 287.0534  409.0908  127.0397  123.0444  271.0588 | [C_15_H_11_O_6_]^+^  [C_22_H_18_O_8_-H]^+^  [C_6_H_6_O_3_]+H^+^  [C_7_H_6_O_2_]+H^+^  [C_15_H_12_O_5_-H]^+^ | *Procyanidin B4, Procyanidin B5 | 2 | KEGG | 0.80 | 2.2 | 1.1 | 1.2E-03 | 2.7E-03 | 0.241 ± 0.036 | 0.525 ± 0.020 |
| 56 | 5.75 | 287.0536 | C_15_H_10_O_6_ | -4.9 | 139.0400 | [C_7_H_5_O_3_+H]+H^+^ | *2''-Hydroxygenistein,  Orobol | 2 | KEGG | 0.58 | 1.6 | 0.7 | 4.0E-03 | 7.3E-03 | 0.067 ± 0.006 | 0.108 ± 0.009 |
| 57 | 5.75 | 595.1656 | C_27_H_30_O_15_ | -0.2 | 287.0542  289.0581  129.0549 | [C_15_H_9_O_6_+H]+H^+^  [C_11_H_15_O_9_-2H]^+^  [C_6_H_10_O_3_-H]^+^ | *Isoorientin 2''-O-rhamnoside | 2 | KEGG | 0.67 | 1.8 | 0.8 | 1.1E-03 | 2.5E-03 | 0.381 ± 0.033 | 0.682 ± 0.060 |
| 58 | 5.86 | 303.0488 | C_15_H_10_O_7_ | -3.7 | 247.0562 | [C_13_H_9_O_5_+H]+H^+^ | **Quercetin/  6-Hydroxykaempferol/ Robinetin/  Morin/  8-Hydroxykaempferol | 2 | KEGG | 1.56 | 15.8 | 4.0 | 9.5E-06 | 3.9E-04 | 0.010 ± 0.005 | 0.456 ± 0.058 |
| 59 | 5.86 | 465.1023 | C_21_H_20_O_12_ | -1.0 | 145.051 | [C_6_H_10_O_4_-H]^+^ | **Myricitrin/  Bracteatin 6-O-glucoside/  Gossypetin 8-rhamnoside | 2 | KEGG | 1.57 | 16.8 | 4.1 | 1.7E-05 | 6.5E-05 | 0.086 ± 0.018 | 1.441 ± 0.189 |
| 64 | 6.05 | 449.1082 | C_21_H_20_O_11_ | 0.8 | 287.0544 | [C_15_H_9_O_6_+H]+H^+^ | **Carthamone | 2 | KEGG | 1.30 | 7.0 | 2.8 | 1.7E-06 | 8.8E-06 | 0.048 ± 0.005 | 0.333 ± 0.032 |
| 65 | 6.20 | 453.1167 | C_24_H_20_O_9_ | -2.9 | 191.0328  301.0726  163.0396  123.0451  192.0423 | [C_10_H_7_O_4_]^+^  [C_16_H_12_O_6_]+H^+^  [C_9_H_8_O_3_-H]^+^  [C_7_H_6_O_2_]+H^+^  [C_10_H_7_O_4_]+H^+^ | **Cinchonain 1a | 2 | KEGG | 1.49 | 12.8 | 3.7 | 1.3E-06 | 8.8E-06 | n.d. | 0.118 ± 0.018 |
| **Phenolic aldehyde** | | | |  |  |  |  |  |  |  |  |  |  |  |  | |
| 49 | 5.50 | 139.0398 | C_7_H_6_O_3_ | 6.0 | 111.0431 | [C_6_H_5_O_2_+H]+H^+^ | *3,4-Dihydroxybenzaldehyde | 2 | KEGG | 0.59 | 1.6 | 0.6 | 9.8E-04 | 2.3E-03 | 0.230 ± 0.018 | 0.342 ± 0.012 |

^a^Peak numbers are assigned according to the elution order of the base peak chromatogram (BPC).

^b^Level of identification (ID level), 1: identification verified using authentic standard; 2: identification done at fragmentation level (MS/MS) by matching with online libraries; 3: identification done using parent ion (m/z value) only due to absence of fragments.

^c^Significant metabolites with false discovery rate (FDR) value < 0.05 are asterisked (*); |Log_2_FC| > 2 are double-asterisked (**).

^d^Relative quantification and statistical data of alkaloids in young (Y) and mature (M) leaves of *M. speciosa*. Each value is the mean of five biological replicates and five technical replicates [mean ± standard error of the mean (SEM)]. Where compounds may be undetected or absent, the value is considered not detected (n.d.).
