# Supplementary material for: Comparative metabolomics analysis reveals alkaloid repertoires in young and mature Mitragyna speciosa (Korth.) Havil. Leaves
Source: PLoS One. 2023 Mar 21;18(3):e0283147. doi: 10.1371/journal.pone.0283147 (PMC10030037; doi:10.1371/journal.pone.0283147)
Supplement: S1 Text — (DOCX) [file pone.0283147.s008.docx]

**Authentication of plant material**

Authentication of *M. speciosa* leaves used in this study was done based on previous studies [1]. The DNA of 140 mg powdered *M. speciosa* leaves was extracted using the DNeasy Plant Kit (QIAGEN, Germany). DNA quality and quantity were determined using NanoDrop® ND-1000 (Thermo Scientific). The extracted total DNA was kept at -20°C for PCR analysis. This study used forward (18S-25S-5’F) and reverse primers (18S-25S-3’R), 5’-GTA GGT GAA CCT GCA GAA GGA TCA-3’ and 5’-CCA TGC TTA AAC TCA GCG GGT-3’, respectively, to amplify approximately 650 bp ITS1-5.8S-ITS2 region.

PCR amplification was performed using a T-100 (Bio-Rad) thermal cycler under cycling profile of a preliminary denaturation at 95°C for 3 min, 39 cycles of 95 °C for 40 s, 55 °C for 40 s, and 72 °C for 1 min, and a final extension at and 72 °C for 10 min. The PCR products were then determined by agarose gel electrophoresis and purified using QIA quick PCR purification kit (QIAGEN, Germany) prior sequencing. The obtained sequences were checked, edited and aligned by BioEdit (version 7.2.5) [2]. The plant materials used in this study were identified by comparison of Basic Local Alignment Search Tool (BLAST) results demonstrating ≥ 99% sequence similarity to the published sequences of *M. speciosa* (Table A).

**Table A.** **Blast results summary of ITS region showing that the obtained sequences were identical to the published ITS sequences of M. speciosa.**

| **Description** | **Species name** | **Identity (%)** | **GenBank**  **Accession** |
| --- | --- | --- | --- |
| *Mitragyna speciosa* isolation-source red-veined variety 18S ribosomal RNA gene | *Mitragyna speciosa* | 99.85 | JF412826.1 |
| *Mitragyna speciosa* clone K51 Indonesian small subunit ribosomal RNA gene | *Mitragyna speciosa* | 99.7 | MT586314.1 |
| *Mitragyna speciosa* strain White Jongkong clone K52_2 small subunit ribosomal RNA gene | *Mitragyna speciosa* | 99.7 | MT111843.1 |

**References**

1. Sukrong S, Zhu S, Ruangrungsi N, Phadungcharoen T, Palanuvej C, Komatsu K. Molecular analysis of the genus *Mitragyna* existing in Thailand based on rDNA its sequences and its application to identify a narcotic species: *Mitragyna speciosa*. Biol Pharm Bull. 2007;30: 1284–1288. doi:10.1248/bpb.30.1284

2. Hall TA. BioEdit: a user-friendly biologiccal sequence alignment editor and analysis program for Windows 95/98/NT. Nucleic Acids Symp Ser. 1999;41: 95–98. doi:10.1039/c7qi00394c
