# Supplementary material for: Comparative metabolomics analysis reveals alkaloid repertoires in young and mature Mitragyna speciosa (Korth.) Havil. Leaves
Source: PLoS One. 2023 Mar 21;18(3):e0283147. doi: 10.1371/journal.pone.0283147 (PMC10030037; doi:10.1371/journal.pone.0283147)
Supplement: S1 Appendix — (DOCX) [file pone.0283147.s009.docx]

**S1 Appendix.** LC-ESI-TOF-MS spectra of metabolite features putatively identified at ID level 3 (identification done using m/z value only due to absence of fragments).


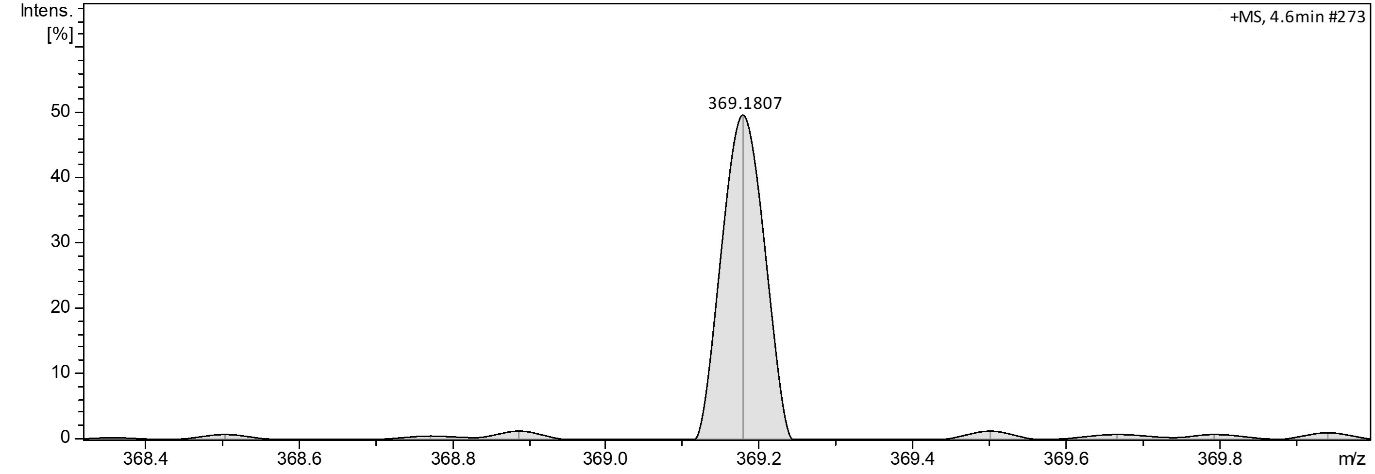


**Fig S1** a. Spectrum view of mitraphyline/ isomitraphyline/ strictosidine aglycone/ horhammericine/ dialdehyde1


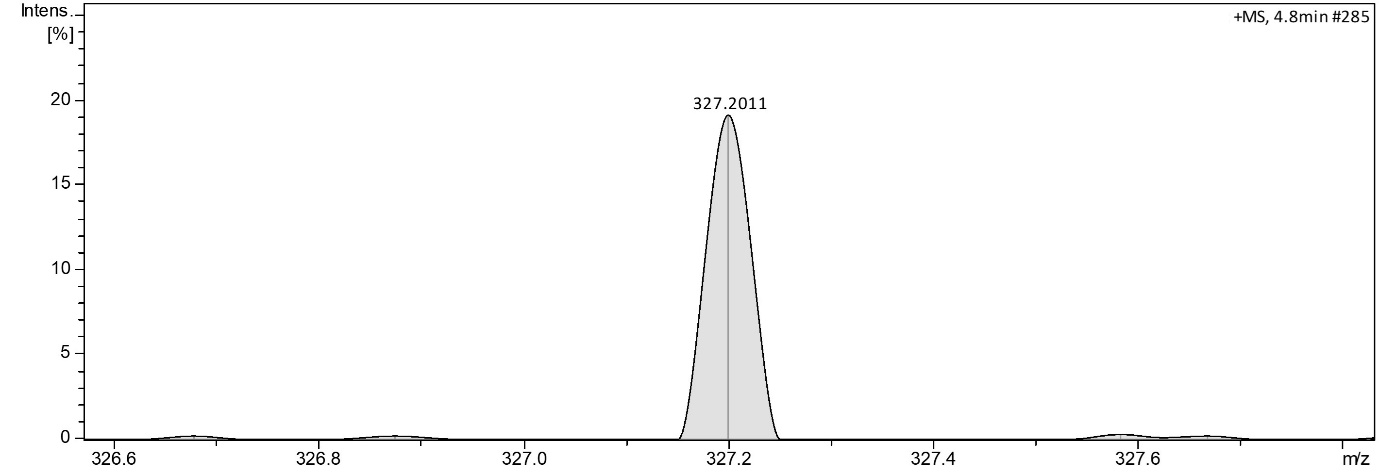


**Fig S1** b. Spectrum view of ajmaline


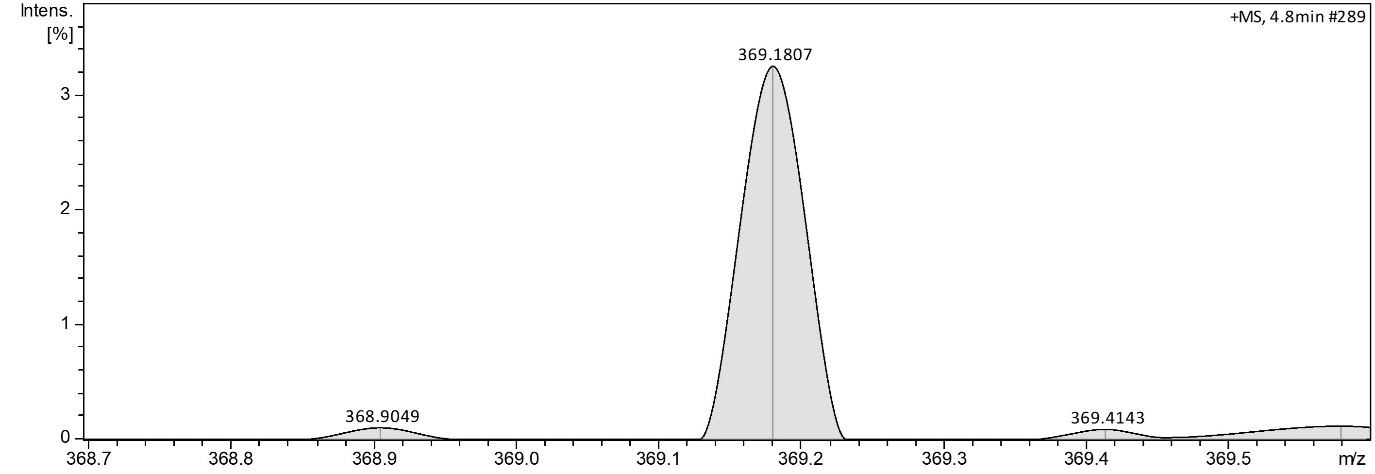


**Fig S1** c. Spectrum view of mitraphyline/ isomitraphyline/ strictosidine aglycone/ horhammericine/ dialdehyde 2


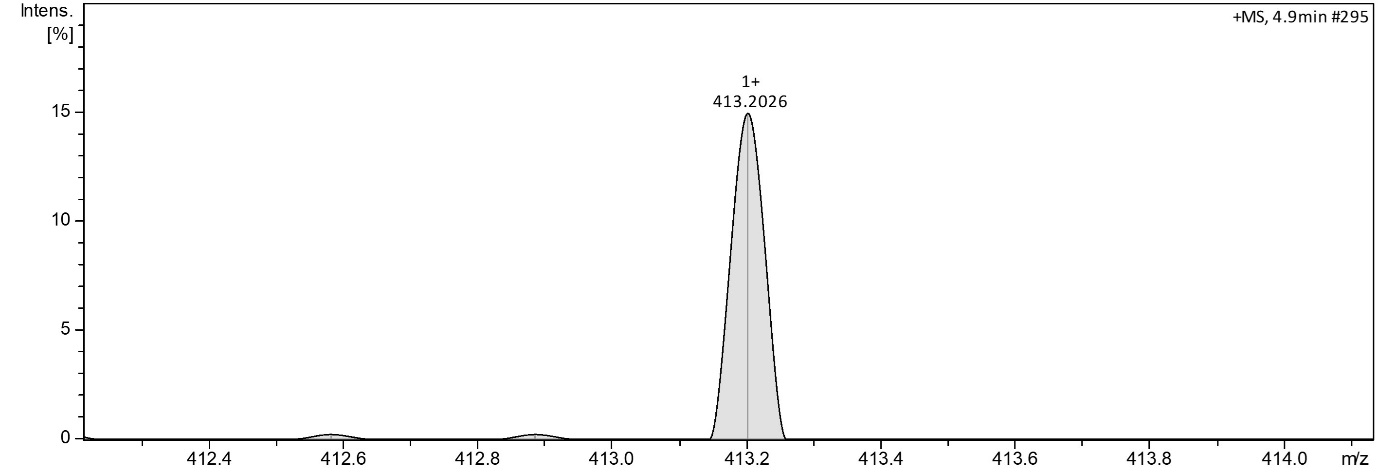


**Fig S1** d. Spectrum view of (-)-alstolucine A


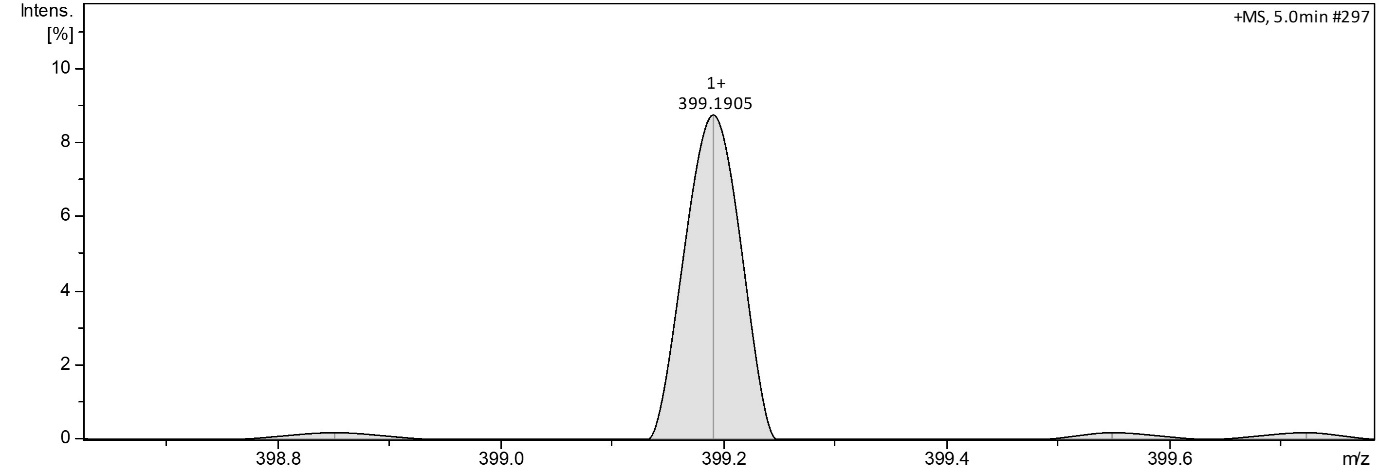


**Fig S1** e. Spectrum view of isospeciofoleine/ javaphilline 1


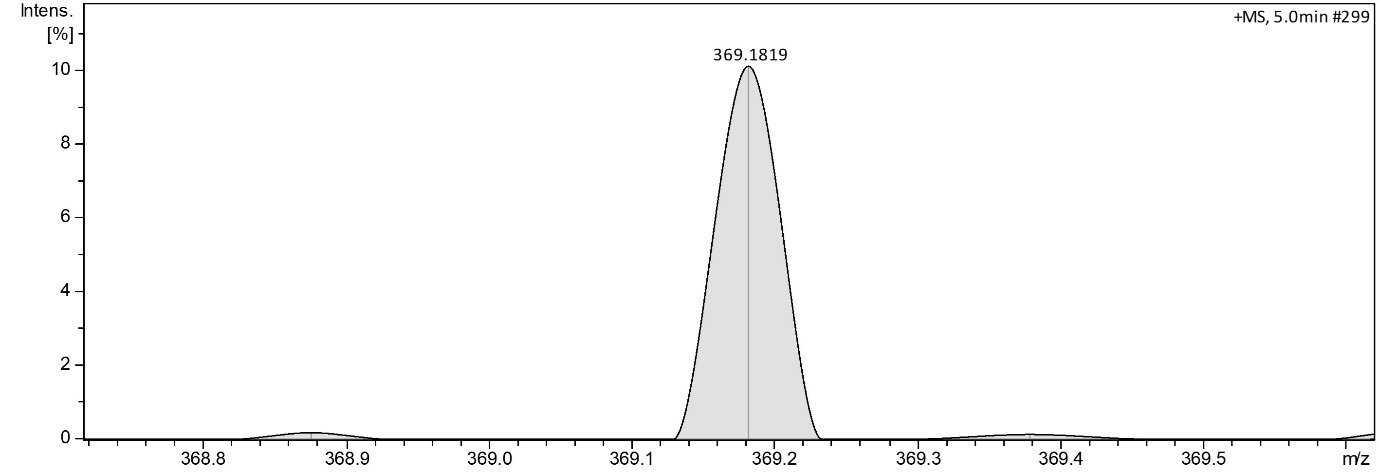


**Fig S1** f. Spectrum view of mitraphyline/ isomitraphyline/ strictosidine aglycone/ horhammericine/ dialdehyde 3


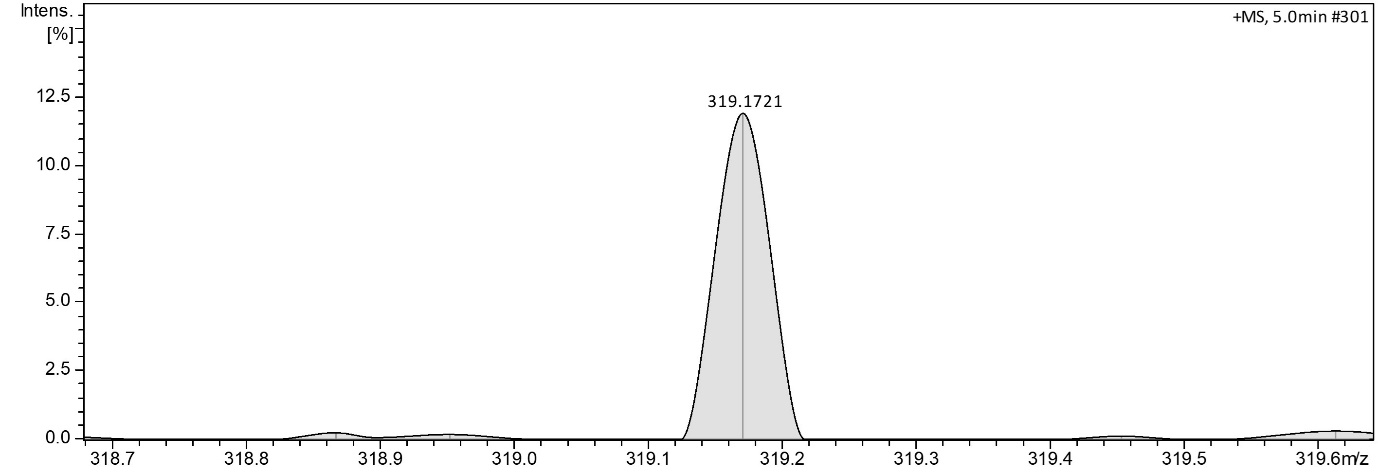


**Fig S1** g. Spectrum view of eseramine


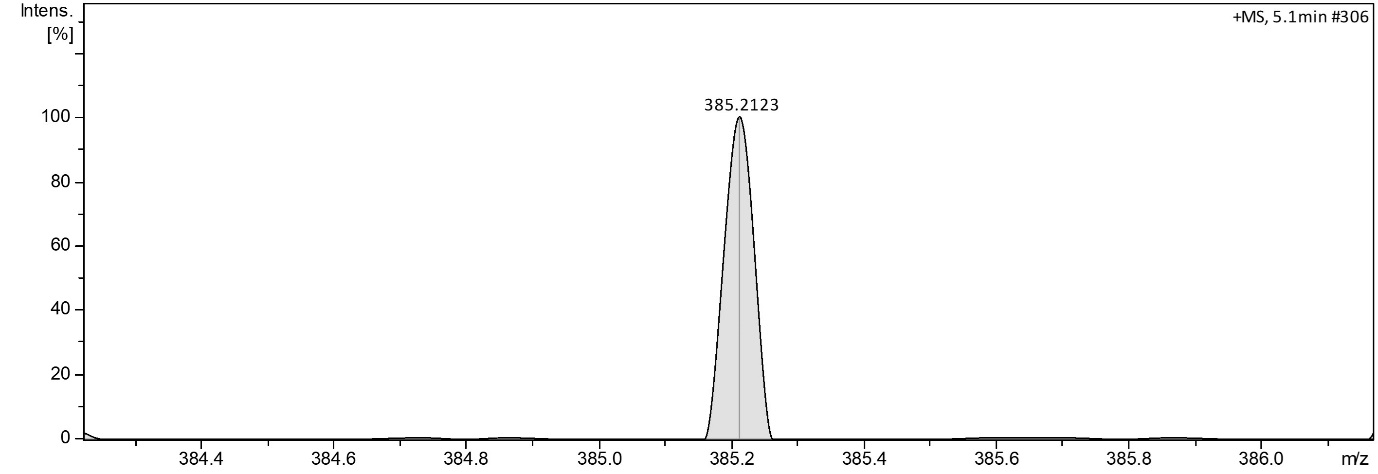


**Fig S1** h. Spectrum of isorynchophylline/ corynoxine/ corynoxine B


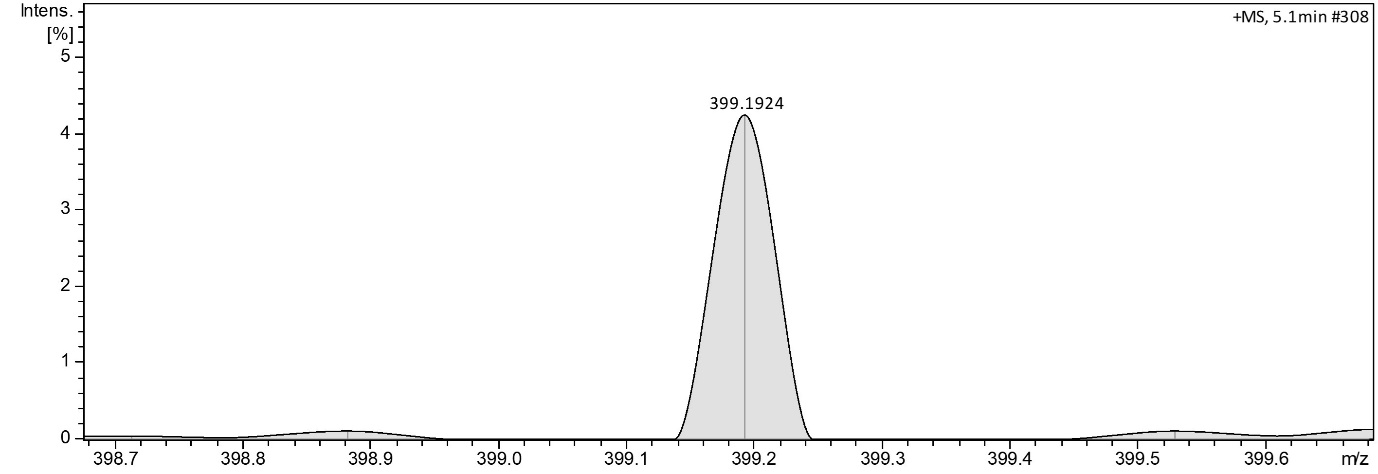


**Fig S1** i. Spectrum view of isospeciofoleine/ javaphilline 2


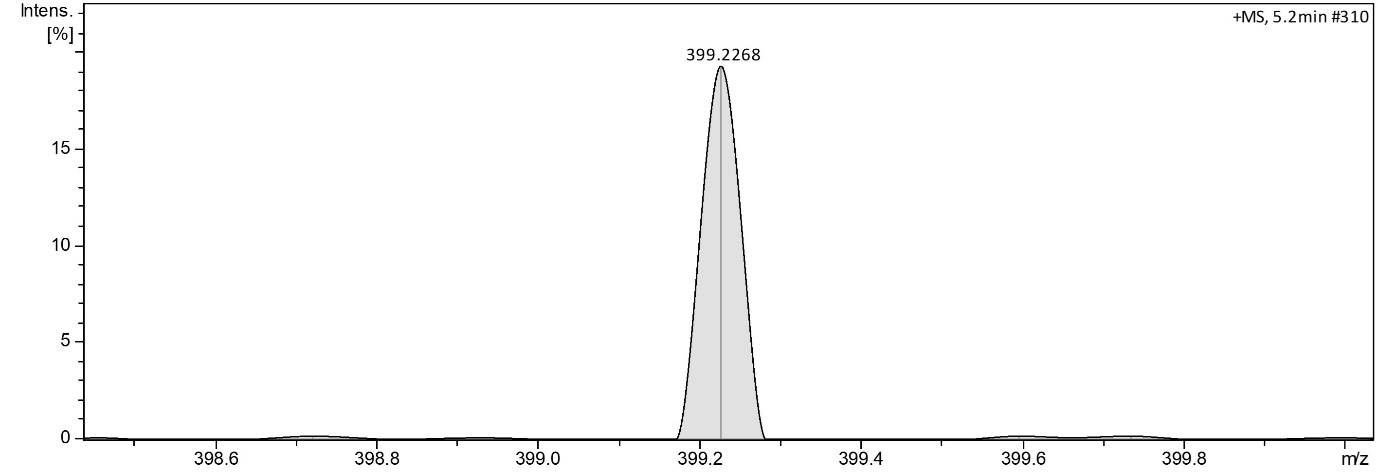


**Fig S1** j. Spectrum view of speciogynine


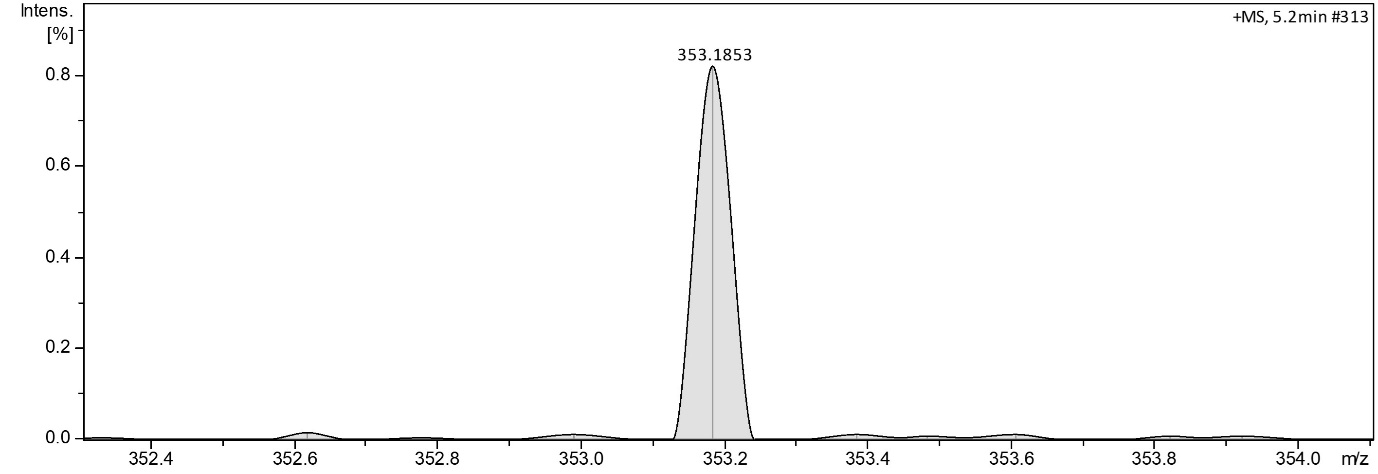


**Fig S1** k. Spectrum view of ajmalicine1


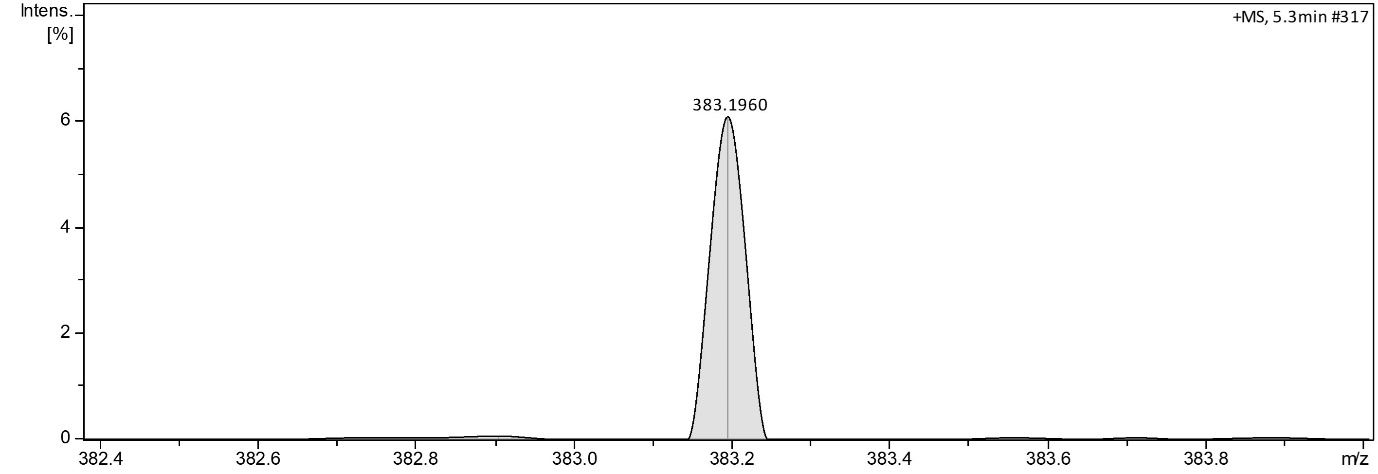


**Fig S1** l. Spectrum view of akuammine/ aricine/ cabucine/ lochnerinine


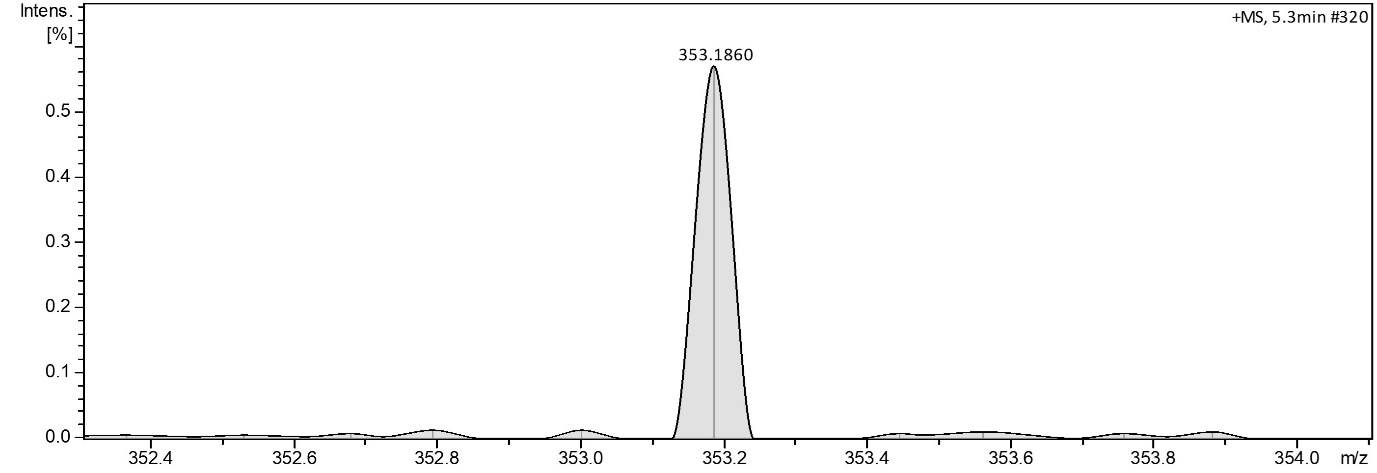


**Fig S1** m. Spectrum view of ajmalicine2


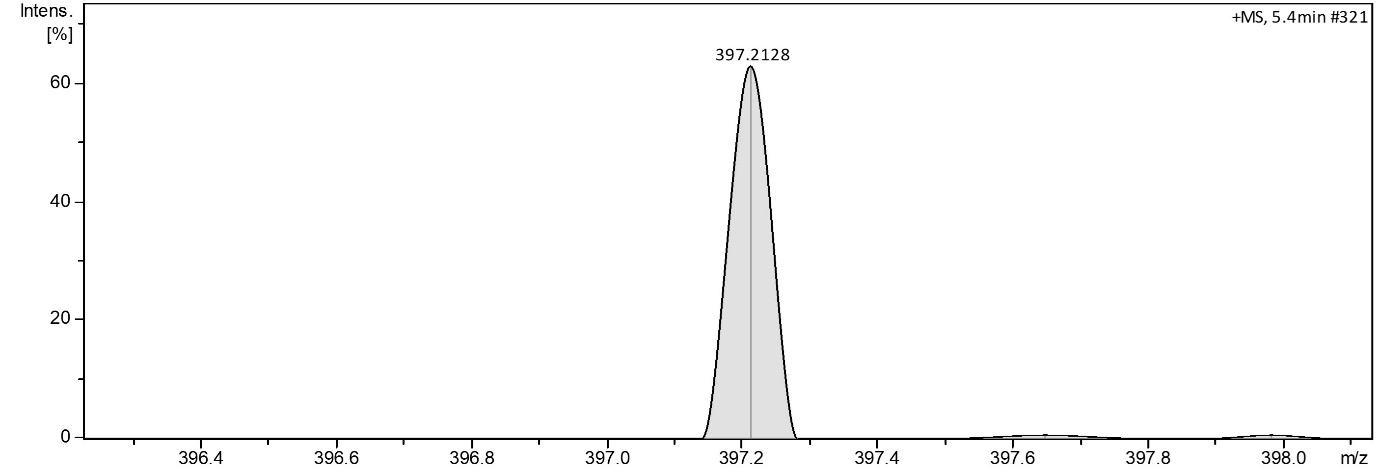


**Fig S1** n. Spectrum view of paynantheine


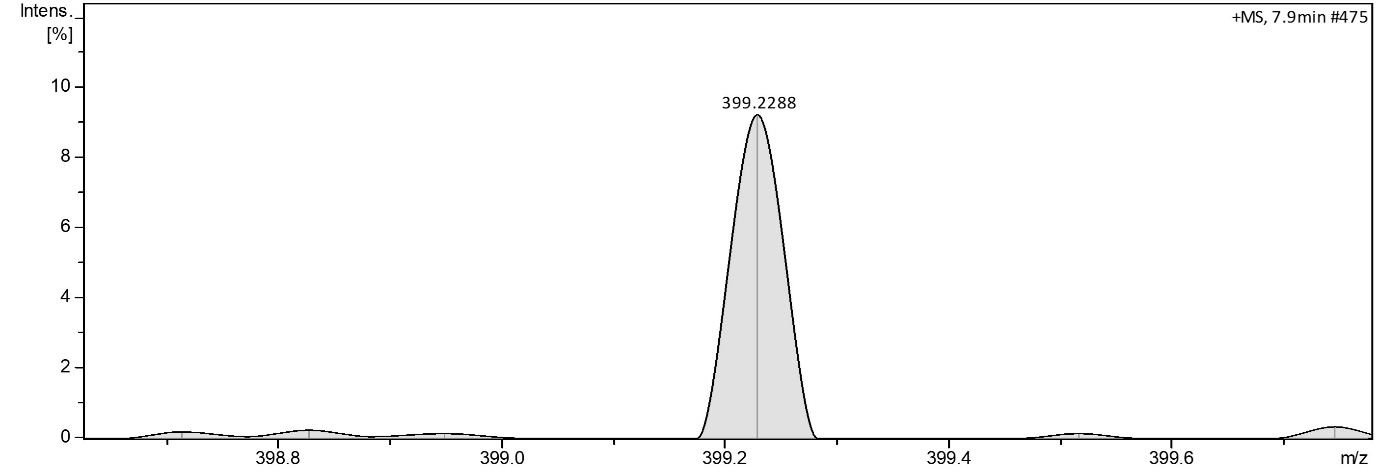


**Fig S1** o. Spectrum view of speciociliatine


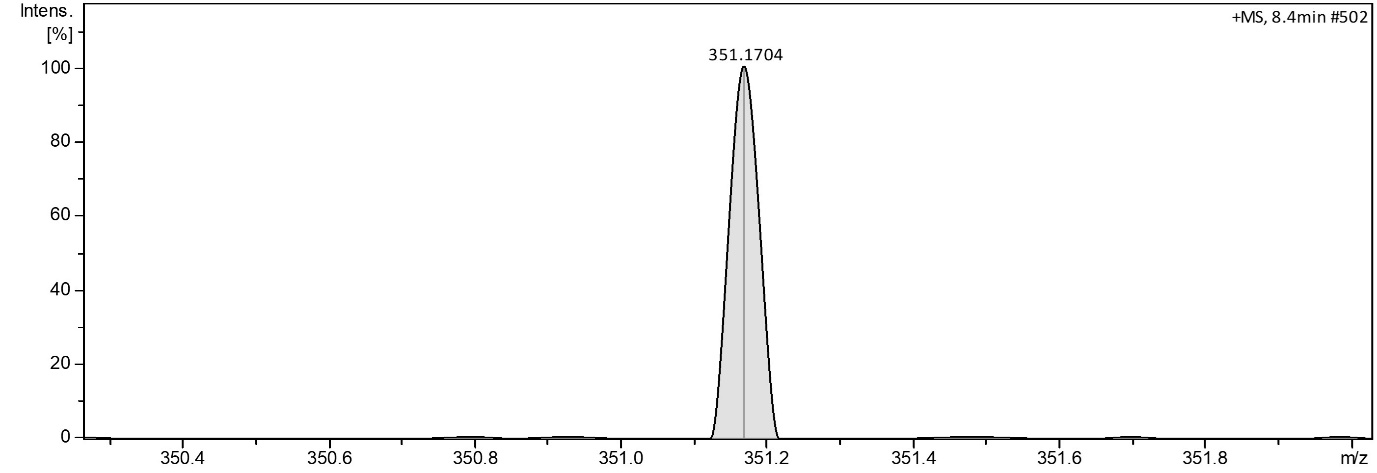


**Fig S1** p. Spectrum view of perakine/ vomilenine/ polyneuridine aldehyde/ 19-epi-cathenamine/ cathenamine


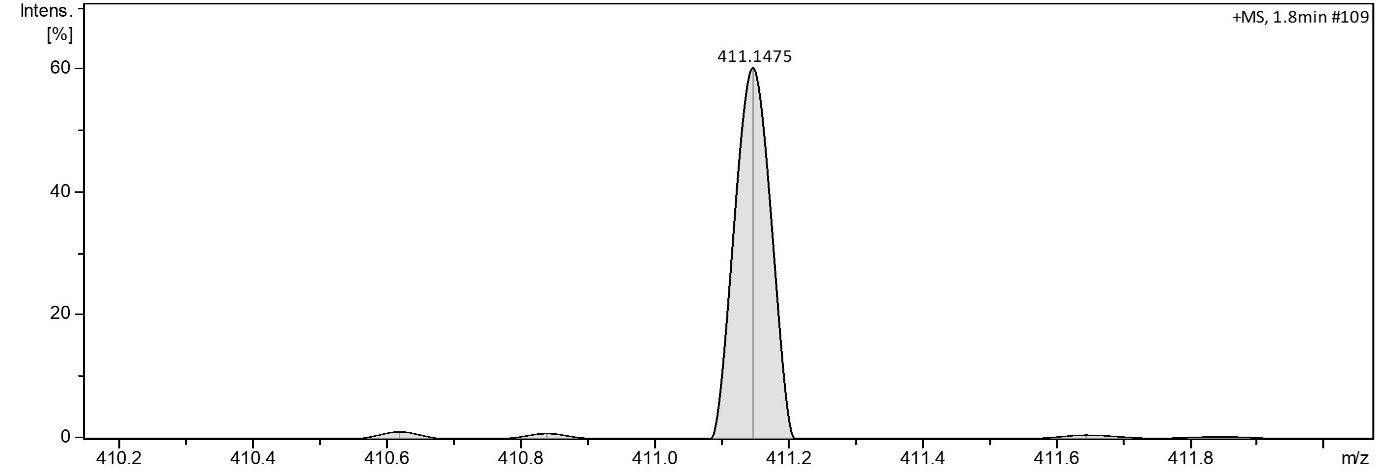


**Fig S1** q. Spectrum view of edulitine


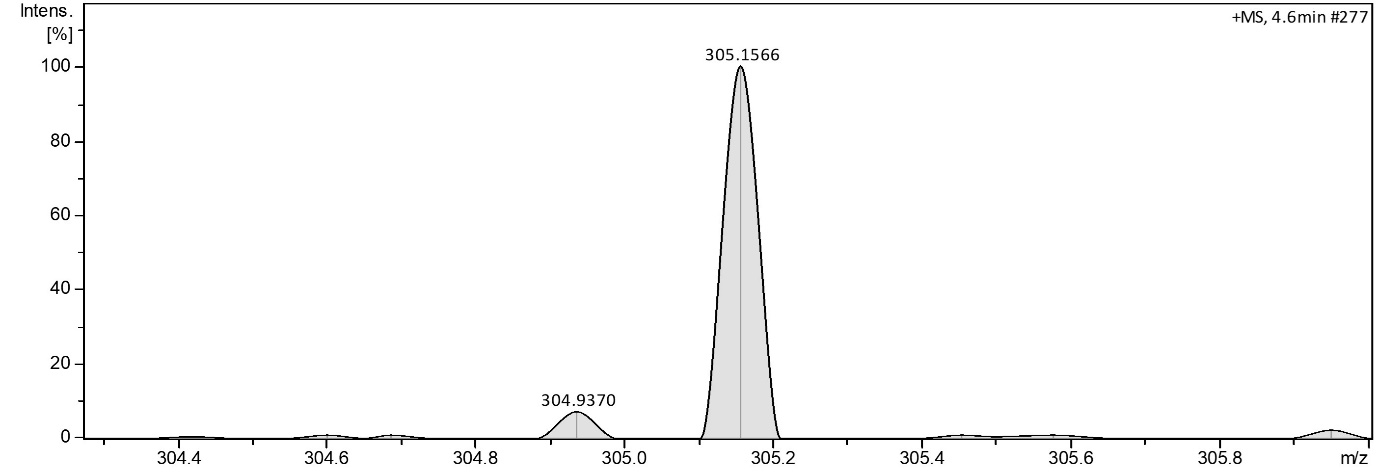


**Fig S1** r. Spectrum view of balfourodinium


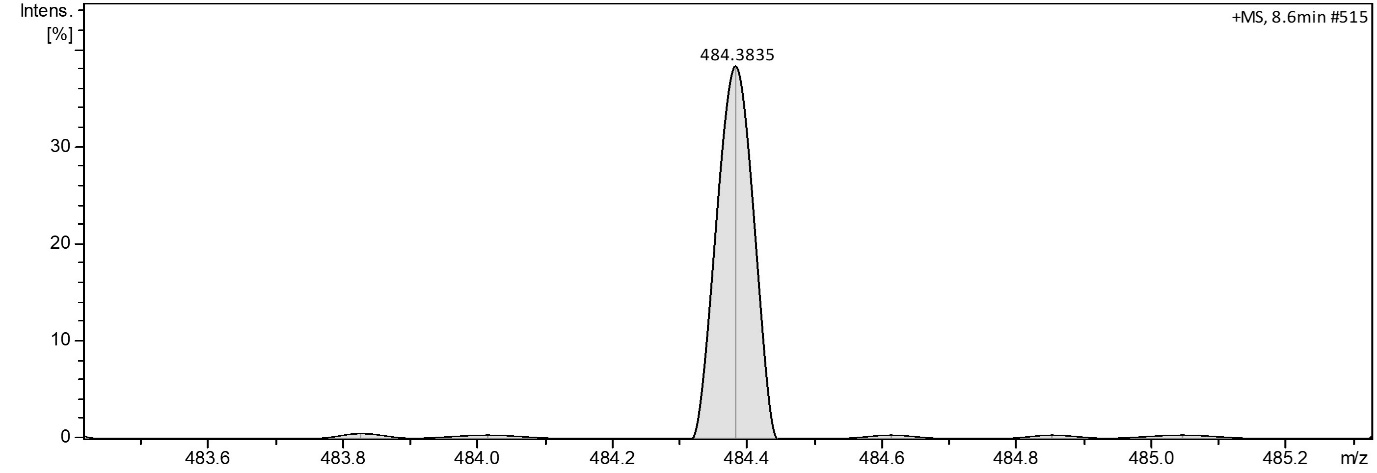


**Fig S1** s. Spectrum view of spirolucidine/oxolucidine B


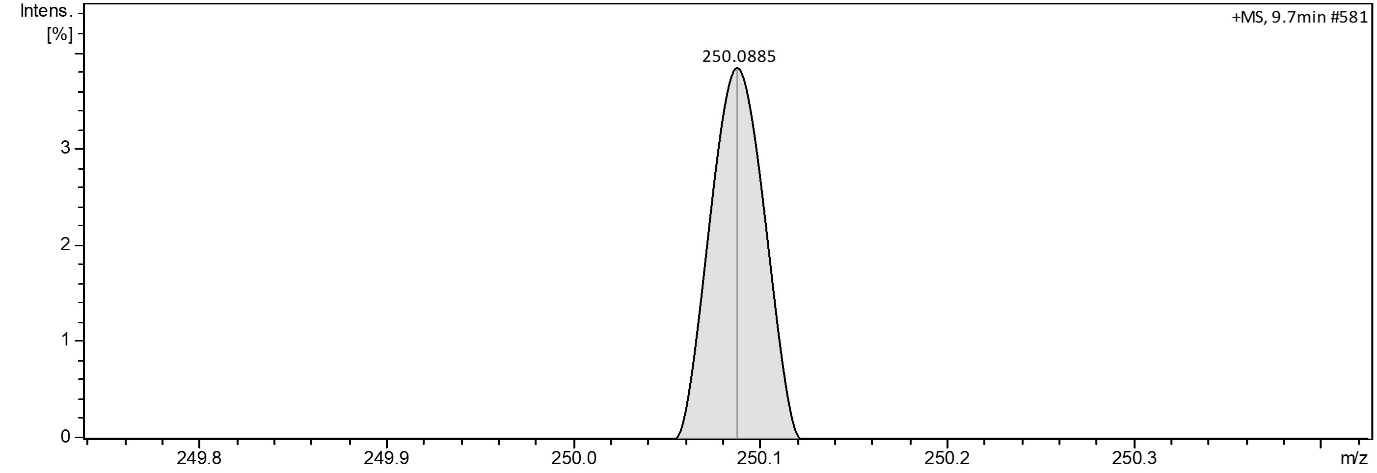


**Fig S1** t. Spectrum view of dubamine


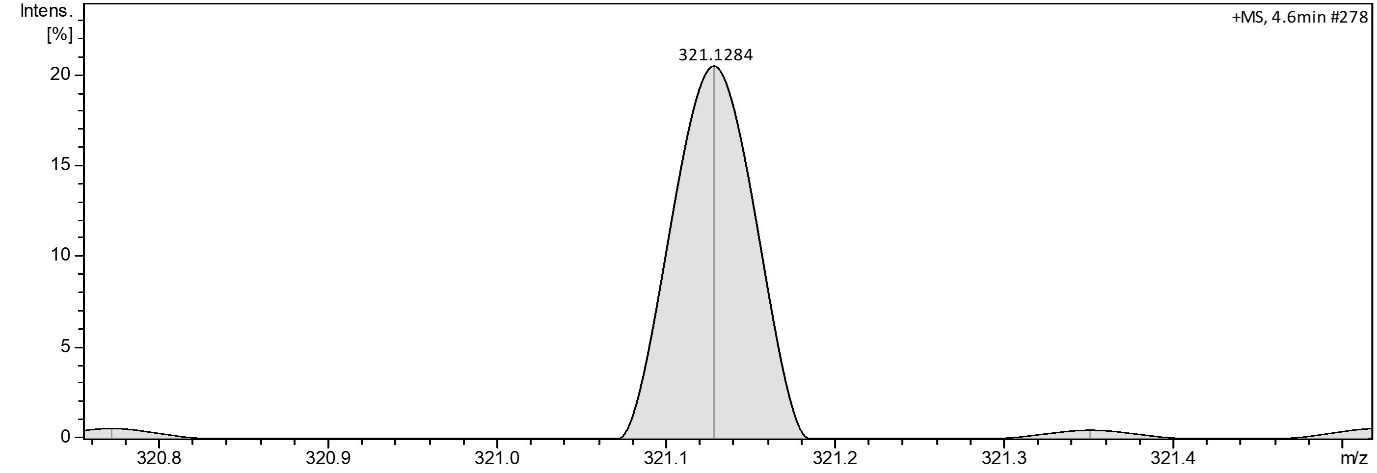


**Fig S1** u. Spectrum view of alangimarine


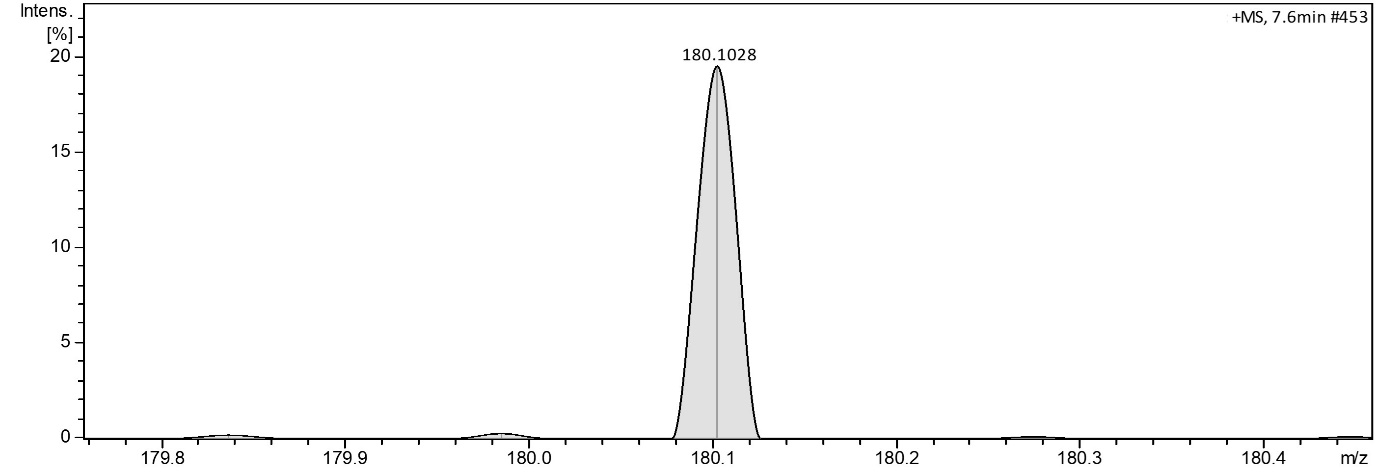


**Fig S1** v. Spectrum view of salsolinol


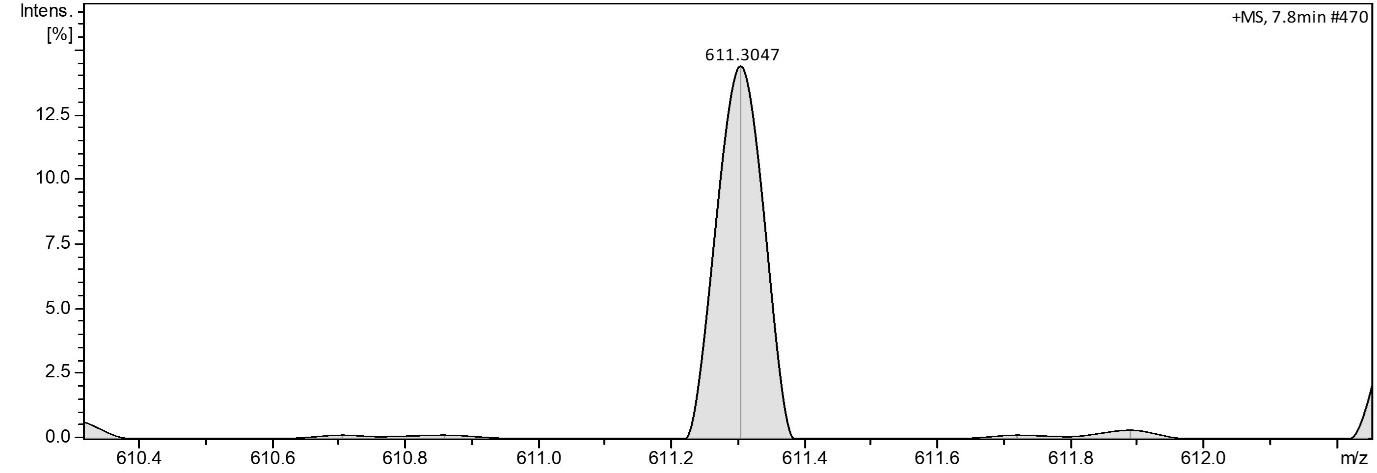


**Fig S1** w. Spectrum view of liensinine/ isoliensinine


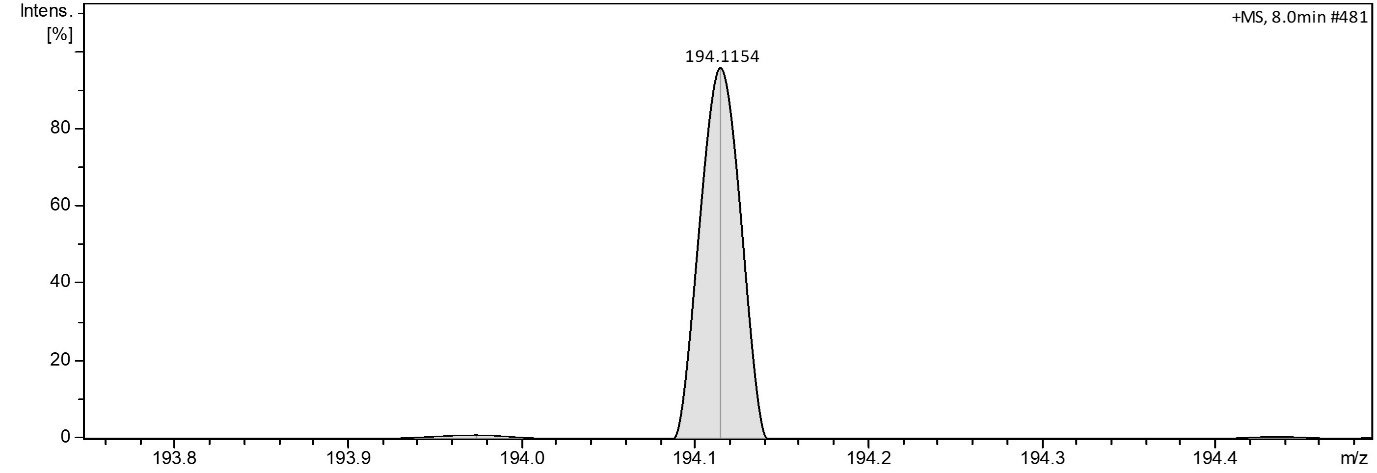


**Fig S1** x. Spectrum view of salsoline/ heliamine


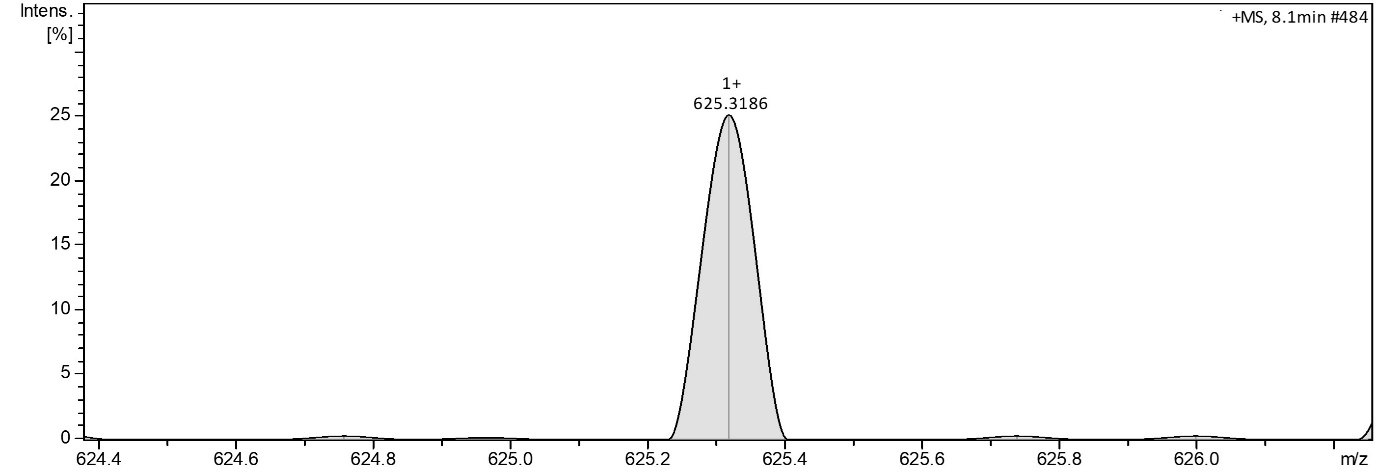


**Fig S1** y. Spectrum view of dauricine/ neferine


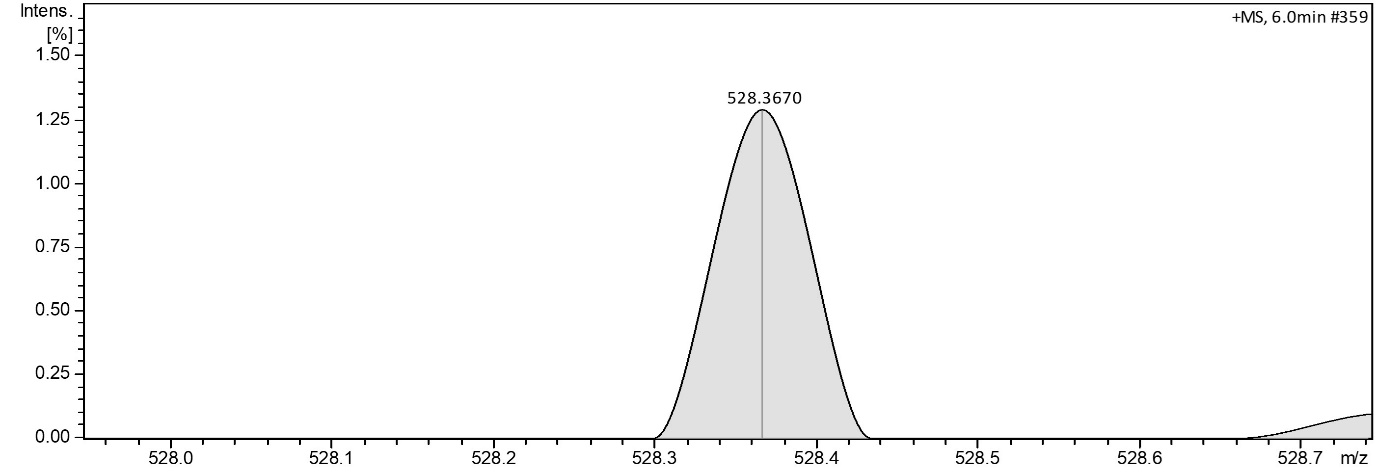


**Fig S1** z. Spectrum view of daphniphylline


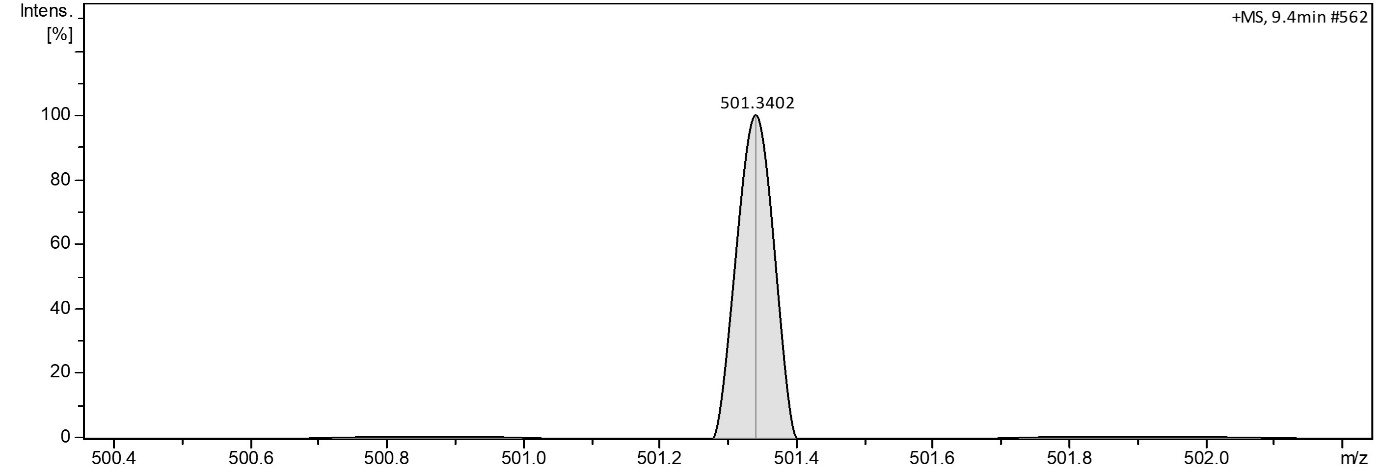


**Fig S1** aa. Spectrum view of adouetine X/ frangulanine


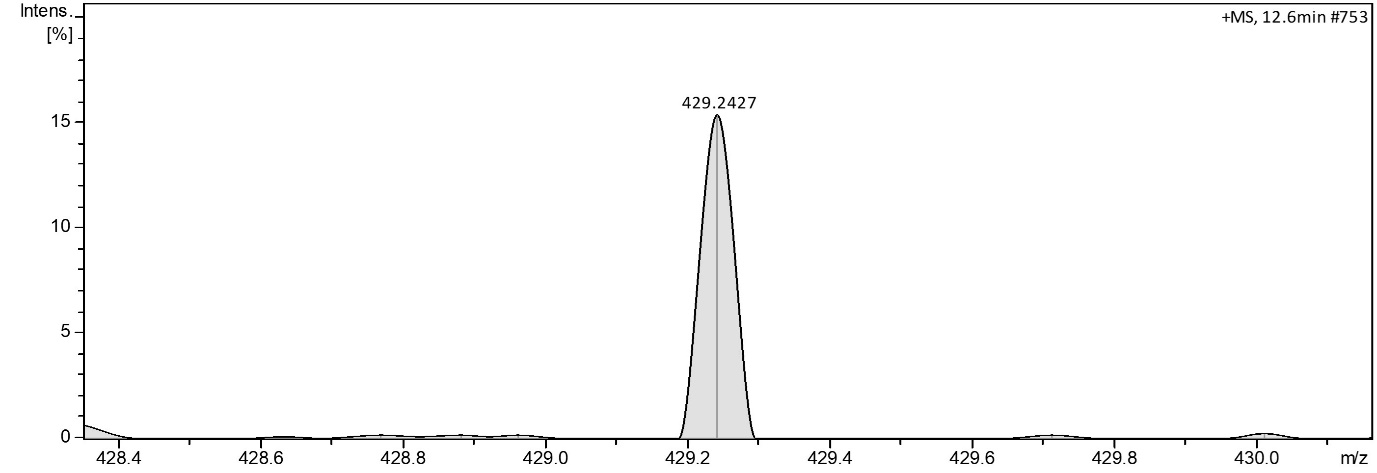


**Fig S1** bb. Spectrum view of nummularine F


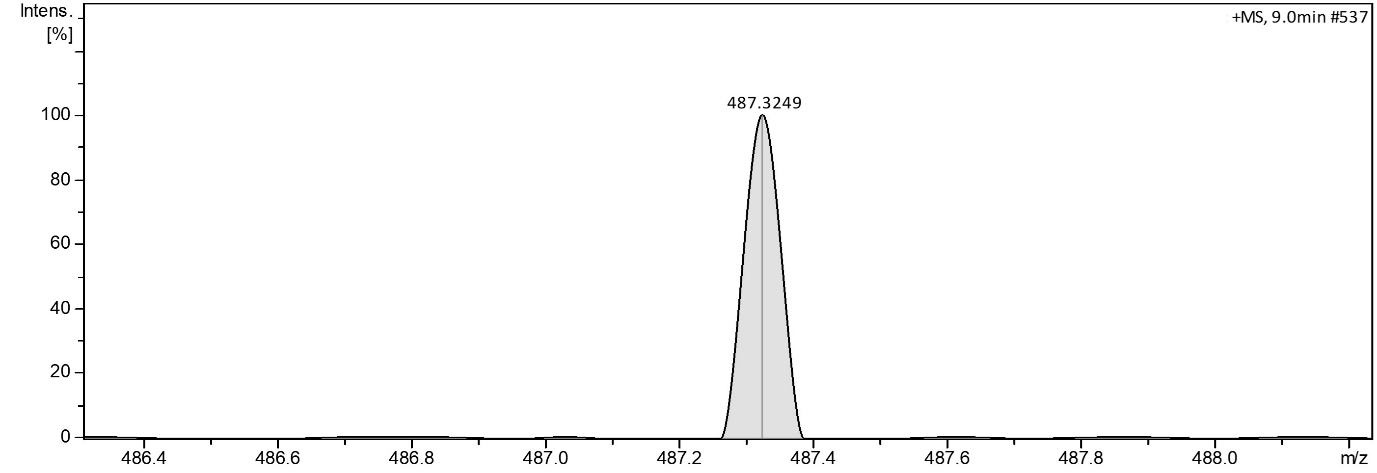


**Fig S1** cc. Spectrum view of Hovenine A


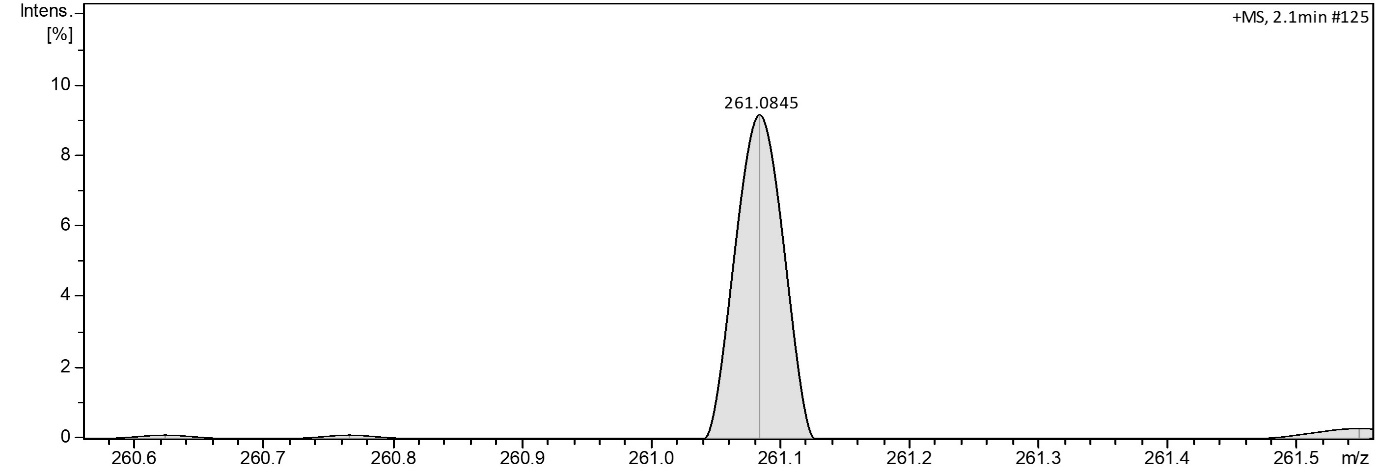


**Fig S1** dd. Spectrum view of 1,2,3,4-tetrahydro-β-carboline-1,3-dicarboxylic acid


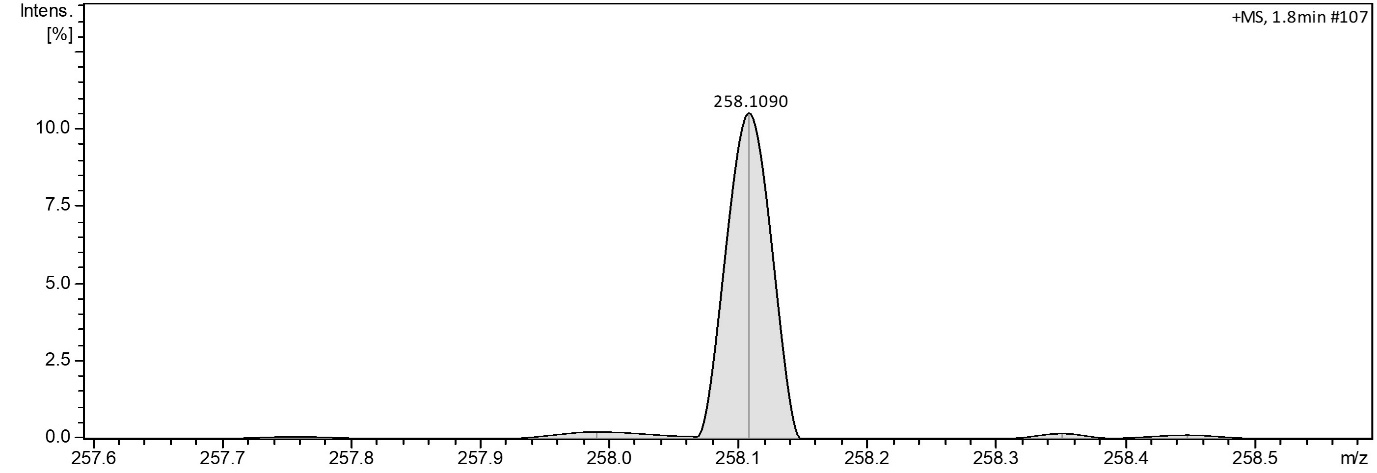


**Fig S1** ee. Spectrum view of ismine


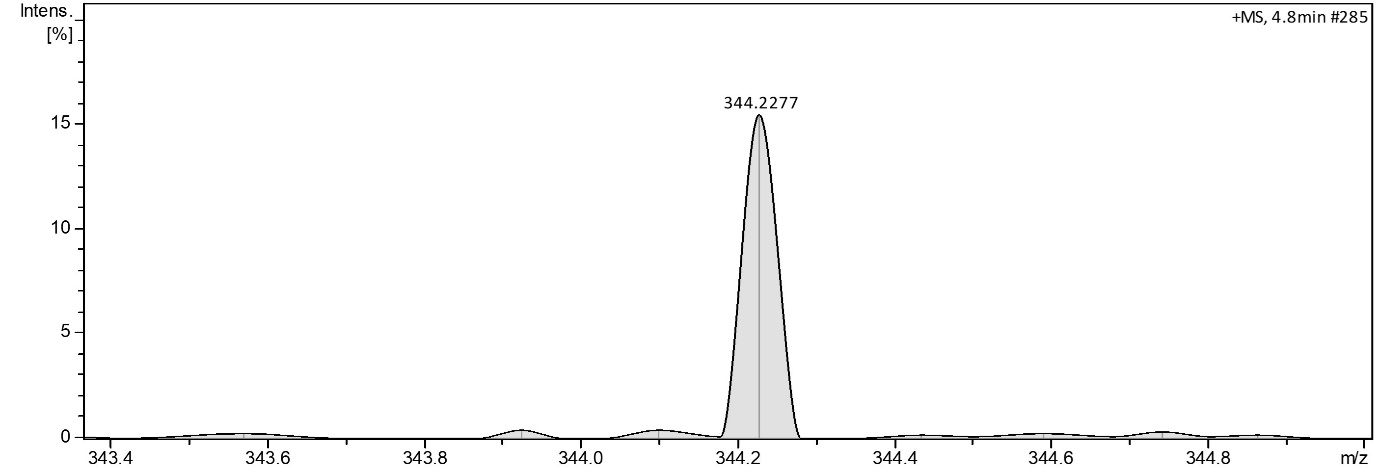


**Fig S1** ff. Spectrum view of piperolein B/ isopiperoleine B


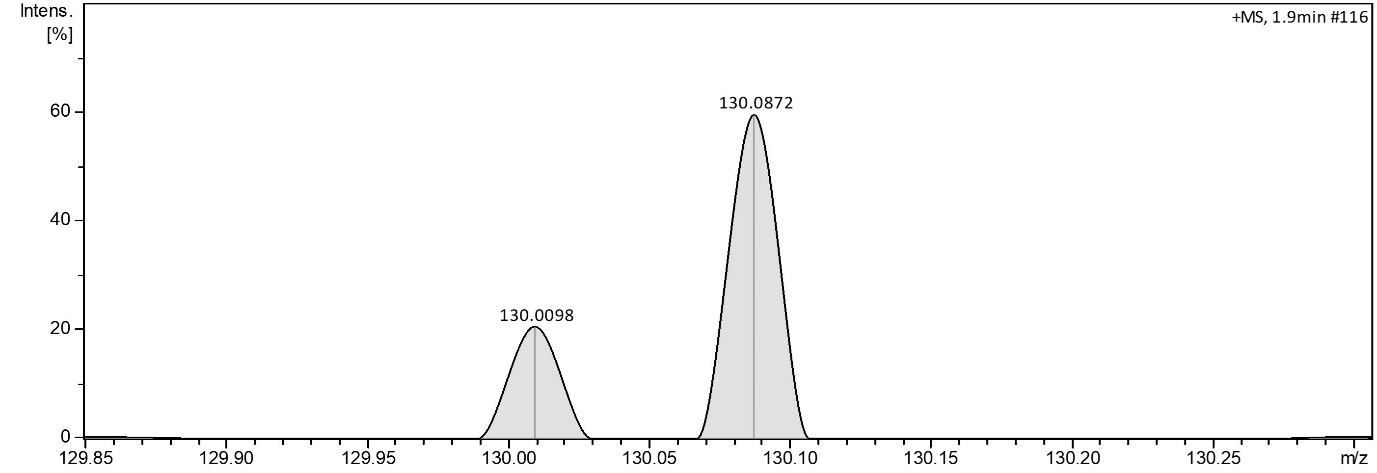


**Fig S1** gg. Spectrum view of L-pipecolic acid/D-pipecolic acid


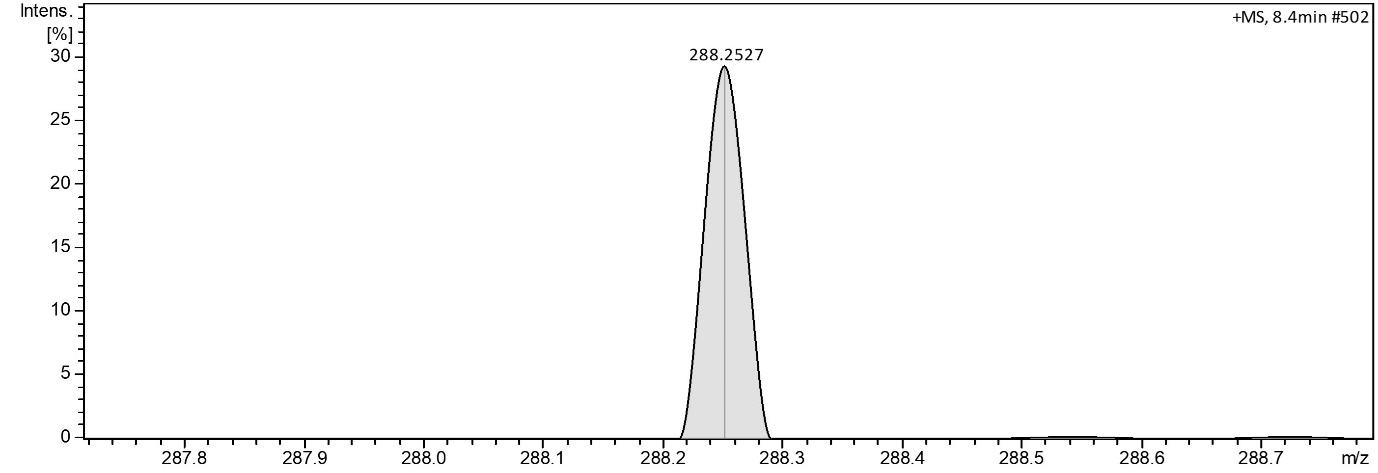


**Fig S1** hh. Spectrum view of prosopinine


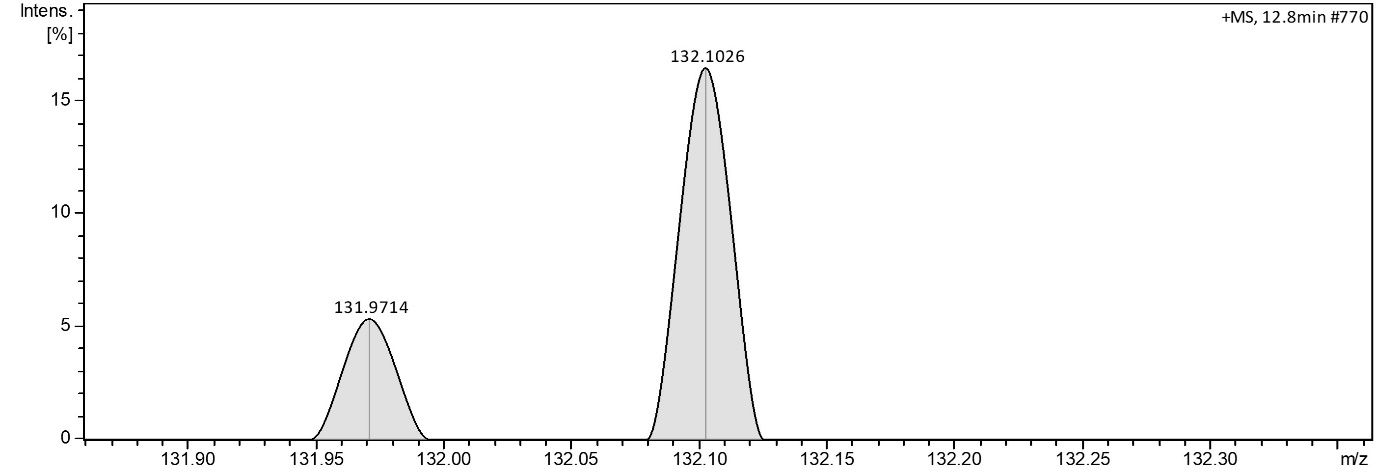


**Fig S1** ii. Spectrum view of 6-deoxyfagomine


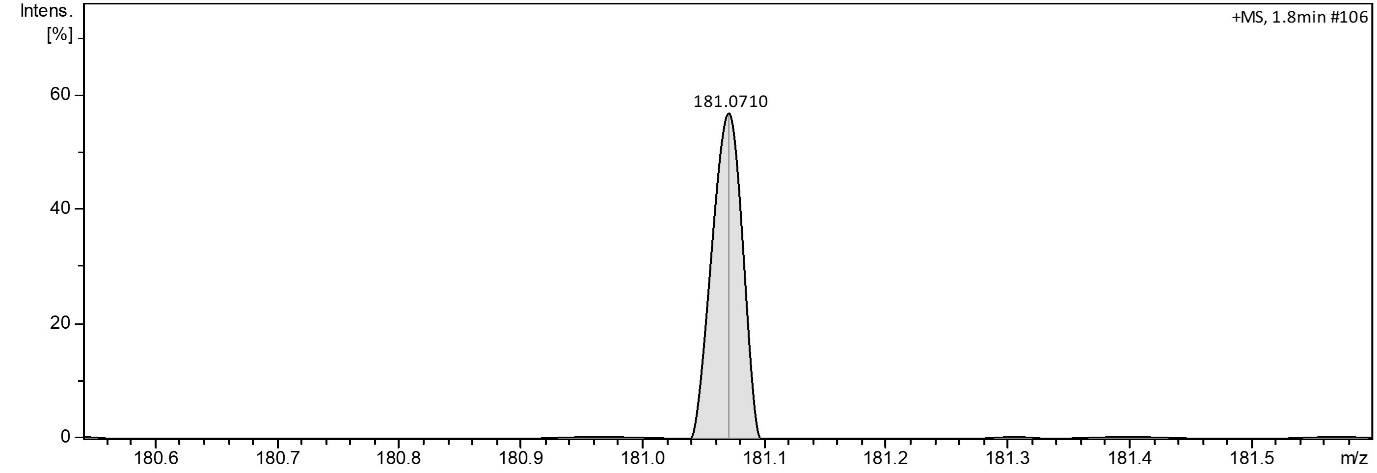


**Fig S1** jj. Spectrum view of theophylline/ theobromine/ paraxanthine


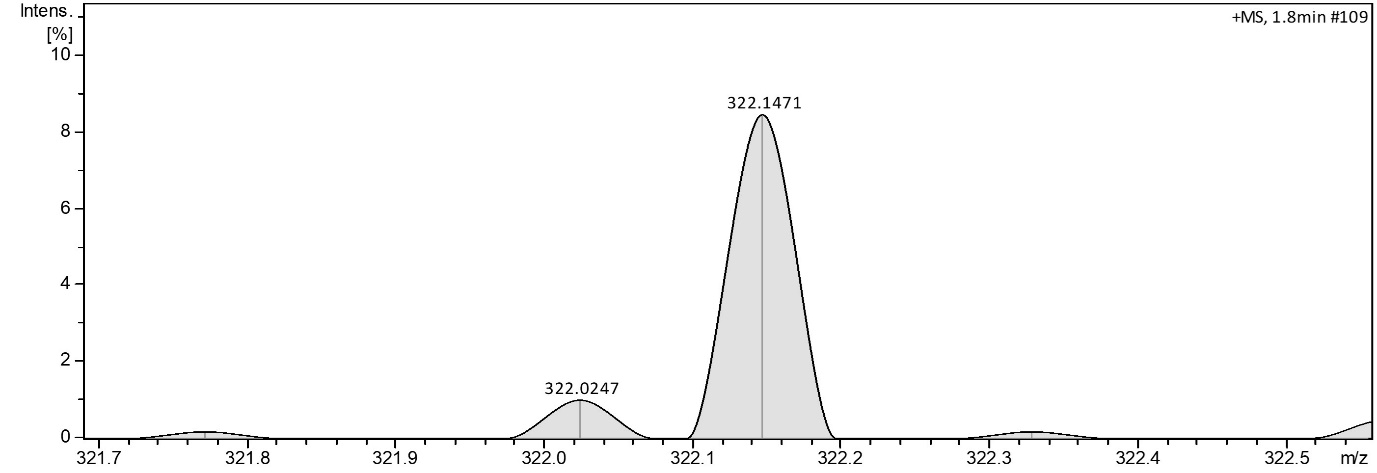


**Fig S1** kk. Spectrum view of acrocynine/2-[4(3,4-methylenedioxyphenyl)butyl]-4(1H)-quinoline


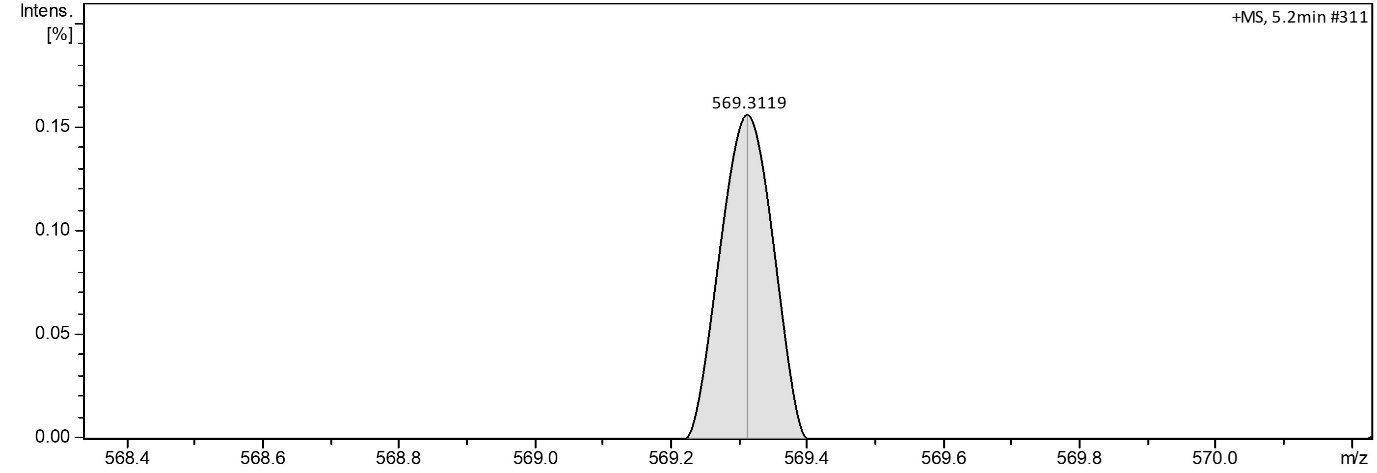


**Fig S1** ll. Spectrum view of ceanothine E/adouetine Y


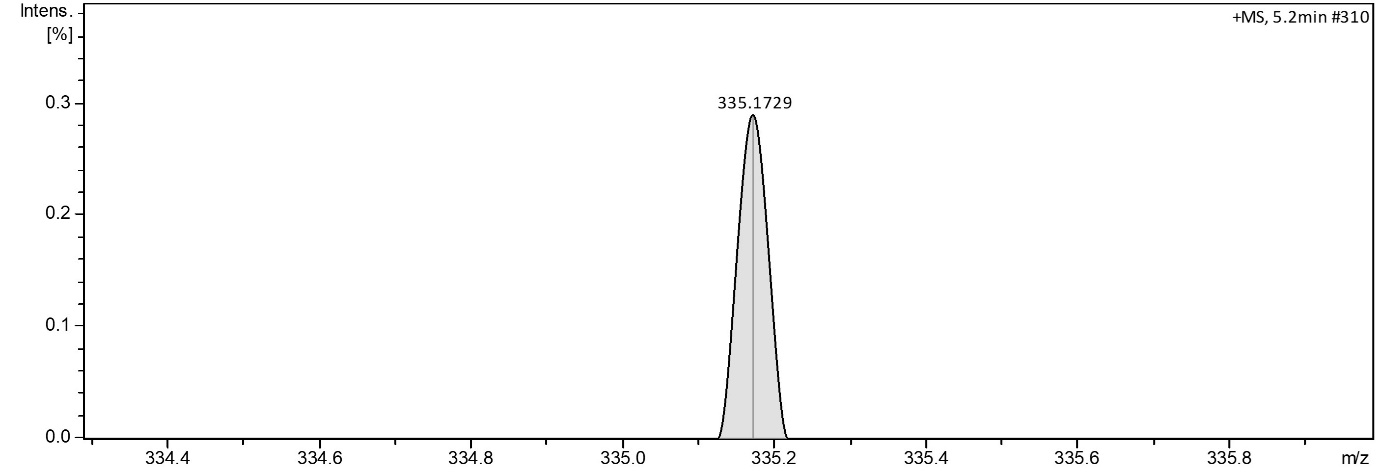


**Fig S1** mm. Spectrum view of strychnine/vinorine


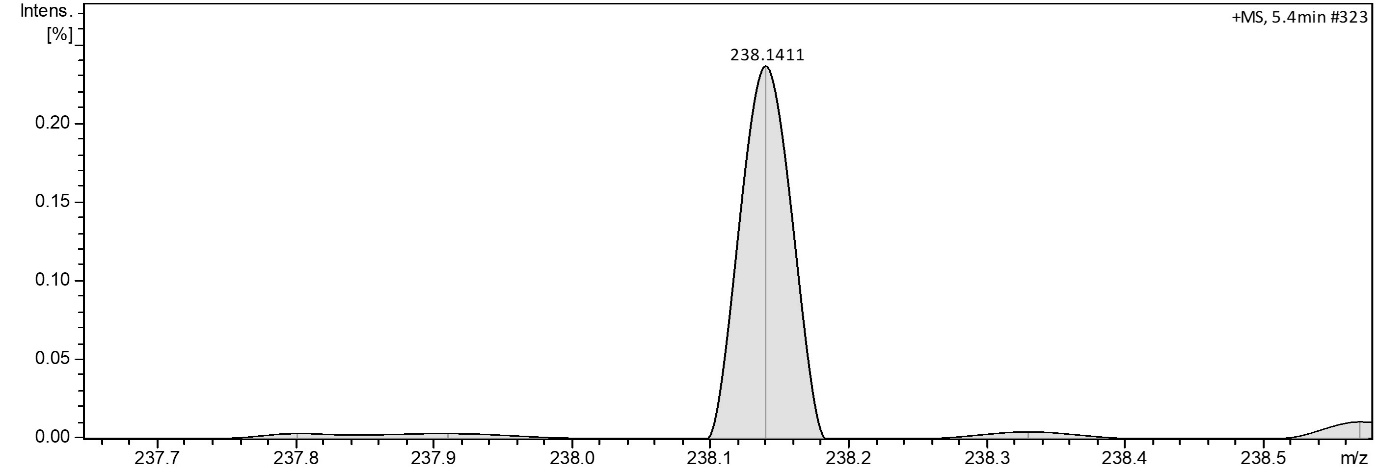


**Fig S1** nn. Spectrum view of anhalonine/pellotine/gigantine/O-7-angelylheliotridine


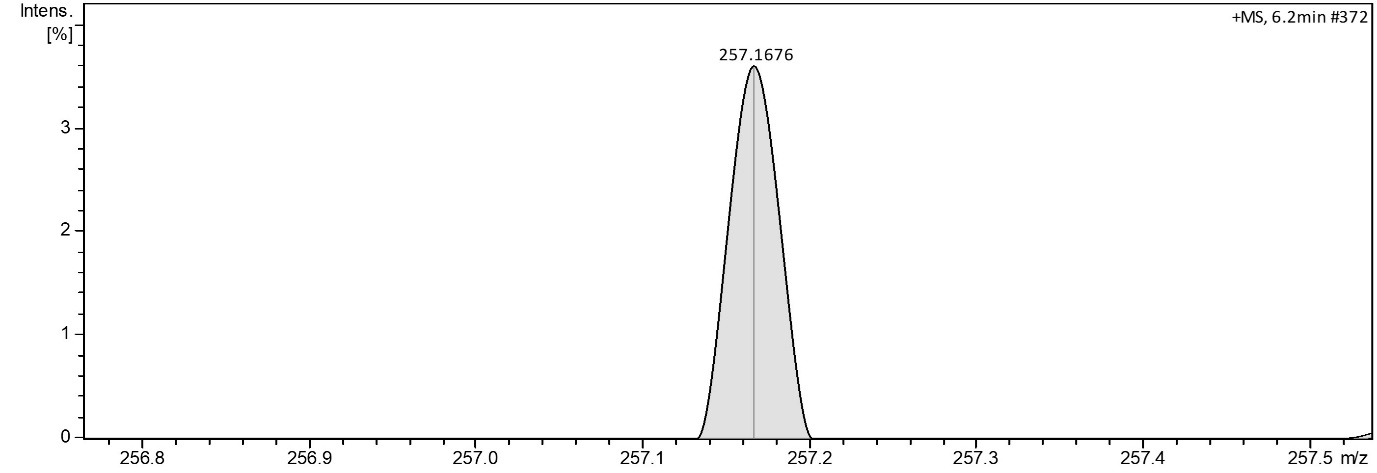


**Fig S1** oo. Spectrum view of huperzine/chanoclavine-I/fumigaclavine


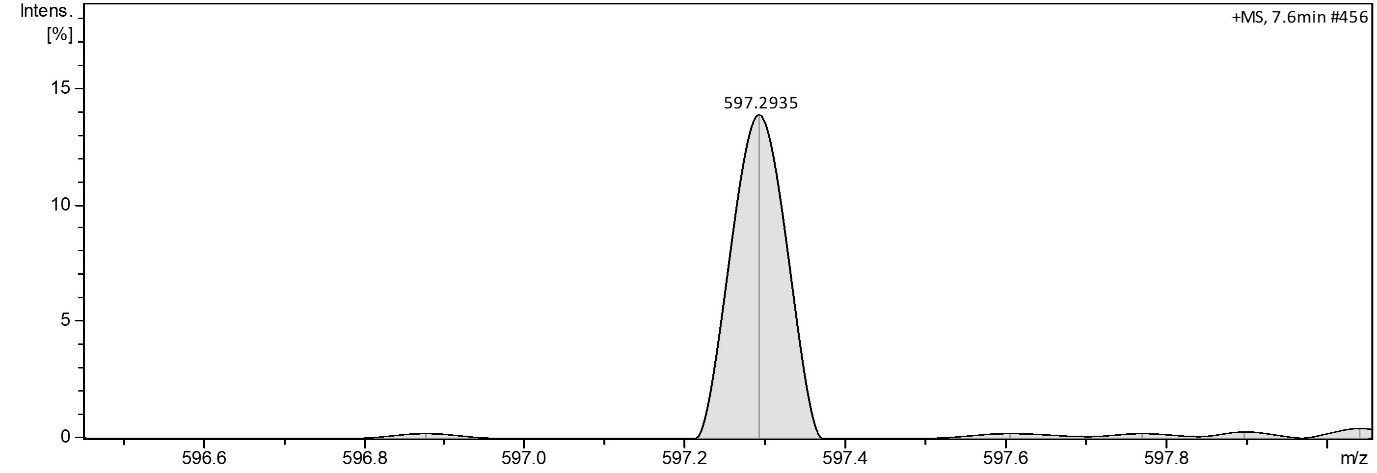


**Fig S1** pp. Spectrum view of guattegaumerine/dipiperamide D/berbamunine


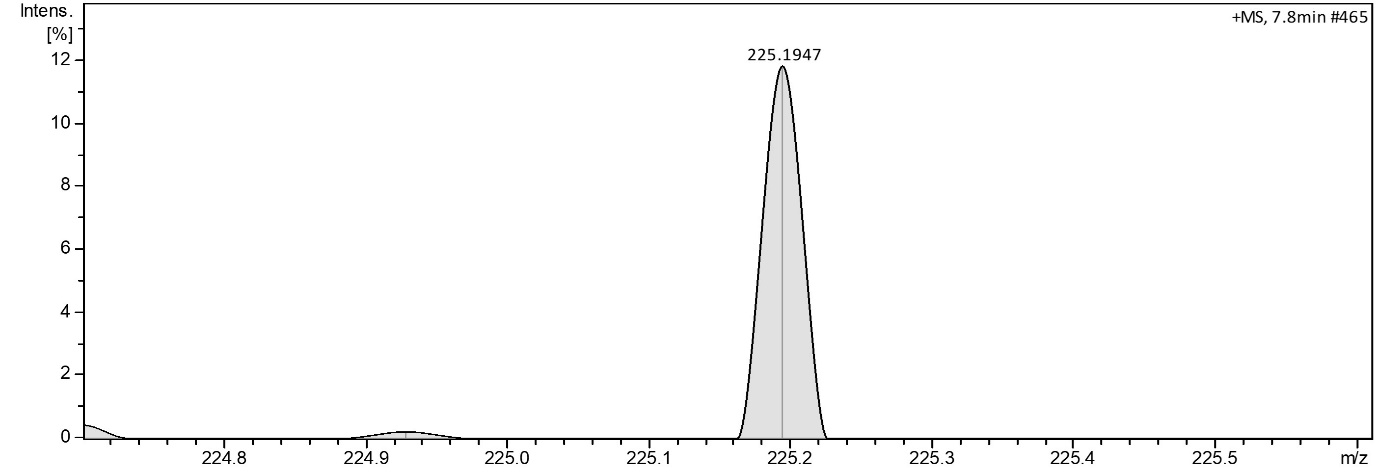


**Fig S1** qq. Spectrum view of anapheline/cuscohygrine


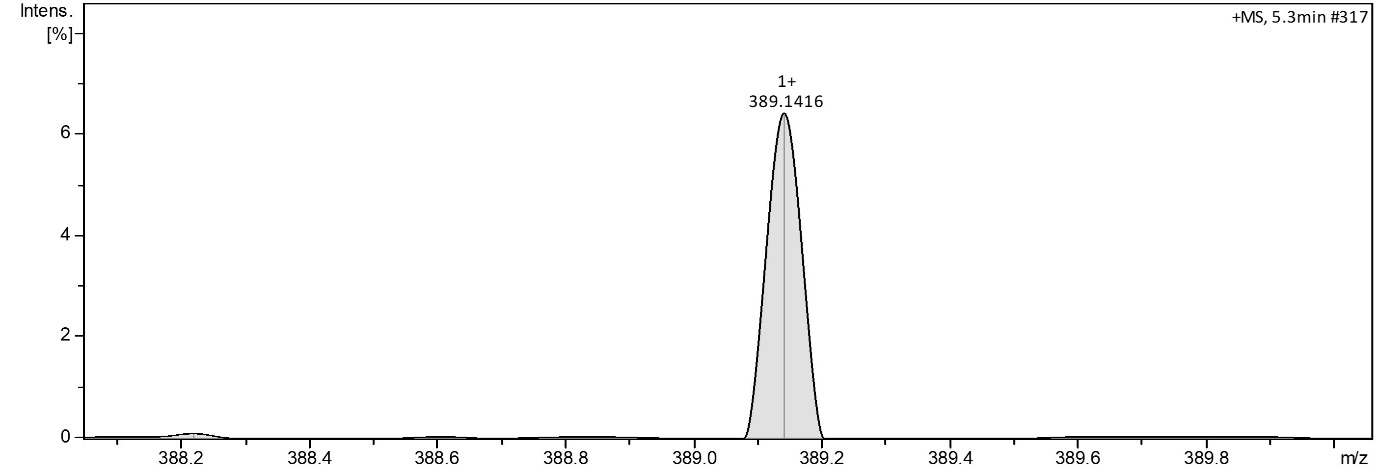


**Fig S1** rr. Spectrum view of secologanin


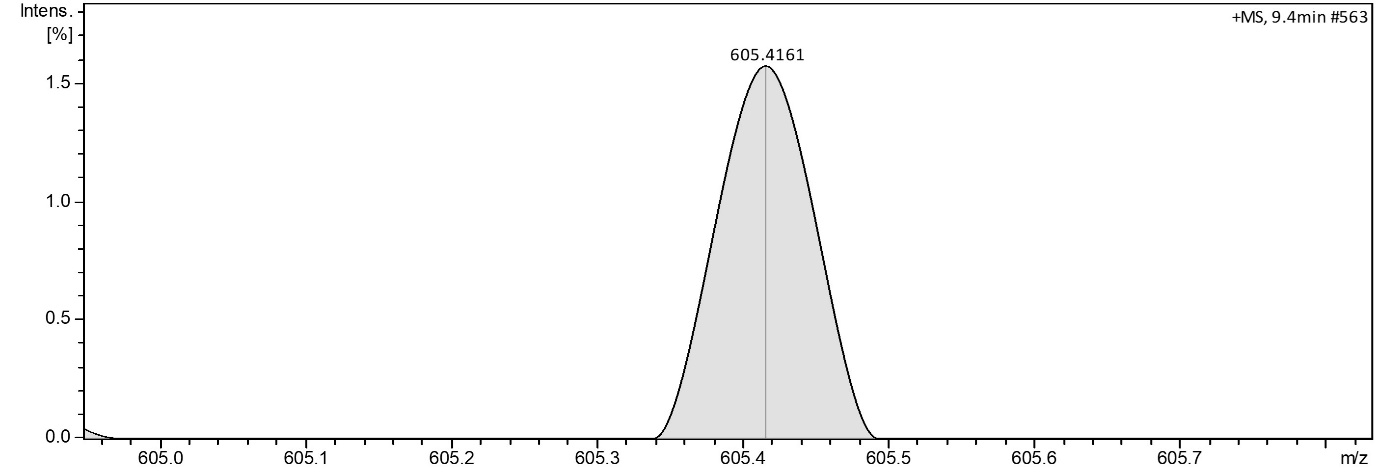


**Fig S1** ss. Spectrum view of papyriferic acid
