# Supplementary material for: Comparative metabolomics analysis reveals alkaloid repertoires in young and mature Mitragyna speciosa (Korth.) Havil. Leaves
Source: PLoS One. 2023 Mar 21;18(3):e0283147. doi: 10.1371/journal.pone.0283147 (PMC10030037; doi:10.1371/journal.pone.0283147)
Supplement: S2 Appendix — (DOCX) [file pone.0283147.s010.docx]

**S2 Appendix.** LC-ESI-TOF-MS/MS spectra of metabolite features putatively identified at ID level 2 (identification done at fragmentation level (MS2) by matching with online databases).

**Fig S2** a. LC-ESI-TOF-MS/MS spectra for tryptamine

**Fig S2** b. LC-ESI-TOF-MS/MS spectra for yohimbine

**Fig S2** c. LC-ESI-TOF-MS/MS spectra for akuammidine

**Fig S2** d. LC-ESI-TOF-MS/MS spectra for strictosidine

**Fig S2** e. LC-ESI-TOF-MS/MS spectra for speciofoline

**Fig S2** f. LC-ESI-TOF-MS/MS spectra for 7-hydroxymitragynine

**Fig S2** g. LC-ESI-TOF-MS/MS spectra for rynchophylline

**Fig S2** h. LC-ESI-TOF-MS/MS spectra for corynantheidine

**Fig S2** i. LC-ESI-TOF-MS/MS spectra for 11-methoxyvinorine

**Fig S2** j. LC-ESI-TOF-MS/MS spectra for hirsuteine

**Fig S2** k. LC-ESI-TOF-MS/MS spectra for mitragynine

**Fig S2** l. LC-ESI-TOF-MS/MS spectra for isopaynantheine

**Fig S2** m. LC-ESI-TOF-MS/MS spectra for brucine

**Fig S2** n. LC-ESI-TOF-MS/MS spectra for alstonine

**Fig S2** o. LC-ESI-TOF-MS/MS spectra for vomicine

**Fig S2** p. LC-ESI-TOF-MS/MS spectra for (+)-N-(methoxycarbonyl)-N-norboldine

**Fig S2** q. LC-ESI-TOF-MS/MS spectra for cinegalline

**Fig S2** r. LC-ESI-TOF-MS/MS spectra for vasicinol

**Fig S2** s. LC-ESI-TOF-MS/MS spectra for caffeine

**Fig S2** t. LC-ESI-TOF-MS/MS spectra for lumichrome

**Fig S2** u. LC-ESI-TOF-MS/MS spectra for quinate

**Fig S2** v. LC-ESI-TOF-MS/MS spectra for 5''-O-beta-D-glucosylpyridoxine

**Fig S2** w. LC-ESI-TOF-MS/MS spectra for 3-Methoxytyramine-betaxanthin

**Fig S2** x. LC-ESI-TOF-MS/MS spectra for caffeic aldehyde

**Fig S2** y. LC-ESI-TOF-MS/MS spectra for sinapate

**Fig S2** z. LC-ESI-TOF-MS/MS spectra for magnoshinin

**Fig S2** aa. LC-ESI-TOF-MS/MS spectra for glaucarubinone/soularubinone

**Fig S2** bb. LC-ESI-TOF-MS/MS spectra for 3-Oxoglycyrrhetinate

**Fig S2** cc. LC-ESI-TOF-MS/MS spectra for glabric acid

**Fig S2** dd. LC-ESI-TOF-MS/MS spectra for fatsicarpain C

**Fig S2** ee. LC-ESI-TOF-MS/MS spectra for epicatechin

**Fig S2** ff. LC-ESI-TOF-MS/MS spectra for quarcetin/6-hydroxykaempferol/ robinetin/morin/8-hydroxykaempferol

**Fig S2** gg. LC-ESI-TOF-MS/MS spectra for rutin

**Fig S2** hh. LC-ESI-TOF-MS/MS spectra for procyanidin B4/procyanidin B5

**Fig S2** ii. LC-ESI-TOF-MS/MS spectra for 2''-Hydroxygenistein/orobol

**Fig S2** jj. LC-ESI-TOF-MS/MS spectra for isoorientin 2''-O-rhamnoside

**Fig S2** kk. LC-ESI-TOF-MS/MS spectra for quarcetin/6-hydroxykaempferol/ robinetin/morin/8-hydroxykaempferol

**Fig S2** ll. LC-ESI-TOF-MS/MS spectra for myricitrin/bracteatin 6-O-glucoside/gossypetin 8-rhamnoside

**Fig S2** mm. LC-ESI-TOF-MS/MS spectra for carthamone

**Fig S2** nn. LC-ESI-TOF-MS/MS spectra for cinchonain 1a

**Fig S2** oo. LC-ESI-TOF-MS/MS spectra for 3,4-dihydroxybenzaldehyde
